# Patterns of emergency department presentations for a youth mental health cohort: data-linkage cohort study

**DOI:** 10.1192/bjo.2023.521

**Published:** 2023-09-14

**Authors:** Frank Iorfino, Catherine McHugh, Matthew Richards, Adam Skinner, Ante Prodan, Jo-an Occhipinti, Yun Ju Christine Song, Simon Chiu, Simon Judkins, Elizabeth Scott, Ian B. Hickie

**Affiliations:** Brain and Mind Centre, University of Sydney, Sydney, New South Wales, Australia; Brain and Mind Centre, University of Sydney, Sydney, New South Wales, Australia; Translational Health Research Institute, Western Sydney University, Sydney, New South Wales, Australia; and School of Computer, Data and Mathematical Sciences, Western Sydney University, Sydney, New South Wales, Australia; Brain and Mind Centre, University of Sydney, Sydney, New South Wales, Australia; and Computer Simulation & Advanced Research Technologies, Sydney, New South Wales, Australia; Austin Hospital, Heidelberg, Victoria, Australia; Brain and Mind Centre, University of Sydney, Sydney, New South Wales, Australia; and St Vincent's Private Hospital, Sydney, New South Wales, Australia

**Keywords:** Emergency department, mental illness, youth, depression, data linkage

## Abstract

**Background:**

Primary youth mental health services in Australia have increased access to care for young people, yet the longer-term outcomes and utilisation of other health services among these populations is unclear.

**Aims:**

To describe the emergency department presentation patterns of a help-seeking youth mental health cohort.

**Method:**

Data linkage was performed to extract Emergency Department Data Collection registry data (i.e. emergency department presentations, pattern of re-presentations) for a transdiagnostic cohort of 7024 youths (aged 12–30 years) who presented to mental health services. Outcome measures were pattern of presentations and reason for presentations (i.e. mental illness; suicidal behaviours and self-harm; alcohol and substance use; accident and injury; physical illness; and other).

**Results:**

During the follow-up period, 5372 (76.5%) had at least one emergency department presentation. The presentation rate was lower for males (IRR = 0.87, 95% CI 0.86–0.89) and highest among those aged 18 to 24 (IRR = 1.117, 95% CI 1.086–1.148). Almost one-third (31.12%) had an emergency department presentation that was directly associated with mental illness or substance use, and the most common reasons for presentation were for physical illness and accident or injury. Index visits for mental illness or substance use were associated with a higher rate of re-presentation.

**Conclusions:**

Most young people presenting to primary mental health services also utilised emergency services. The preventable and repeated nature of many presentations suggests that reducing the ongoing secondary risks of mental disorders (i.e. substance misuse, suicidality, physical illness) could substantially improve the mental and physical health outcomes of young people.

Globally, mental disorders among young people are the leading cause of disability because of their impacts on overall health and productivity.^1^ Most mental disorders are also associated with substantial premature all-cause mortality due to the increased risk of developing subsequent medical conditions and increased risk of death by suicide.^2,3^ With over 75% of adult mental disorders emerging before the age of 25 years, these disorders often have lifelong impacts even if the disorder is subthreshold or has remitted, so effective mental healthcare during this period is critical to reduce the burden for individuals, society and the health system.^4–8^

The development and expansion of primary youth mental healthcare services in Australia has increased access to mental health services for young people.^9^ Previous work has demonstrated that young people accessing these services often present with attenuated mood, anxiety and psychotic syndromes,^10^ yet 14% reported a previous suicide attempt, which carries risks for worsening illness trajectories (e.g. onset of bipolar disorder, alcohol or substance misuse).^11^ Furthermore, two-thirds of young people accessing primary mental healthcare have poor social and occupational functional outcomes, which tend to be associated with significant physical health comorbidities and other indicators of disorder complexity.^12^ Concurrently we have seen that emergency department presentations for self-harm and suicidal behaviour in Australia are increasing,^13,14^ as are presentations for mental health diagnoses,^15^ with the greatest increase observed among young people. This may reflect increased help-seeking by young people, families and schools, but may also indicate that community mental health services are currently unable to meet the substantial demand and needs of young people.^16^ Although much research has been devoted to the increase in mental illness-related emergency presentations,^15^ few studies have investigated the patterns of emergency visits for young people in the mental health system. Indeed, young people with a mental disorder are more likely to utilise emergency services than those without,^17^ suggesting the need to understand the types of emergency department presentations common to young people in the mental health system.

Understanding the relationships between young people's use of primary mental healthcare and emergency services is crucial in developing a more complete understanding of their health service needs. Thus, the aims of this study were to describe the emergency department presentation patterns of a youth mental health cohort with emerging mood and psychotic syndromes, using a large, linked data-set of emergency department presentations in New South Wales (NSW), Australia.

## Method

### Study population

The study used data from the Brain and Mind Research Register (BPRR), a longitudinal cohort study of 7024 young people presenting to the youth mental health clinics of the Brain and Mind Centre in Sydney, Australia, between October 2008 and October 2020. Most young people initially presented to primary mental health services (i.e. ‘headspace’, which offers integrated mental health services for young people in Australia^18,19^), with some also going on to attend more specialised services.^10,20^ The service primarily attracts young people with a range of mental health problems, including those with subthreshold and full-threshold mental disorders. Young people may self-refer, or have been referred by family members, friends, school counselling services or via a general practitioner. All young people attending these clinics received clinician-based case management and psychological, social and/or medical interventions as part of standard care.

Inclusion criteria for this study were: (a) attendance of at least one visit at the Brain and Mind Centre's youth mental health services and (b) age between 12 and 30 years at first recorded presentation. Exclusion criteria for this study were: (a) medical instability or lack of capacity to give informed consent (as determined by a psychiatrist), (b) history of neurological disease (e.g. tumour, head trauma, epilepsy), (c) medical illness known to affect cognitive and brain function (e.g. cancer, electroconvulsive therapy in the past 3 months), (d) clinically evident intellectual disability and/or (e) insufficient English to participate in the research protocol.

### Patient and public involvement

Patients and carers were not directly involved in the initial study design. However, the clinical research team conducting this study is embedded within a clinical service and clinical research team with major lived experience involvement in all research activities, including the specific design of this study through regular consultation.

### Data and record linkage

The BPRR was linked to three external databases held by the Centre for Health Record Linkage in NSW, Australia. Complete details of the data linkage approach can be found in our previous work.^21,22^ Clinical research data for this cohort were linked to routinely collected data from the Emergency Department Data Collection (EDDC) registry over a 10-year period, from January 2010 to October 2020.^21^ A waiver of consent was granted according to the National Statement on Ethical Conduct in Human Research (2007). A master linkage key was generated for each participant to mask identifying information, including name, address, date of birth and gender. This key was then used by the NSW Centre for Health Record Linkage (CHeReL), which performed probabilistic matching by applying an automated blocking algorithm to identify records belonging to the same participant. The probabilistic matching was necessary to ensure accurate linkage of the data-sets while protecting the privacy of the participants. The false-positive record identification rate was controlled at 0.5%. Linkage was then performed using R statistical software (version 4.1.0) using the master linkage key to join across the EDDC registry, NSW Registry of Births, Deaths and Marriages (RBDM) and clinical research databases.

This study reports on data from the EDDC registry, which contains routinely collected data for presentations to all public hospital emergency departments in NSW (around 184 emergency departments in 2016−2017). Data analysed included arrival mode, type of visit, triage category, mode of separation (i.e. the manner in which the patient left the emergency department) and emergency department diagnosis (i.e. reason for presentation). SNOMED, ICD-9 and ICD-10 codes were used by two clinically trained authors to classify presentations into predetermined categories: (a) mental illness, (b) suicidal behaviours and self-harm, (c) alcohol and substance misuse, (d) accident and injury, (e) physical illness and (f) other. Interrater reliability was 98%, with all discrepancies between the coders resolved via consensus. A full list of emergency department codes, descriptions and associated presentation categories is given in the Supplementary materials available at https://dx.doi.org/10.1192/bjo.2023.521. The outcomes of interest for this study were the proportion of the cohort presenting to an emergency department, rate of emergency department presentation and re-presentation, total number of presentations per person, time between re-presentations and reason for presentations.

### Ethics statement

The clinical cohort study was approved by the University of Sydney's Human Research Committee (2008/5453 and 2012/1626) and the data linkage study was approved by NSW Population and Health Services Research Ethics Committee (2019/ETH12201).

### Statistical analyses

All statistical analyses and calculations were performed using R statistical software for Windows (version 4.1.0). Person-years of follow-up were calculated as the time (in years) between 1 January 2010 and 30 September 2020 that participants were aged 12 years or older (or the relevant age range for age-specific rates). Emergency department presentations that occurred when participants were younger than 12 years were excluded. Those who had a date of death recorded during the study (*n* = 58) were censored at that date and the corresponding emergency department presentation associated with death (*n* = 33) was also excluded. The absolute number of emergency department presentations and time between consecutive presentations were calculated for each participant. Group differences for categorical variables were compared using chi-squared tests of independence, crude incidence of emergency department presentations per 1000 person-years and unadjusted incidence rate ratios (IRR) using the ‘rateratio.test’ package,^23^ which conducts an unbiased test of Poisson incidence ratios given person-years of follow-up. Nominal values for emergency department presentations in NSW by gender, age and reason during 2018–2019 were also reported where appropriate and compared by percentage and incidence rate per 1000 person-years (which is equivalent to the yearly rate per 1000 population).

## Results

**Fig. 1:**
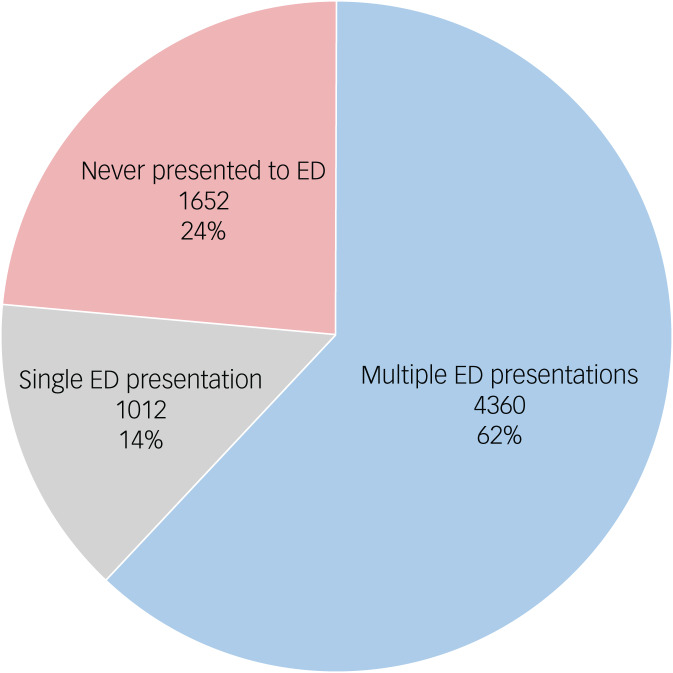
Emergency department (ED) presentation status among a youth mental health cohort (*n* = 7024).

**Fig. 2:**
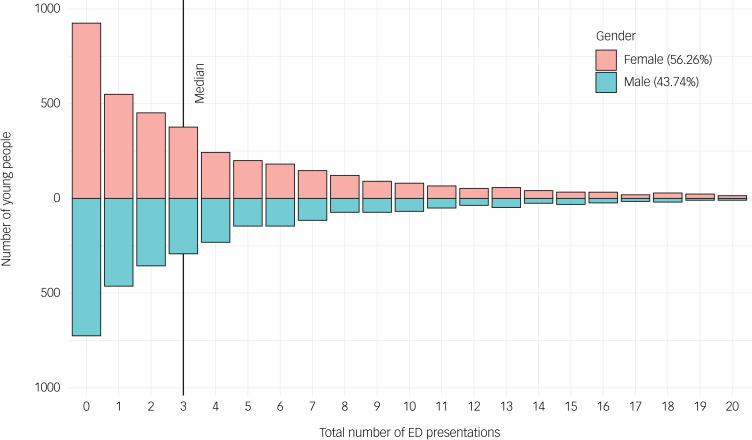
Distribution of total number of emergency department (ED) presentations over the course of follow-up for each individual by gender.

Of the 7024 individuals from this cohort, three-quarters (5372/7024; 76.5%) had at least one emergency department presentation during the follow-up period (i.e. January 2010 to October 2020) (Fig. 1). A higher proportion of the total cohort had multiple presentations (4360/7024; 62.1%) compared with a single presentation (1012/7024; 14.4%). There were 39 155 emergency department presentations across the cohort, with a median of 3 visits per person (IQR = 1–6) (Fig. 2). Despite there being a slightly greater proportion of females (3952/7024; 56.3%) than males, gender did not account for a statistical difference in the likelihood of individuals presenting to an emergency department at least once during the study follow-up (χ^2^(1) = 0.039, *P* = 0.843). Yet, among those who did present, the presentation rate was lower for males (IRR = 0.87, 95% CI 0.86–0.89) than for females and was higher for those aged between 18 and 24 years (IRR = 1.12, 95% CI 1.09–1.15) than for other age groups. Rates of presentation were up to three times higher than the NSW state-wide reported incidence for emergency department presentations (Table 1).

**Table 1:** Crude incidence for all presentations to an emergency department in the full cohort (*n* = 7024)

Variable	Individuals presenting, *n*	Person-years of follow-up	Emergency department visits, *n* (%)	Crude incidence (per 1000 person-years)	IRR (95% CI)	Incidence in NSW (per 1000 population)^a^
Gender						
Female	3952	41 865.62	23 352 (59.6%)	557.78	ref	333.71
Male	3072	32 490.3	15 803 (40.4%)	486.39	0.872 (0.855–0.890)	329.65
Age, years^b^						
12–17	3727	12 396.56	6561 (16.3%)	529.26	ref	287.66
18–24	6233	33 270.58	19 663 (48.7%)	591.00	1.117 (1.086–1.148)	260.28
25–34	5398	28 688.78	14 120 (35.0%)	492.18	0.930 (0.903–0.958)	287.66

IRR, incidence rate ratio; NSW, New South Wales; ref, reference.

a. NSW rate sourced from the Australian Institute of Health and Welfare 2018–19 report on Mental Health Services in Emergency Departments.^24^

b. Counts in each category are cumulative and may include individuals contributing to multiple age groups over the 10-year follow-up period.

Of the total cohort, 2186/7024 (31.12%) young people had an emergency department presentation that was directly associated with mental illness or substance use (i.e. mental illness, suicidal behaviours and self-harm, alcohol and substance misuse). The highest rates of mental illness presentations were among those aged 18–24 years (IRR = 1.10, 95% CI 1.013–1.197) and 25–34 years (IRR = 1.09, 95% CI 1.001–1.187) when compared with ages 12–17 years. These rates were between three and five times higher than the NSW state-wide reported incidence for mental illness emergency department presentations (Table 2).

**Table 2:** Crude incidence for mental illness emergency department presentations in the full cohort (*n* = 7024)

Variable	Mental illness emergency department visits, *n*	Crude incidence (per 1000 person-years)	IRR (95% CI)	Incidence in NSW (per 1000 population)^a^
Gender				
Female	2888	68.98	ref	12.71
Male	2238	68.88	1.00 (0.944–1.056)	11.39
Age, years^b^				
12–17	753	60.74	ref	14.83
18–24	2224	66.85	1.10 (1.013–1.197)	20.51
25–34	1899	66.19	1.09 (1.001–1.187)	16.63

IRR, incidence rate ratio; NSW, New South Wales; ref, reference.

a. NSW rate sourced from the Australian Institute of Health and Welfare 2018–19 report on Mental Health Services in Emergency Departments.^24^

b. Counts in each category are cumulative and may include individuals contributing to multiple age groups over the 10-year follow-up period.

The most common reason for presentation was for physical illness, accounting for over half of all emergency department presentations (19 103/37 001; 51.6%), followed by accident and injury (7094/37 001; 19.2%), mental illness (5126/37 001; 13.9%), suicidal behaviours and self-harm (2370/37 001; 6.4%), alcohol and substance misuse (1959/37 001; 5.3%) and other (1349/37 001; 3.6%). Mental illness or substance use (i.e. mental illness, suicidal behaviours and self-harm, alcohol and substance misuse) presentations were more likely to be triaged as emergency or urgent presentations compared with physical illness, accident and injury and other presentations (χ^2^(1) = 1565.3, *P* < 0.001).

There were 4360/7024 (62.1%) young people who had multiple emergency department presentations, accounting for 33 783/37 001 (91.3%) of the total number of presentations. Almost one-third of these occurred within 28 days of a previous presentation (10 825/33 783; 32.0%), 6233/33 783 (18.5%) occurred between 28 days and 3 months of a previous presentation, 9140/33 783 (27.1%) occurred between 3 and 12 months of a previous presentation and 7585/33 783 (22.5%) occurred more than 1 year after a previous presentation. Index visits (i.e. first visits to an emergency department) that were for mental illness or substance use (i.e. mental illness, suicidal behaviours and self-harm, alcohol and substance misuse) were associated with a higher rate of re-presentation, compared with physical illness, accident and injury and other presentations (IRR = 1.20, 95% CI 1.17–1.23).

**Fig. 3:**
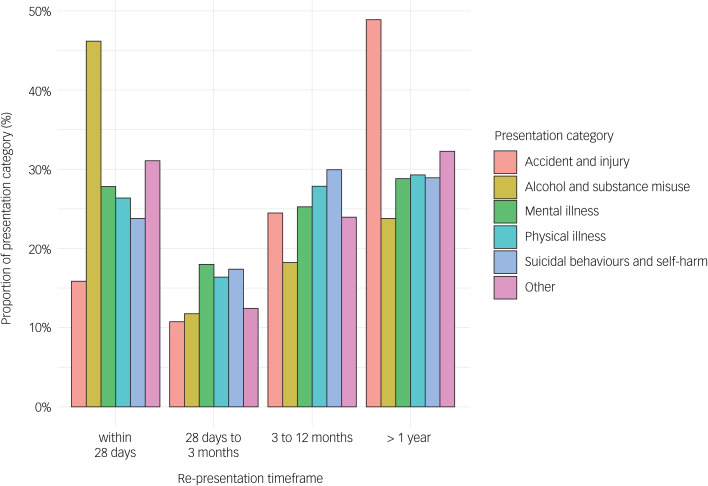
The time course for re-presentations and reason for re-presentation.

Of the total number of repeat emergency department presentations, 74.0% (25 011/33 783) were defined as re-presentations (i.e. person re-presenting for the same reason during the study period). A quarter of re-presentations occurred within 28 days of a previous presentation (6448/25 011; 25.8%), 3865/25 011 (15.5%) occurred between 28 days and 3 months of a previous presentation, 6655/25 011 (26.6%) occurred between 3 and 12 months of a previous presentation and 8043/25 011 (32.2%) occurred more than 1 year after a previous presentation (Fig. 3).

**Fig. 4:**
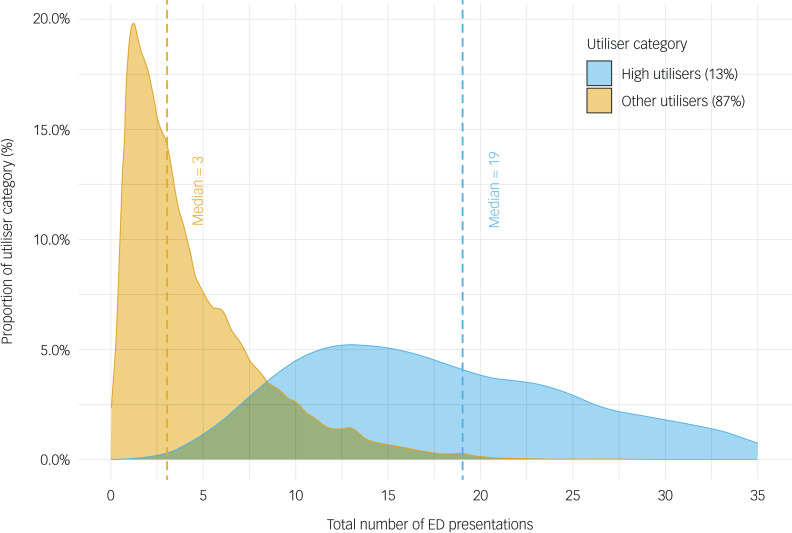
Distributions of emergency department (ED) presentations for high utilisers (more than four presentations within a 12-month period) versus other utilisers.

In total, 9.9% (696/7024) of the cohort were identified as ‘high utilisers’ (i.e. more than four presentations within a 12-month period), and 13.1% (696/5372) of those who presented to an emergency department were high utilisers (Fig. 4). High utilisers were more likely to be female (429/696; 61.6%) (χ^2^(1) = 8.180, *P* < 0.01) with a median of 20 presentations (IQR = 14–31) over the entire follow-up period, whereas males (267/696, 38.4%) had a median of 18 presentations (IQR = 15, 95% CI 12–27).

## Discussion

This study shows that three out of four young people aged between 12 and 30 years who engaged with youth mental health services also utilised emergency services for mental or physical health problems. Multiple presentations were more common than one-off presentations, which may suggest that persistent or recurring health problems were not being sufficiently addressed by primary youth mental healthcare services. Almost one-third of the cohort presented to an emergency department for mental illness or substance use (i.e. mental illness, self-harm and suicide, alcohol and substance related), although mental illness or substance use only accounted for a quarter of all emergency department presentations. The most common reasons for presentation were physical illness and accident or injury (accounting for ~70%), which may reflect the broader risks associated with mental illness among young people engaging with primary care services.

### Implications

This study has important implications for primary youth mental healthcare as it highlights the broad and heightened risks among these young people. The high rate and repeated use of emergency services suggest that current primary care-based services are not fully meeting the needs of young people. This supports calls for enhancing the primary care model with better integration of multidisciplinary and secondary care so that young people get the right type of care when they first present.^25^ A large proportion of young people are at risk for persistent and recurring mental health problems and secondary impacts that require intensive and ongoing care.^26^ This includes a substantial proportion of the total cohort (9.9%) who were identified as high utilisers of emergency services, with a median of 19 visits during the follow-up period. Yet, the current system does not appear to have the capacity or capability to provide the type of personalised treatment that allocates care based on prior increased risk for progression and extension of illness.^27^ Identifying these young people and facilitating their entry into the right level of care is critical and the role of digital technologies to facilitate this should be examined.^28^

The re-presentation rate for this cohort was high, with 25.8% occurring within 28 days of a previous emergency department presentation and a further 15.5% occurring within 3 months. This finding supports prior research that established a positive association between mood disorders and recurring utilisation of emergency department services.^29^ We also found that re-presentations were more common among those with mental health or substance use emergency department presentations (i.e. mental illness, suicidal behaviours or self-harm, alcohol and substance use), which is consistent with previous findings.^30^ These presentations are typically indicative of inadequate emergency department or community-based care and associated with substantially higher healthcare costs.^31^ This reiterates the lack of sufficient effective provision of, or engagement with, specific evidence-based interventions, such as assertive follow-up and aftercare, that directly reduce the rate of re-presentation.^32,33^ Such interventions can have major impacts on population-level rates of self-harm and suicide if taken to scale,^34^ yet the implementation of, and resources allocated to, these interventions vary across the healthcare system.

Higher rates of health service use, even for non-mental health reasons, in the months prior to suicide are common and provide an opportunity to engage in effective intervention and secondary prevention.^35^ Although the reason for the relationship between mental illness and emergency department presentations due to physical illness or accident and injury is not clear, these findings support the view that improving multidisciplinary and coordinated care for young people is critical.^25,28^ We know that young people who experience mental illness are more likely to develop physical illnesses, either as a direct result of mental illness and its treatment (e.g. antipsychotic medication) or due to shared genetic risk factors or other environmental factors (e.g. socioeconomic, substance use).^36^ Most of these factors are associated with both increased risk of suicide and contribute to increased mortality from other causes.^37^ Thus, this work emphasises the need for greater intervention to not only reduce mortality due to suicide, but for other causes of death related to physical illnesses.^38^

There are also major secondary prevention implications given that this cohort has an increased risk of developing poorer health outcomes that reach the threshold of requiring emergency care. The elevated risk of suicidal behaviours and self-harm in these populations of young people justifies broad health service-based suicide prevention strategies, such as safety planning (a documented plan for the management of risk-related behaviours should they arise), even among those without a history of such behaviours.^39^ Further, many secondary interventions could also target factors that typically emerge during adolescence and young adulthood and contribute to an increased burden of illness and poor illness trajectories, such as alcohol and substance misuse, which accounted for over 5% of all emergency department presentations.^40^ Critically, re-presentations for alcohol and substance misuse were most likely to occur within 28 days of a previous presentation, indicating the lack of adequate high-quality prevention and treatment among those engaged with the health system.

This study provides valuable insights into the broader patterns of health service use for a cohort of young people engaged in primary mental healthcare. The findings indicate that most young people presenting to these services experience poor mental and physical health outcomes which result in presentations to emergency departments. The preventable nature of many presentations suggests that improving the provision of multidisciplinary and coordinated ambulatory and non-urgent care to focus on reducing many of the primary and secondary risks associated with mental disorders, such as alcohol and substance misuse, suicidal thoughts and behaviours and physical illness, could substantially improve the mental and physical health outcomes of young people.

### Strengths and limitations

This study utilises a large transdiagnostic youth mental health cohort and data linkage methods to provide valuable insights into the wider health service use patterns of these young people over a 10-year period. Reporting of all types of emergency department presentations (mental illness; suicidal behaviours and self-harm; alcohol and substance use; accident and injury; physical illness; and other) details the full breadth of health service needs for young people presenting for youth mental healthcare. Follow-up and linkage was systematic and included all individuals in the youth mental health cohort.

Given that our study is based on administrative data entered by clinicians, the reliability of emergency department diagnoses (i.e. reason for presentation) is unknown and should be interpreted with caution. We used the broad diagnostic classifications to improve the validity of these emergency department diagnoses, since we assume that the reliability of these broad categories would be greater than the within-group classifications. We did not collect information about the impact of other sociodemographic or clinical variables (e.g. age at onset, diagnosis), and future work should focus on trying to understand the role these other factors play in the frequency and timing of emergency department presentations. We also did not adjust for demographic variables such as age or gender in our statistical models, which could potentially affect our findings. Additionally, the time course of emergency department presentations and contacts with primary care-based youth mental health services was not assessed, so it is unclear how actively engaged an individual was with primary services at the time of an emergency department presentation. A more detailed analysis of this would provide further insights into whether emergency department presentations were commonly an entry point into care, the frequency of these visits prior to contact with other services and whether they occurred during episodes of active care. Understanding these pathways is critical to understanding whether prevention efforts are adequate at engaging young people who have never presented to services earlier in the course of illness and whether improved monitoring of those in remission or in active care is needed.

## Supporting information

Iorfino et al. supplementary material Iorfino et al. supplementary material

## Data Availability

The data presented in this manuscript are available from the corresponding author on request.
