# Supplementary material for: Patterns of emergency department presentations for a youth mental health cohort: data-linkage cohort study
Source: BJPsych Open. 2023 Sep 14;9(5):e170. doi: 10.1192/bjo.2023.521 (PMC10594097; doi:10.1192/bjo.2023.521)
Supplement: Iorfino et al. supplementary material [file S2056472423005215sup001.pdf]

Supplementary Material

Supplement 1. Full list of reasons for presentation to emergency departments with classification and ICD-10 or SNOMED codes.

ed_code	description	presentation_category	classification
005.9	Food poisoning, unspecified	Physical illness	icd
008.8	Intestinal infection due to other organism, not elsewhere classified	Physical illness	icd
009.0	Infectious colitis, enteritis, and gastroenteritis	Physical illness	icd
009.1	Colitis, enteritis, and gastroenteritis of presumed infectious origin	Physical illness	icd
034.1	Scarlet fever	Physical illness	icd
054.11	Herpetic vulvovaginitis	Physical illness	icd
075	Infectious mononucleosis	Physical illness	icd
079.9	Adenovirus infection in conditions classified elsewhere and of unspecified site	Physical illness	icd
133.0	Scabies	Physical illness	icd
276.5	Hyperosmolality and/or hypernatremia	Physical illness	icd
279.8	Other specified disorders involving the immune mechanism	Physical illness	icd
292.0	Drug withdrawal	Alcohol and substance misuse	icd
292.9	Unspecified drug−induced mental disorder	Mental health	icd
295.30	Paranoid type schizophrenia, unspecified	Accident and injury	icd
295.90	Unspecified schizophrenia, unspecified	Mental health	icd
296.0	Bipolar I disorder, single manic episode, unspecified	Mental health	icd
296.20	Major depressive affective disorder, single episode, unspecified	Mental health	icd
296.40	Bipolar I disorder, most recent episode (or current) manic, unspecified	Mental health	icd
296.50	Bipolar I disorder, most recent episode (or current) depressed, unspecified	Mental health	icd
296.7	Bipolar I disorder, most recent episode (or current) unspecified	Mental health	icd
298.9	Unspecified psychosis	Mental health	icd
300.00	Anxiety state, unspecified	Mental health	icd
300.01	Panic disorder without agoraphobia	Mental health	icd
300.11	Conversion disorder	Mental health	icd
300.4	Dysthymic disorder	Mental health	icd
300.9	Unspecified nonpsychotic mental disorder	Mental health	icd
301.9	Unspecified personality disorder	Mental health	icd
303.00	Acute alcoholic intoxication in alcoholism, unspecified	Alcohol and substance misuse	icd
303.90	Other and unspecified alcohol dependence, unspecified	Alcohol and substance misuse	icd
305.00	Alcohol abuse, unspecified	Alcohol and substance misuse	icd
306.9	Unspecified psychophysiological malfunction	Mental health	icd
307.1	Anorexia nervosa	Mental health	icd
307.51	Bulimia nervosa	Mental health	icd
307.9	Other and unspecified special symptoms or syndromes, not elsewhere classified	Other	icd
308.0	Predominant disturbance of emotions	Mental health	icd

ed_code	description	presentation_category	classification
308.9	Unspecified acute reaction to stress	Mental health	icd
309.24	Adjustment disorder with anxiety	Mental health	icd
309.9	Unspecified adjustment reaction	Mental health	icd
311	Depressive disorder, not elsewhere classified	Mental health	icd
312.90	Undersocialized conduct disorder, aggressive type, unspecified	Mental health	icd
313.9	Unspecified emotional disturbance of childhood or adolescence	Mental health	icd
340	Multiple sclerosis	Physical illness	icd
345.10	Generalized convulsive epilepsy, without mention of intractable epilepsy	Physical illness	icd
345.3	Grand mal status	Physical illness	icd
345.90	Epilepsy, unspecified, without mention of intractable epilepsy	Physical illness	icd
346.90	Migraine, unspecified, without mention of intractable migraine without mention of status migrainosus	Physical illness	icd
348.5	Cerebral edema	Physical illness	icd
368.16	Psychophysical visual disturbances	Mental health	icd
370.9	Unspecified keratitis	Physical illness	icd
376.01	Orbital cellulitis	Physical illness	icd
379.91	Pain in or around eye	Physical illness	icd
379.92	Swelling or mass of eye	Physical illness	icd
379.93	Redness or discharge of eye	Physical illness	icd
380.10	Infective otitis externa, unspecified	Physical illness	icd
382.9	Unspecified otitis media	Physical illness	icd
388.70	Otalgia, unspecified	Physical illness	icd
415.1	Acute cor pulmonale	Physical illness	icd
423.9	Unspecified disease of pericardium	Physical illness	icd
435.8	Other specified transient cerebral ischemias	Physical illness	icd
458.9	Hypotension, unspecified	Physical illness	icd
460	Acute nasopharyngitis [common cold]	Physical illness	icd
461.0	Acute maxillary sinusitis	Physical illness	icd
462	Acute pharyngitis	Physical illness	icd
463	Acute tonsillitis	Physical illness	icd
465.9	Acute upper respiratory infections of unspecified site	Physical illness	icd
478.1	Hypertrophy of nasal turbinates	Physical illness	icd
482.9	Bacterial pneumonia, unspecified	Physical illness	icd
487.1	Influenza with other respiratory manifestations	Physical illness	icd
493.90	Asthma, unspecified type, unspecified	Physical illness	icd
519.8	Other diseases of respiratory system, not elsewhere classified	Physical illness	icd

ed_code	description	presentation_category	classification
525.9	Unspecified disorder of the teeth and supporting structures	Physical illness	icd
526.9	Unspecified disease of the jaws	Physical illness	icd
530.1	Achalasia and cardiospasm	Physical illness	icd
535.30	Alcoholic gastritis, without mention of hemorrhage	Alcohol and substance misuse	icd
535.50	Unspecified gastritis and gastroduodenitis, without mention of hemorrhage	Physical illness	icd
536.2	Persistent vomiting	Physical illness	icd
540.9	Acute appendicitis without mention of peritonitis	Physical illness	icd
556	Ulcerative (chronic) enterocolitis	Physical illness	icd
558.9	Other and unspecified noninfectious gastroenteritis and colitis	Physical illness	icd
564.0	Constipation, unspecified	Physical illness	icd
565.0	Anal fissure	Physical illness	icd
566	Abscess of anal and rectal regions	Physical illness	icd
569.42	Anal or rectal pain	Physical illness	icd
573.9	Unspecified disorder of liver	Physical illness	icd
578.9	Hemorrhage of gastrointestinal tract, unspecified	Physical illness	icd
580.9	Acute glomerulonephritis with unspecified pathological lesion in kidney	Physical illness	icd
581.9	Nephrotic syndrome with unspecified pathological lesion in kidney	Physical illness	icd
590.10	Acute pyelonephritis without lesion of renal medullary necrosis	Physical illness	icd
590.80	Pyelonephritis, unspecified	Physical illness	icd
592.1	Calculus of ureter	Physical illness	icd
599.0	Urinary tract infection, site not specified	Physical illness	icd
614.9	Unspecified inflammatory disease of female pelvic organs and tissues	Physical illness	icd
616.3	Abscess of Bartholin's gland	Physical illness	icd
626.4	Irregular menstrual cycle	Physical illness	icd
626.9	Unspecified disorders of menstruation and other abnormal bleeding from female genital tract	Physical illness	icd
681.10	Cellulitis and abscess of toe, unspecified	Physical illness	icd
682.3	Cellulitis and abscess of upper arm and forearm	Physical illness	icd
682.6	Cellulitis and abscess of leg, except foot	Physical illness	icd
683	Acute lymphadenitis	Physical illness	icd
685.0	Pilonidal cyst with abscess	Physical illness	icd
692.71	Sunburn	Accident and injury	icd
693.0	Dermatitis due to drugs and medicines taken internally	Physical illness	icd
703.0	Ingrowing nail	Physical illness	icd
704.8	Other specified diseases of hair and hair follicles	Physical illness	icd
708.9	Urticaria, unspecified	Physical illness	icd

ed_code	description	presentation_category	classification
719.04	Effusion of joint, hand	Physical illness	icd
719.41	Pain in joint, shoulder region	Physical illness	icd
719.42	Pain in joint, upper arm	Physical illness	icd
719.44	Pain in joint, hand	Physical illness	icd
719.45	Pain in joint, pelvic region and thigh	Physical illness	icd
719.46	Pain in joint, lower leg	Physical illness	icd
719.47	Pain in joint, ankle and foot	Physical illness	icd
722.10	Displacement of lumbar intervertebral disc without myelopathy	Accident and injury	icd
722.73	Intervertebral disc disorder with myelopathy, lumbar region	Physical illness	icd
723.1	Cervicalgia	Physical illness	icd
723.5	Torticollis, unspecified	Physical illness	icd
724.2	Lumbago	Physical illness	icd
724.5	Backache, unspecified	Physical illness	icd
726.33	Olecranon bursitis	Accident and injury	icd
733.99	Other disorders of bone and cartilage	Physical illness	icd
742.3	Congenital hydrocephalus	Physical illness	icd
754.2	Congenital musculoskeletal deformities of spine	Physical illness	icd
780.09	Other alteration of consciousness	Physical illness	icd
780.1	Hallucinations	Mental health	icd
780.2	Syncope and collapse	Physical illness	icd
780.3	Coma	Physical illness	icd
780.4	Dizziness and giddiness	Physical illness	icd
780.50	Sleep disturbance, unspecified	Physical illness	icd
780.52	Insomnia, unspecified	Physical illness	icd
780.6	Coma	Physical illness	icd
780.7	Coma	Physical illness	icd
781.0	Abnormal involuntary movements	Physical illness	icd
782.0	Disturbance of skin sensation	Physical illness	icd
782.2	Localized superficial swelling, mass, or lump	Physical illness	icd
783.0	Anorexia	Physical illness	icd
784.0	Headache	Physical illness	icd
784.1	Throat pain	Physical illness	icd
784.2	Swelling, mass, or lump in head and neck	Physical illness	icd
785.1	Palpitations	Physical illness	icd
786.09	Other respiratory abnormalities	Physical illness	icd

ed_code	description	presentation_category	classification
786.2	Cough	Physical illness	icd
786.3	Respiratory abnormality, unspecified	Physical illness	icd
786.50	Chest pain, unspecified	Physical illness	icd
786.52	Painful respiration	Physical illness	icd
786.59	Other chest pain	Physical illness	icd
787.0	Nausea with vomiting	Physical illness	icd
789.0	Abdominal pain, unspecified site	Physical illness	icd
789.3	Abdominal pain, unspecified site	Physical illness	icd
790.6	Other abnormal blood chemistry	Physical illness	icd
799.1	Respiratory arrest	Physical illness	icd
799.2	Asphyxia	Accident and injury	icd
799.9	Other unknown and unspecified cause of morbidity and mortality	Physical illness	icd
802.2	Closed fracture of nasal bones	Accident and injury	icd
802.4	Closed fracture of malar and maxillary bones	Accident and injury	icd
802.6	Closed fracture of orbital floor (blow−out)	Accident and injury	icd
807.0	Closed fracture of rib(s), unspecified	Accident and injury	icd
810.0	Fracture of clavicle	Accident and injury	icd
813.0	Closed fracture of upper end of forearm, unspecified	Accident and injury	icd
813.4	Closed fracture of upper end of forearm, unspecified	Accident and injury	icd
814.0	Closed fracture of carpal bone, unspecified	Accident and injury	icd
815.0	Closed fracture of metacarpal bone(s), site unspecified	Accident and injury	icd
816.0	Closed fracture of phalanx or phalanges of hand, unspecified	Accident and injury	icd
822.1	Open fracture of patella	Accident and injury	icd
823.8	Closed fracture of upper end of tibia alone	Accident and injury	icd
824.2	Fracture of lateral malleolus, closed	Accident and injury	icd
825.2	Fracture of calcaneus, closed	Accident and injury	icd
826.0	Closed fracture of one or more phalanges of foot	Accident and injury	icd
831.00	Closed dislocation of shoulder, unspecified	Accident and injury	icd
834.00	Closed dislocation of finger, unspecified part	Accident and injury	icd
836.3	Dislocation of patella, closed	Accident and injury	icd
840.4	Rotator cuff (capsule) sprain	Accident and injury	icd
840.9	Sprains and strains of unspecified site of shoulder and upper arm	Accident and injury	icd
841.9	Sprains and strains of unspecified site of elbow and forearm	Accident and injury	icd
842.10	Sprain of hand, unspecified site	Accident and injury	icd
844.9	Sprains and strains of unspecified site of knee and leg	Accident and injury	icd

ed_code	description	presentation_category	classification
845.0	Sprain of ankle, unspecified site	Accident and injury	icd
845.00	Sprain of ankle, unspecified site	Accident and injury	icd
845.10	Sprain of foot, unspecified site	Accident and injury	icd
845.13	Sprain of interphalangeal (joint), toe	Accident and injury	icd
847.0	Sprain of neck	Accident and injury	icd
847.2	Sprain of lumbar	Accident and injury	icd
847.4	Sprain of coccyx	Accident and injury	icd
850.1	Concussion with no loss of consciousness	Accident and injury	icd
850.2	Concussion with moderate loss of consciousness	Accident and injury	icd
850.9	Concussion, unspecified	Accident and injury	icd
854.01	Intracranial injury of other and unspecified nature without mention of open intracranial wound, with no loss of consciousness	Accident and injury	icd
854.02	Intracranial injury of other and unspecified nature without mention of open intracranial wound, with brief [less than one hour] loss of consciousness	Accident and injury	icd
854.06	Intracranial injury of other and unspecified nature without mention of open intracranial wound, with loss of consciousness of unspecified duration	Accident and injury	icd
854.09	Intracranial injury of other and unspecified nature without mention of open intracranial wound, with concussion, unspecified	Accident and injury	icd
873.0	Open wound of scalp, without mention of complication	Accident and injury	icd
873.4	Open wound of scalp, without mention of complication	Accident and injury	icd
873.6	Open wound of scalp, without mention of complication	Accident and injury	icd
873.63	Open wound of tooth (broken) (fractured) (due to trauma), without mention of complication	Accident and injury	icd
873.8	Other and unspecified open wound of head without mention of complication	Accident and injury	icd
876.0	Open wound of back, without mention of complication	Accident and injury	icd
878.2	Open wound of scrotum and testes, without mention of complication	Accident and injury	icd
881.0	Open wound of forearm, without mention of complication	Accident and injury	icd
882.0	Open wound of hand except finger(s) alone, without mention of complication	Accident and injury	icd
883.0	Open wound of finger(s), without mention of complication	Accident and injury	icd
890.0	Open wound of hip and thigh, without mention of complication	Accident and injury	icd
891.0	Open wound of knee, leg [except thigh], and ankle, without mention of complication	Accident and injury	icd
893.0	Open wound of toe(s), without mention of complication	Accident and injury	icd
914.6	Superficial foreign body (splinter) of hand(s) except finger(s) alone, without major open wound and without mention of infection	Accident and injury	icd
914.8	Other and unspecified superficial injury of hand(s) except finger(s) alone, without mention of infection	Accident and injury	icd
915.6	Superficial foreign body (splinter) of finger(s), without major open wound and without mention of infection	Accident and injury	icd
917.5	Insect bite, nonvenomous, of foot and toe(s), infected	Accident and injury	icd
918.1	Superficial injury of cornea	Accident and injury	icd
919.4	Insect bite, nonvenomous, of other, multiple, and unspecified sites, without mention of infection	Accident and injury	icd
920	Contusion of face, scalp, and neck except eye(s)	Accident and injury	icd
921.9	Unspecified contusion of eye	Accident and injury	icd

ed_code	description	presentation_category	classification
922.2	Contusion of abdominal wall	Accident and injury	icd
923.3	Contusion of finger	Accident and injury	icd
923.9	Contusion of unspecified part of upper limb	Accident and injury	icd
924.0	Contusion of thigh	Accident and injury	icd
924.1	Contusion of thigh	Accident and injury	icd
924.2	Contusion of thigh	Accident and injury	icd
930.0	Corneal foreign body	Accident and injury	icd
930.9	Foreign body in unspecified site on external eye	Accident and injury	icd
931	Foreign body in ear	Accident and injury	icd
934.9	Foreign body in respiratory tree, unspecified	Accident and injury	icd
935.0	Foreign body in mouth	Accident and injury	icd
935.1	Foreign body in esophagus	Accident and injury	icd
935.2	Foreign body in stomach	Accident and injury	icd
943.2	Burn of unspecified degree of upper limb, except wrist and hand, unspecified site	Accident and injury	icd
944.2	Burn of unspecified degree of hand, unspecified site	Accident and injury	icd
945.1	Burn of unspecified degree of lower limb [leg], unspecified site	Accident and injury	icd
946.1	Erythema [first degree] of multiple specified sites	Accident and injury	icd
956.3	Injury to peroneal nerve	Accident and injury	icd
957.7	Injury to superficial nerves of head and neck	Accident and injury	icd
959.1	Head injury, unspecified	Accident and injury	icd
959.2	Shoulder and upper arm injury	Accident and injury	icd
959.4	Hand, except finger injury	Accident and injury	icd
962.9	Poisoning by other and unspecified hormones and synthetic substitutes	Alcohol and substance misuse	icd
963.0	Poisoning by antiallergic and antiemetic drugs	Suicidal behaviours and self−harm	icd
965.00	Poisoning by opium (alkaloids), unspecified	Alcohol and substance misuse	icd
965.01	Poisoning by heroin	Alcohol and substance misuse	icd
965.1	Poisoning by salicylates	Suicidal behaviours and self−harm	icd
965.4	Poisoning by aromatic analgesics, not elsewhere classified	Suicidal behaviours and self−harm	icd
965.6	Poisoning by opium (alkaloids), unspecified	Alcohol and substance misuse	icd
968.5	Surface (topical) and infiltration anesthetics	Physical illness	icd
969.0	Poisoning by antidepressant, unspecified	Suicidal behaviours and self−harm	icd
969.4	Poisoning by benzodiazepine−based tranquilizers	Alcohol and substance misuse	icd
969.6	Poisoning by psychodysleptics (hallucinogens)	Alcohol and substance misuse	icd
969.8	Poisoning by other specified psychotropic agents	Suicidal behaviours and self−harm	icd
970.8	Poisoning by analeptics	Suicidal behaviours and self−harm	icd

ed_code	description	presentation_category	classification
972.9	Poisoning by other and unspecified agents primarily affecting the cardiovascular system	Physical illness	icd
977.9	Poisoning by unspecified drug or medicinal substance	Suicidal behaviours and self−harm	icd
987.8	Toxic effect of other specified gases, fumes, or vapors	Alcohol and substance misuse	icd
995.2	Other anaphylactic reaction	Physical illness	icd
995.3	Allergy, unspecified, not elsewhere classified	Physical illness	icd
995.81	Adult physical abuse	Accident and injury	icd
998.1	Postoperative shock, unspecified	Physical illness	icd
999.5	Generalized vaccinia as a complication of medical care, not elsewhere classified	Physical illness	icd
A03.9	Shigellosis, unspecified	Physical illness	icd
A05.9	Bacterial foodborne intoxication, unspecified	Physical illness	icd
A08.5	Rotaviral enteritis	Physical illness	icd
A085	Rotaviral enteritis	Physical illness	icd
A09	Infectious gastroenteritis and colitis, unspecified	Physical illness	icd
A09.0	Infectious gastroenteritis and colitis, unspecified	Physical illness	icd
A09.9	Infectious gastroenteritis and colitis, unspecified	Physical illness	icd
A37.9	Whooping cough due to Bordetella pertussis without pneumonia	Physical illness	icd
A49.9	Bacterial infection, unspecified	Physical illness	icd
A54.0	Gonococcal infection of lower genitourinary tract, unspecified	Physical illness	icd
A60.0	Herpesviral infection of urogenital system, unspecified	Physical illness	icd
A64	Unspecified sexually transmitted disease	Physical illness	icd
B01.9	Varicella without complication	Physical illness	icd
B07	Plantar wart	Physical illness	icd
B09	Unspecified viral infection characterized by skin and mucous membrane lesions	Physical illness	icd
B34.9	Viral infection, unspecified	Physical illness	icd
B349	Adenovirus infection, unspecified	Physical illness	icd
B37.9	Candidiasis, unspecified	Physical illness	icd
B86	Scabies	Physical illness	icd
B97.8	Adenovirus as the cause of diseases classified elsewhere	Physical illness	icd
D50.9	Iron deficiency anemia, unspecified	Physical illness	icd
E03.9	Unspecified activity	Other	icd
E10.11	Type 1 diabetes mellitus with ketoacidosis with coma	Physical illness	icd
E10.12	Type 1 diabetes mellitus with ketoacidosis without coma	Physical illness	icd
E10.8	Type 1 diabetes mellitus with unspecified complications	Physical illness	icd
E11.02	Type 2 diabetes mellitus with hyperosmolarity without nonketotic hyperglycemic−hyperosmolar coma (NKHHC)	Physical illness	icd
E11.11	Type 2 diabetes mellitus with ketoacidosis with coma	Physical illness	icd

ed_code	description	presentation_category	classification
E16.2	Hypoglycemia, unspecified	Physical illness	icd
E27.4	Other adrenocortical overactivity	Physical illness	icd
E29.9	Testicular dysfunction, unspecified	Physical illness	icd
E86	Accidental poisoning by alcoholic beverages	Alcohol and substance misuse	icd
E920.4	Accidents caused by powered lawn mower	Accident and injury	icd
F06.9	Psychotic disorder with hallucinations due to known physiological condition	Mental health	icd
F09	Unspecified mental disorder due to known physiological condition	Mental health	icd
F10.0	Alcohol abuse, uncomplicated	Alcohol and substance misuse	icd
F10.2	Alcohol abuse, uncomplicated	Alcohol and substance misuse	icd
F10.5	Alcohol abuse, uncomplicated	Alcohol and substance misuse	icd
F10.9	Alcohol abuse, uncomplicated	Alcohol and substance misuse	icd
F100	Alcohol abuse, uncomplicated	Alcohol and substance misuse	icd
F102	Alcohol abuse, uncomplicated	Alcohol and substance misuse	icd
F11.2	Opioid abuse, uncomplicated	Alcohol and substance misuse	icd
F129	Cannabis abuse, uncomplicated	Alcohol and substance misuse	icd
F15.20	Other stimulant dependence, uncomplicated	Alcohol and substance misuse	icd
F19.2	Other psychoactive substance abuse, uncomplicated	Alcohol and substance misuse	icd
F19.3	Other psychoactive substance abuse, uncomplicated	Alcohol and substance misuse	icd
F19.9	Other psychoactive substance abuse, uncomplicated	Alcohol and substance misuse	icd
F192	Other psychoactive substance abuse, uncomplicated	Alcohol and substance misuse	icd
F193	Other psychoactive substance abuse, uncomplicated	Alcohol and substance misuse	icd
F20.0	Paranoid schizophrenia	Mental health	icd
F20.9	Schizophrenia, unspecified	Mental health	icd
F209	Paranoid schizophrenia	Mental health	icd
F22.0	Delusional disorders	Mental health	icd
F29	Unspecified psychosis not due to a substance or known physiological condition	Mental health	icd
F30.9	Manic episode, unspecified	Mental health	icd
F31.1	Bipolar disorder, current episode hypomanic	Mental health	icd
F31.9	Bipolar disorder, unspecified	Mental health	icd
F311	Bipolar disorder, current episode hypomanic	Mental health	icd
F32	Major depressive disorder, single episode, mild	Mental health	icd
F32.20	Major depressive disorder, single episode, mild	Mental health	icd
F32.90	Major depressive disorder, single episode, mild	Mental health	icd
F329	Major depressive disorder, single episode, mild	Mental health	icd
F34.1	Dysthymic disorder	Mental health	icd

ed_code	description	presentation_category	classification
F41.0	Panic disorder [episodic paroxysmal anxiety]	Mental health	icd
F41.9	Anxiety disorder, unspecified	Mental health	icd
F419	Panic disorder [episodic paroxysmal anxiety]	Mental health	icd
F42.8	Other obsessive−compulsive disorder	Mental health	icd
F43.0	Acute stress reaction	Mental health	icd
F43.2	Acute stress reaction	Mental health	icd
F43.9	Reaction to severe stress, unspecified	Mental health	icd
F44.88	Dissociative amnesia	Mental health	icd
F45.2	Somatization disorder	Mental health	icd
F45.4	Somatization disorder	Mental health	icd
F459	Somatization disorder	Mental health	icd
F48.9	Nonpsychotic mental disorder, unspecified	Mental health	icd
F50.0	Anorexia nervosa, unspecified	Mental health	icd
F50.9	Eating disorder, unspecified	Mental health	icd
F60.9	Personality disorder, unspecified	Mental health	icd
F89	Unspecified disorder of psychological development	Mental health	icd
F91.0	Conduct disorder confined to family context	Mental health	icd
F91.8	Other conduct disorders	Mental health	icd
F91.9	Conduct disorder, unspecified	Mental health	icd
F92.9	Mixed disorder of conduct and emotions, unspecified	Mental health	icd
F93.9	Childhood emotional disorder, unspecified	Mental health	icd
F95.9	Tic disorder, unspecified	Mental health	icd
F98.8	Other specified behavioral and emotional disorders with onset usually occurring in childhood and adolescence	Mental health	icd
F99	Mental disorder, not otherwise specified	Mental health	icd
G04.9	Acute disseminated encephalitis and encephalomyelitis, unspecified	Physical illness	icd
G40.9	Localization−related (focal) (partial) idiopathic epilepsy and epileptic syndromes with seizures of localized onset, not intractable, with status epilepticus	Physical illness	icd
G40.90	Localization−related (focal) (partial) idiopathic epilepsy and epileptic syndromes with seizures of localized onset, not intractable, with status epilepticus	Physical illness	icd
G409	Localization−related (focal) (partial) idiopathic epilepsy and epileptic syndromes with seizures of localized onset, not intractable, with status epilepticus	Physical illness	icd
G43.9	Migraine without aura, not intractable, with status migrainosus	Physical illness	icd
G44.2	Cluster headache syndrome, unspecified, intractable	Physical illness	icd
G47.0	Insomnia, unspecified	Physical illness	icd
G47.9	Sleep disorder, unspecified	Physical illness	icd
G60.9	Hereditary and idiopathic neuropathy, unspecified	Physical illness	icd
G93.2	Benign intracranial hypertension	Physical illness	icd
G93.4	Cerebral cysts	Physical illness	icd

ed_code	description	presentation_category	classification
G97.1	Other reaction to spinal and lumbar puncture	Physical illness	icd
H02.9	Unspecified disorder of eyelid	Physical illness	icd
H05.0	Unspecified acute inflammation of orbit	Physical illness	icd
H05.9	Unspecified disorder of orbit	Physical illness	icd
H051	Unspecified acute inflammation of orbit	Physical illness	icd
H10.1	Acute follicular conjunctivitis, right eye	Physical illness	icd
H10.9	Unspecified conjunctivitis	Physical illness	icd
H16.0	Unspecified corneal ulcer, right eye	Physical illness	icd
H16.1	Unspecified corneal ulcer, right eye	Physical illness	icd
H53.9	Unspecified visual disturbance	Physical illness	icd
H57.1	Unspecified anomaly of pupillary function	Physical illness	icd
H57.9	Unspecified disorder of eye and adnexa	Physical illness	icd
H60.3	Abscess of external ear, unspecified ear	Physical illness	icd
H60.9	Abscess of external ear, unspecified ear	Physical illness	icd
H603	Abscess of external ear, unspecified ear	Physical illness	icd
H61.2	Unspecified perichondritis of right external ear	Physical illness	icd
H66.4	Acute suppurative otitis media without spontaneous rupture of ear drum, right ear	Physical illness	icd
H66.9	Acute suppurative otitis media without spontaneous rupture of ear drum, right ear	Physical illness	icd
H92.0	Otalgia, right ear	Physical illness	icd
H93.9	Transient ischemic deafness, right ear	Physical illness	icd
I10	Essential (primary) hypertension	Physical illness	icd
I30.9	Acute pericarditis, unspecified	Physical illness	icd
I31.9	Disease of pericardium, unspecified	Physical illness	icd
I33.0	Acute and subacute infective endocarditis	Physical illness	icd
I49.8	Other specified cardiac arrhythmias	Physical illness	icd
I49.9	Cardiac arrhythmia, unspecified	Physical illness	icd
I51.4	Myocarditis, unspecified	Physical illness	icd
I67.4	Hypertensive encephalopathy	Physical illness	icd
I80.0	Phlebitis and thrombophlebitis of superficial vessels of unspecified lower extremity	Physical illness	icd
J00	Acute nasopharyngitis [common cold]	Physical illness	icd
J01.9	Acute maxillary sinusitis, unspecified	Physical illness	icd
J02.9	Acute pharyngitis, unspecified	Physical illness	icd
J03.9	Acute streptococcal tonsillitis, unspecified	Physical illness	icd
J06.9	Acute upper respiratory infection, unspecified	Physical illness	icd
J069	Acute laryngopharyngitis	Physical illness	icd

ed_code	description	presentation_category	classification
J10.1	Influenza due to other identified influenza virus with other respiratory manifestations	Physical illness	icd
J111	Influenza due to unidentified influenza virus with unspecified type of pneumonia	Physical illness	icd
J18.8	Other pneumonia, unspecified organism	Physical illness	icd
J18.9	Pneumonia, unspecified organism	Physical illness	icd
J181	Bronchopneumonia, unspecified organism	Physical illness	icd
J20.9	Acute bronchitis, unspecified	Physical illness	icd
J22	Unspecified acute lower respiratory infection	Physical illness	icd
J304	Vasomotor rhinitis	Physical illness	icd
J34.8	Abscess, furuncle and carbuncle of nose	Physical illness	icd
J45.9	Mild intermittent asthma, uncomplicated	Physical illness	icd
J46	Unspecified chronic bronchitis	Physical illness	icd
J69.0	Pneumonitis due to inhalation of food and vomit	Physical illness	icd
J80	Acute respiratory distress syndrome	Physical illness	icd
J90	Pleural effusion, not elsewhere classified	Physical illness	icd
J93.1	Spontaneous tension pneumothorax	Physical illness	icd
J93.9	Pneumothorax, unspecified	Physical illness	icd
J98.8	Other specified respiratory disorders	Physical illness	icd
J98.9	Respiratory disorder, unspecified	Physical illness	icd
K02.9	Dental caries, unspecified	Physical illness	icd
K04.4	Acute apical periodontitis of pulpal origin	Physical illness	icd
K04.7	Periapical abscess without sinus	Physical illness	icd
K07.6	Temporomandibular joint disorders	Physical illness	icd
K08.9	Disorder of teeth and supporting structures, unspecified	Physical illness	icd
K0888	Exfoliation of teeth due to systemic causes	Physical illness	icd
K10.9	Disease of jaws, unspecified	Physical illness	icd
K11.2	Atrophy of salivary gland	Physical illness	icd
K12.2	Cellulitis and abscess of mouth	Physical illness	icd
K13.7	Diseases of lips	Physical illness	icd
K20	Eosinophilic esophagitis	Physical illness	icd
K21.9	Gastro−esophageal reflux disease without esophagitis	Physical illness	icd
K27.0	Acute peptic ulcer, site unspecified, with hemorrhage	Physical illness	icd
K29.1	Acute gastritis without bleeding	Physical illness	icd
K29.90	Gastroduodenitis, unspecified, without bleeding	Physical illness	icd
K35.8	Acute appendicitis with generalized peritonitis, without abscess	Physical illness	icd
K35.9	Acute appendicitis with generalized peritonitis, without abscess	Physical illness	icd

ed_code	description	presentation_category	classification
K358	Acute appendicitis with generalized peritonitis, without abscess	Physical illness	icd
K359	Acute appendicitis with generalized peritonitis, without abscess	Physical illness	icd
K46.9	Unspecified abdominal hernia without obstruction or gangrene	Physical illness	icd
K52.8	Gastroenteritis and colitis due to radiation	Physical illness	icd
K52.9	Noninfective gastroenteritis and colitis, unspecified	Physical illness	icd
K529	Gastroenteritis and colitis due to radiation	Physical illness	icd
K56.6	Paralytic ileus	Physical illness	icd
K57.32	Diverticulitis of large intestine without perforation or abscess without bleeding	Physical illness	icd
K58.9	Irritable bowel syndrome without diarrhea	Physical illness	icd
K59.0	Constipation, unspecified	Physical illness	icd
K60.2	Anal fissure, unspecified	Physical illness	icd
K61.0	Anal abscess	Physical illness	icd
K61.1	Rectal abscess	Physical illness	icd
K62.5	Hemorrhage of anus and rectum	Physical illness	icd
K62.8	Anal polyp	Physical illness	icd
K63.1	Perforation of intestine (nontraumatic)	Physical illness	icd
K64.9	Unspecified hemorrhoids	Physical illness	icd
K80.20	Calculus of gallbladder without cholecystitis without obstruction	Physical illness	icd
K8050	Calculus of gallbladder with acute cholecystitis without obstruction	Physical illness	icd
K85.9	Idiopathic acute pancreatitis without necrosis or infection	Physical illness	icd
K92.1	Melena	Physical illness	icd
K92.2	Gastrointestinal hemorrhage, unspecified	Physical illness	icd
L01.0	Impetigo, unspecified	Physical illness	icd
L02.40	Cutaneous abscess, furuncle and carbuncle	Physical illness	icd
L03.10	Cellulitis of right finger	Physical illness	icd
L03.11	Cellulitis of right finger	Physical illness	icd
L03.12	Cellulitis of right finger	Physical illness	icd
L03.14	Cellulitis of right finger	Physical illness	icd
L03.3	Cellulitis of right finger	Physical illness	icd
L03.9	Cellulitis of right finger	Physical illness	icd
L0301C	Cellulitis of right finger	Physical illness	icd
L05.0	Pilonidal cyst with abscess	Physical illness	icd
L08.9	Local infection of the skin and subcutaneous tissue, unspecified	Physical illness	icd
L089CD	Pyoderma	Physical illness	icd
L27.0	Generalized skin eruption due to drugs and medicaments taken internally	Physical illness	icd

ed_code	description	presentation_category	classification
L29.9	Pruritus, unspecified	Physical illness	icd
L50.9	Urticaria, unspecified	Physical illness	icd
L60.0	Ingrowing nail	Physical illness	icd
L98.9	Disorder of the skin and subcutaneous tissue, unspecified	Physical illness	icd
M00.97	Staphylococcal arthritis, unspecified joint	Physical illness	icd
M10.99	Idiopathic gout, unspecified site	Physical illness	icd
M20.0	Unspecified deformity of right finger(s)	Physical illness	icd
M23.89	Cystic meniscus, unspecified lateral meniscus, right knee	Physical illness	icd
M25.46	Hemarthrosis, unspecified joint	Accident and injury	icd
M25.49	Hemarthrosis, unspecified joint	Accident and injury	icd
M25.51	Hemarthrosis, unspecified joint	Accident and injury	icd
M25.52	Hemarthrosis, unspecified joint	Accident and injury	icd
M25.53	Hemarthrosis, unspecified joint	Accident and injury	icd
M25.54	Hemarthrosis, unspecified joint	Accident and injury	icd
M25.55	Hemarthrosis, unspecified joint	Accident and injury	icd
M25.56	Hemarthrosis, unspecified joint	Accident and injury	icd
M25.57	Hemarthrosis, unspecified joint	Accident and injury	icd
M25.59	Hemarthrosis, unspecified joint	Accident and injury	icd
M2551	Hemarthrosis, unspecified joint	Accident and injury	icd
M2553A	Hemarthrosis, unspecified joint	Accident and injury	icd
M2554	Hemarthrosis, unspecified joint	Accident and injury	icd
M2557	Hemarthrosis, unspecified joint	Accident and injury	icd
M2559	Hemarthrosis, unspecified joint	Accident and injury	icd
M43.6	Torticollis	Mental health	icd
M51.2	Intervertebral disc disorders with myelopathy, thoracic region	Physical illness	icd
M53.3	Sacrococcygeal disorders, not elsewhere classified	Physical illness	icd
M54.2	Cervicalgia	Physical illness	icd
M54.5	Low back pain	Physical illness	icd
M54.9	Dorsalgia, unspecified	Physical illness	icd
M54.99	Panniculitis affecting regions of neck and back, site unspecified	Physical illness	icd
M545	Panniculitis affecting regions of neck and back, site unspecified	Physical illness	icd
M66.0	Rupture of popliteal cyst	Physical illness	icd
M70.2	Crepitant synovitis (acute) (chronic), right wrist	Physical illness	icd
M75.1	Adhesive capsulitis of unspecified shoulder	Physical illness	icd
M79.19	Rheumatism, unspecified	Physical illness	icd

ed_code	description	presentation_category	classification
M79.29	Rheumatism, unspecified	Physical illness	icd
M79.69	Rheumatism, unspecified	Physical illness	icd
M79.99	Rheumatism, unspecified	Physical illness	icd
M7969	Rheumatism, unspecified	Physical illness	icd
M85.89	Other specified disorders of bone density and structure, multiple sites	Physical illness	icd
M86.19	Other acute osteomyelitis, multiple sites	Physical illness	icd
M95.9	Acquired deformity of musculoskeletal system, unspecified	Physical illness	icd
N10	Acute pyelonephritis	Physical illness	icd
N12	Tubulo−interstitial nephritis, not specified as acute or chronic	Physical illness	icd
N23	Unspecified renal colic	Physical illness	icd
N30.9	Acute cystitis without hematuria	Physical illness	icd
N39.0	Urinary tract infection, site not specified	Physical illness	icd
N39.9	Disorder of urinary system, unspecified	Physical illness	icd
N390	Urinary tract infection, site not specified	Physical illness	icd
N43.3	Hydrocele, unspecified	Physical illness	icd
N45.9	Epididymitis	Physical illness	icd
N48.9	Disorder of penis, unspecified	Physical illness	icd
N50.9	Disorder of male genital organs, unspecified	Physical illness	icd
N60.1	Solitary cyst of right breast	Physical illness	icd
N61	Mastitis without abscess	Physical illness	icd
N63	Unspecified lump in unspecified breast	Physical illness	icd
N73.9	Female pelvic inflammatory disease, unspecified	Physical illness	icd
N739	Acute parametritis and pelvic cellulitis	Physical illness	icd
N75.0	Cyst of Bartholin's gland	Physical illness	icd
N80.9	Endometriosis, unspecified	Physical illness	icd
N83.2	Follicular cyst of ovary, unspecified side	Physical illness	icd
N89.8	Other specified noninflammatory disorders of vagina	Physical illness	icd
N92.6	Irregular menstruation, unspecified	Physical illness	icd
N93.9	Abnormal uterine and vaginal bleeding, unspecified	Physical illness	icd
N94.6	Dysmenorrhea, unspecified	Physical illness	icd
N946	Mittelschmerz	Physical illness	icd
O009	Abdominal pregnancy without intrauterine pregnancy	Physical illness	icd
O02.1	Missed abortion	Physical illness	icd
O03.4	Incomplete spontaneous abortion without complication	Physical illness	icd
O03.9	Complete or unspecified spontaneous abortion without complication	Physical illness	icd

ed_code	description	presentation_category	classification
O20.0	Threatened abortion	Physical illness	icd
O20.9	Hemorrhage in early pregnancy, unspecified	Physical illness	icd
O21.9	Vomiting of pregnancy, unspecified	Physical illness	icd
O210	Mild hyperemesis gravidarum	Physical illness	icd
O26.9	Excessive weight gain in pregnancy, unspecified trimester	Physical illness	icd
O469	Antepartum hemorrhage with coagulation defect, unspecified, first trimester	Physical illness	icd
O80	Encounter for full−term uncomplicated delivery	Physical illness	icd
Q898	Asplenia (congenital)	Physical illness	icd
R00.0	Tachycardia, unspecified	Physical illness	icd
R00.2	Palpitations	Physical illness	icd
R04.0	Epistaxis	Physical illness	icd
R04.2	Hemoptysis	Physical illness	icd
R05	Cough	Physical illness	icd
R06.0	Dyspnea, unspecified	Physical illness	icd
R06.8	Dyspnea, unspecified	Physical illness	icd
R060	Dyspnea, unspecified	Physical illness	icd
R07.0	Pain in throat	Physical illness	icd
R07.1	Chest pain on breathing	Physical illness	icd
R07.3	Pain in throat	Physical illness	icd
R07.4	Pain in throat	Physical illness	icd
R074	Pain in throat	Physical illness	icd
R09.1	Pleurisy	Physical illness	icd
R091	Asphyxia	Accident and injury	icd
R10.0	Severe abdominal pain	Physical illness	icd
R10.1	Acute abdomen	Physical illness	icd
R10.2	Pelvic and perineal pain	Physical illness	icd
R10.3	Acute abdomen	Physical illness	icd
R10.4	Acute abdomen	Physical illness	icd
R101	Acute abdomen	Physical illness	icd
R104	Acute abdomen	Physical illness	icd
R11	Nausea	Physical illness	icd
R13	Aphagia	Physical illness	icd
R190	Intra−abdominal and pelvic swelling, mass and lump, unspecified site	Physical illness	icd
R20.8	Other disturbances of skin sensation	Physical illness	icd
R21	Rash and other nonspecific skin eruption	Physical illness	icd

ed_code	description	presentation_category	classification
R22.0	Localized swelling, mass and lump, head	Physical illness	icd
R22.9	Localized swelling, mass and lump, unspecified	Physical illness	icd
R25.2	Cramp and spasm	Physical illness	icd
R25.8	Other abnormal involuntary movements	Physical illness	icd
R26.8	Ataxic gait	Physical illness	icd
R30.0	Dysuria	Physical illness	icd
R31	Gross hematuria	Physical illness	icd
R32	Unspecified urinary incontinence	Physical illness	icd
R33	Drug induced retention of urine	Physical illness	icd
R35	Frequency of micturition	Physical illness	icd
R41.0	Disorientation, unspecified	Physical illness	icd
R42	Dizziness and giddiness	Physical illness	icd
R44.3	Hallucinations, unspecified	Mental health	icd
R45.1	Restlessness and agitation	Mental health	icd
R45.81	Low self−esteem	Mental health	icd
R47.8	Aphasia	Physical illness	icd
R50.8	Drug induced fever	Physical illness	icd
R50.9	Fever, unspecified	Physical illness	icd
R509	Drug induced fever	Physical illness	icd
R51	Headache	Physical illness	icd
R52.0	Pain, unspecified	Physical illness	icd
R52.1	Pain, unspecified	Physical illness	icd
R52.9	Pain, unspecified	Physical illness	icd
R53	Neoplastic (malignant) related fatigue	Physical illness	icd
R55	Syncope and collapse	Physical illness	icd
R56.0	Simple febrile convulsions	Physical illness	icd
R56.8	Simple febrile convulsions	Physical illness	icd
R59.9	Enlarged lymph nodes, unspecified	Physical illness	icd
R60.0	Localized edema	Physical illness	icd
R60.9	Edema, unspecified	Physical illness	icd
R63.4	Abnormal weight loss	Physical illness	icd
R68.8	Hypothermia, not associated with low environmental temperature	Physical illness	icd
R73	Impaired fasting glucose	Physical illness	icd
S00.01	Unspecified superficial injury of scalp, initial encounter	Accident and injury	icd
S00.90	Unspecified superficial injury of scalp, initial encounter	Accident and injury	icd

ed_code	description	presentation_category	classification
S00.95	Unspecified superficial injury of scalp, initial encounter	Accident and injury	icd
S01.0	Unspecified open wound of scalp, initial encounter	Accident and injury	icd
S01.20	Unspecified open wound of scalp, initial encounter	Accident and injury	icd
S01.30	Unspecified open wound of scalp, initial encounter	Accident and injury	icd
S01.88	Unspecified open wound of scalp, initial encounter	Accident and injury	icd
S0180	Unspecified open wound of scalp, initial encounter	Accident and injury	icd
S019	Unspecified open wound of scalp, initial encounter	Accident and injury	icd
S02.0	Fracture of vault of skull, initial encounter for closed fracture	Accident and injury	icd
S02.2	Fracture of vault of skull, initial encounter for closed fracture	Accident and injury	icd
S02.5	Fracture of vault of skull, initial encounter for closed fracture	Accident and injury	icd
S02.60	Fracture of vault of skull, initial encounter for closed fracture	Accident and injury	icd
S02.9	Fracture of vault of skull, initial encounter for closed fracture	Accident and injury	icd
S0260	Fracture of mandible	Accident and injury	icd
S05.0	Injury of conjunctiva and corneal abrasion without foreign body, unspecified eye, initial encounter	Accident and injury	icd
S05.8	Injury of conjunctiva and corneal abrasion without foreign body, unspecified eye, initial encounter	Accident and injury	icd
S06.00	Concussion without loss of consciousness, initial encounter	Accident and injury	icd
S06.01	Concussion without loss of consciousness, initial encounter	Accident and injury	icd
S06.8	Concussion without loss of consciousness, initial encounter	Accident and injury	icd
S09.9	Injury of blood vessels of head, not elsewhere classified, initial encounter	Accident and injury	icd
S099	Injury of blood vessels of head, not elsewhere classified, initial encounter	Accident and injury	icd
S11.88	Laceration without foreign body of larynx, initial encounter	Accident and injury	icd
S13.4	Traumatic rupture of cervical intervertebral disc, initial encounter	Accident and injury	icd
S13.6	Traumatic rupture of cervical intervertebral disc, initial encounter	Accident and injury	icd
S19.9	Other specified injuries of unspecified part of neck, initial encounter	Accident and injury	icd
S20.2	Contusion of breast, unspecified breast, initial encounter	Accident and injury	icd
S21.1	Unspecified open wound of right breast, initial encounter	Accident and injury	icd
S21.2	Unspecified open wound of right breast, initial encounter	Accident and injury	icd
S22.40	Wedge compression fracture of unspecified thoracic vertebra, initial encounter for closed fracture	Accident and injury	icd
S23.4	Traumatic rupture of thoracic intervertebral disc, initial encounter	Accident and injury	icd
S27.0	Traumatic pneumothorax, initial encounter	Accident and injury	icd
S31.0	Unspecified open wound of lower back and pelvis without penetration into retroperitoneum, initial encounter	Accident and injury	icd
S31.3	Unspecified open wound of lower back and pelvis without penetration into retroperitoneum, initial encounter	Accident and injury	icd
S31.4	Unspecified open wound of lower back and pelvis without penetration into retroperitoneum, initial encounter	Accident and injury	icd
S33.7	Traumatic rupture of lumbar intervertebral disc, initial encounter	Accident and injury	icd
S40.82	Contusion of right shoulder, initial encounter	Accident and injury	icd

ed_code	description	presentation_category	classification
S40.9	Contusion of right shoulder, initial encounter	Accident and injury	icd
S41.0	Unspecified open wound of right shoulder, initial encounter	Accident and injury	icd
S42.00	Fracture of unspecified part of right clavicle, initial encounter for closed fracture	Accident and injury	icd
S42.10	Fracture of unspecified part of right clavicle, initial encounter for closed fracture	Accident and injury	icd
S42.20	Fracture of unspecified part of right clavicle, initial encounter for closed fracture	Accident and injury	icd
S42.3	Fracture of unspecified part of right clavicle, initial encounter for closed fracture	Accident and injury	icd
S42.40	Fracture of unspecified part of right clavicle, initial encounter for closed fracture	Accident and injury	icd
S43.00	Unspecified subluxation of right shoulder joint, initial encounter	Accident and injury	icd
S43.3	Unspecified subluxation of right shoulder joint, initial encounter	Accident and injury	icd
S43.4	Unspecified subluxation of right shoulder joint, initial encounter	Accident and injury	icd
S43.7	Unspecified subluxation of right shoulder joint, initial encounter	Accident and injury	icd
S433	Unspecified subluxation of right shoulder joint, initial encounter	Accident and injury	icd
S437	Unspecified subluxation of right shoulder joint, initial encounter	Accident and injury	icd
S49.9	Unspecified physeal fracture of upper end of humerus, right arm, initial encounter for closed fracture	Accident and injury	icd
S498	Unspecified physeal fracture of upper end of humerus, right arm, initial encounter for closed fracture	Accident and injury	icd
S50.0	Contusion of unspecified elbow, initial encounter	Accident and injury	icd
S50.81	Contusion of unspecified elbow, initial encounter	Accident and injury	icd
S50.88	Contusion of unspecified elbow, initial encounter	Accident and injury	icd
S50.9	Contusion of unspecified elbow, initial encounter	Accident and injury	icd
S51.0	Unspecified open wound of right elbow, initial encounter	Accident and injury	icd
S51.9	Unspecified open wound of right elbow, initial encounter	Accident and injury	icd
S52.50	Unspecified fracture of upper end of right ulna, initial encounter for closed fracture	Accident and injury	icd
S52.51	Unspecified fracture of upper end of right ulna, initial encounter for closed fracture	Accident and injury	icd
S52.9	Unspecified fracture of upper end of right ulna, initial encounter for closed fracture	Accident and injury	icd
S53.10	Dislocation of elbow, unspecified	Accident and injury	icd
S53.40	Unspecified subluxation of right radial head, initial encounter	Accident and injury	icd
S59.9	Unspecified physeal fracture of lower end of ulna, right arm, initial encounter for closed fracture	Accident and injury	icd
S60.1	Contusion of unspecified finger without damage to nail, initial encounter	Accident and injury	icd
S60.84	Contusion of unspecified finger without damage to nail, initial encounter	Accident and injury	icd
S60.88	Contusion of unspecified finger without damage to nail, initial encounter	Accident and injury	icd
S60.9	Contusion of unspecified finger without damage to nail, initial encounter	Accident and injury	icd
S6081A	Contusion of unspecified finger without damage to nail, initial encounter	Accident and injury	icd
S61.0	Unspecified open wound of right thumb without damage to nail, initial encounter	Accident and injury	icd
S61.81	Unspecified open wound of right thumb without damage to nail, initial encounter	Accident and injury	icd
S61.9	Unspecified open wound of right thumb without damage to nail, initial encounter	Accident and injury	icd

ed_code	description	presentation_category	classification
S610	Unspecified open wound of right thumb without damage to nail, initial encounter	Accident and injury	icd
S6181B	Unspecified open wound of right thumb without damage to nail, initial encounter	Accident and injury	icd
S62.0	Unspecified fracture of navicular [scaphoid] bone of right wrist, initial encounter for closed fracture	Accident and injury	icd
S62.10	Unspecified fracture of navicular [scaphoid] bone of right wrist, initial encounter for closed fracture	Accident and injury	icd
S62.30	Unspecified fracture of navicular [scaphoid] bone of right wrist, initial encounter for closed fracture	Accident and injury	icd
S62.61	Unspecified fracture of navicular [scaphoid] bone of right wrist, initial encounter for closed fracture	Accident and injury	icd
S6230	Unspecified fracture of navicular [scaphoid] bone of right wrist, initial encounter for closed fracture	Accident and injury	icd
S63.10	Unspecified subluxation of right wrist and hand, initial encounter	Accident and injury	icd
S63.50	Unspecified subluxation of right wrist and hand, initial encounter	Accident and injury	icd
S63.60	Unspecified subluxation of right wrist and hand, initial encounter	Accident and injury	icd
S63.7	Unspecified subluxation of right wrist and hand, initial encounter	Accident and injury	icd
S6350	Unspecified subluxation of right wrist and hand, initial encounter	Accident and injury	icd
S68.0	Complete traumatic metacarpophalangeal amputation of right thumb, initial encounter	Accident and injury	icd
S68.1	Complete traumatic metacarpophalangeal amputation of right thumb, initial encounter	Accident and injury	icd
S69.9	Other specified injuries of unspecified wrist, hand and finger(s), initial encounter	Accident and injury	icd
S699	Other specified injuries of unspecified wrist, hand and finger(s), initial encounter	Accident and injury	icd
S70.81	Contusion of unspecified hip, initial encounter	Accident and injury	icd
S70.83	Contusion of unspecified hip, initial encounter	Accident and injury	icd
S70.88	Other superficial injuries of hip and thigh	Accident and injury	icd
S71.0	Open wound of hip and thigh	Accident and injury	icd
S71.1	Unspecified open wound, right hip, initial encounter	Accident and injury	icd
S73.00	Unspecified subluxation of right hip, initial encounter	Accident and injury	icd
S73.10	Unspecified subluxation of right hip, initial encounter	Accident and injury	icd
S80.0	Contusion of unspecified knee, initial encounter	Accident and injury	icd
S80.1	Contusion of unspecified knee, initial encounter	Accident and injury	icd
S81.0	Unspecified open wound, right knee, initial encounter	Accident and injury	icd
S81.9	Unspecified open wound, right knee, initial encounter	Accident and injury	icd
S82.0	Unspecified fracture of right patella, initial encounter for closed fracture	Accident and injury	icd
S82.28	Unspecified fracture of right patella, initial encounter for closed fracture	Accident and injury	icd
S82.6	Unspecified fracture of right patella, initial encounter for closed fracture	Accident and injury	icd
S82.88	Unspecified fracture of right patella, initial encounter for closed fracture	Accident and injury	icd
S825	Unspecified fracture of right patella, initial encounter for closed fracture	Accident and injury	icd
S83.0	Unspecified subluxation of right patella, initial encounter	Accident and injury	icd
S83.10	Unspecified subluxation of right patella, initial encounter	Accident and injury	icd
S83.6	Unspecified subluxation of right patella, initial encounter	Accident and injury	icd

ed_code	description	presentation_category	classification
S8310	Unspecified subluxation of right patella, initial encounter	Accident and injury	icd
S89.9	Unspecified physeal fracture of upper end of right tibia, initial encounter for closed fracture	Accident and injury	icd
S899	Unspecified physeal fracture of upper end of right tibia, initial encounter for closed fracture	Accident and injury	icd
S90.0	Contusion of unspecified ankle, initial encounter	Accident and injury	icd
S90.81	Contusion of unspecified ankle, initial encounter	Accident and injury	icd
S90.84	Contusion of unspecified ankle, initial encounter	Accident and injury	icd
S90.88	Contusion of unspecified ankle, initial encounter	Accident and injury	icd
S90.9	Contusion of unspecified ankle, initial encounter	Accident and injury	icd
S91.0	Unspecified open wound, right ankle, initial encounter	Accident and injury	icd
S91.1	Unspecified open wound, right ankle, initial encounter	Accident and injury	icd
S91.3	Unspecified open wound, right ankle, initial encounter	Accident and injury	icd
S92.4	Unspecified fracture of right calcaneus, initial encounter for closed fracture	Accident and injury	icd
S92.5	Unspecified fracture of right calcaneus, initial encounter for closed fracture	Accident and injury	icd
S92.9	Unspecified fracture of right calcaneus, initial encounter for closed fracture	Accident and injury	icd
S924	Fracture of great toe	Accident and injury	icd
S93.40	Subluxation of right ankle joint, initial encounter	Accident and injury	icd
S93.48	Subluxation of right ankle joint, initial encounter	Accident and injury	icd
S93.6	Subluxation of right ankle joint, initial encounter	Accident and injury	icd
S94.3	Injury of lateral plantar nerve, unspecified leg, initial encounter	Accident and injury	icd
S97.1	Crushing injury of unspecified ankle, initial encounter	Accident and injury	icd
S971	Crushing injury of unspecified ankle, initial encounter	Accident and injury	icd
S99.9	Unspecified physeal fracture of right calcaneus, initial encounter for closed fracture	Accident and injury	icd
T00.0	Superficial injuries involving multiple body regions	Accident and injury	icd
T01.9	Multiple open wounds, unspecified	Accident and injury	icd
T07	Unspecified multiple injuries, initial encounter	Accident and injury	icd
T09.00	Other injuries of spine and trunk, level unspecified	Accident and injury	icd
T13.1	Other injuries of lower limb, level unspecified	Accident and injury	icd
T14.03	Other injury of unspecified body region, initial encounter	Accident and injury	icd
T14.04	Other injury of unspecified body region, initial encounter	Accident and injury	icd
T14.05	Other injury of unspecified body region, initial encounter	Accident and injury	icd
T14.1	Other injury of unspecified body region, initial encounter	Accident and injury	icd
T14.20	Other injury of unspecified body region, initial encounter	Accident and injury	icd
T14.3	Other injury of unspecified body region, initial encounter	Accident and injury	icd
T14.4	Other injury of unspecified body region, initial encounter	Accident and injury	icd
T14.9	Other injury of unspecified body region, initial encounter	Accident and injury	icd

ed_code	description	presentation_category	classification
T149	Other injury of unspecified body region, initial encounter	Accident and injury	icd
T15.0	Foreign body in cornea, unspecified eye, initial encounter	Accident and injury	icd
T15.1	Foreign body in cornea, unspecified eye, initial encounter	Accident and injury	icd
T16	Foreign body in right ear, initial encounter	Accident and injury	icd
T18.1	Foreign body in mouth, initial encounter	Accident and injury	icd
T20.0	Burn of unspecified degree of head, face, and neck, unspecified site, initial encounter	Accident and injury	icd
T20.2	Burn of unspecified degree of head, face, and neck, unspecified site, initial encounter	Accident and injury	icd
T21.00	Burn of unspecified degree of trunk, unspecified site, initial encounter	Accident and injury	icd
T2119	Burn of unspecified degree of trunk, unspecified site, initial encounter	Accident and injury	icd
T22.00	Burn of unspecified degree of shoulder and upper limb, except wrist and hand, unspecified site, initial encounter	Accident and injury	icd
T22.10	Burn of unspecified degree of shoulder and upper limb, except wrist and hand, unspecified site, initial encounter	Accident and injury	icd
T23.0	Burn of unspecified degree of right hand, unspecified site, initial encounter	Accident and injury	icd
T23.2	Burn of unspecified degree of right hand, unspecified site, initial encounter	Accident and injury	icd
T24.0	Burn of unspecified degree of unspecified site of right lower limb, except ankle and foot, initial encounter	Accident and injury	icd
T25.0	Burn of unspecified degree of right ankle, initial encounter	Physical illness	icd
T26.4	Burn of unspecified eyelid and periocular area, initial encounter	Accident and injury	icd
T29.0	Burns and corrosions of multiple body regions	Accident and injury	icd
T291	Burns of multiple regions, no more than first−degree burns mentioned	Accident and injury	icd
T30.2	Burn of unspecified body region, unspecified degree	Accident and injury	icd
T30.3	Burn of third degree, body region unspecified	Accident and injury	icd
T39.1	Poisoning by aspirin, accidental (unintentional), initial encounter	Accident and injury	icd
T39.4	Poisoning by aspirin, accidental (unintentional), initial encounter	Accident and injury	icd
T39.9	Poisoning by aspirin, accidental (unintentional), initial encounter	Accident and injury	icd
T40.0	Poisoning by opium, accidental (unintentional), initial encounter	Alcohol and substance misuse	icd
T40.1	Poisoning by opium, accidental (unintentional), initial encounter	Alcohol and substance misuse	icd
T40.5	Poisoning by opium, accidental (unintentional), initial encounter	Alcohol and substance misuse	icd
T40.7	Poisoning by opium, accidental (unintentional), initial encounter	Alcohol and substance misuse	icd
T41.21	Poisoning by inhaled anesthetics, accidental (unintentional), initial encounter	Accident and injury	icd
T41.22	Poisoning by inhaled anesthetics, accidental (unintentional), initial encounter	Accident and injury	icd
T414	Poisoning by inhaled anesthetics, accidental (unintentional), initial encounter	Accident and injury	icd
T42.4	Poisoning by hydantoin derivatives, accidental (unintentional), initial encounter	Accident and injury	icd
T42.6	Poisoning by hydantoin derivatives, accidental (unintentional), initial encounter	Accident and injury	icd
T42.7	Poisoning by hydantoin derivatives, accidental (unintentional), initial encounter	Accident and injury	icd
T43.0	Poisoning by tricyclic antidepressants, accidental (unintentional), initial encounter	Suicidal behaviours and self−harm	icd
T43.60	Poisoning by tricyclic antidepressants, accidental (unintentional), initial encounter	Suicidal behaviours and self−harm	icd

ed_code	description	presentation_category	classification
T43.61	Poisoning by tricyclic antidepressants, accidental (unintentional), initial encounter	Suicidal behaviours and self−harm	icd
T43.62	Poisoning by tricyclic antidepressants, accidental (unintentional), initial encounter	Suicidal behaviours and self−harm	icd
T43.9	Poisoning by tricyclic antidepressants, accidental (unintentional), initial encounter	Suicidal behaviours and self−harm	icd
T44.3	Poisoning by anticholinesterase agents, accidental (unintentional), initial encounter	Accident and injury	icd
T44.9	Poisoning by anticholinesterase agents, accidental (unintentional), initial encounter	Accident and injury	icd
T46.0	Poisoning by cardiac−stimulant glycosides and drugs of similar action, accidental (unintentional), initial encounter	Accident and injury	icd
T46.9	Poisoning by cardiac−stimulant glycosides and drugs of similar action, accidental (unintentional), initial encounter	Accident and injury	icd
T50.9	Poisoning by mineralocorticoids and their antagonists, accidental (unintentional), initial encounter	Accident and injury	icd
T54.0	Toxic effect of phenol and phenol homologues, accidental (unintentional), initial encounter	Accident and injury	icd
T59.8	Toxic effect of nitrogen oxides, accidental (unintentional), initial encounter	Alcohol and substance misuse	icd
T65.9	Toxic effect of cyanides, accidental (unintentional), initial encounter	Accident and injury	icd
T67.0	Heatstroke and sunstroke, initial encounter	Physical illness	icd
T67.1	Heatstroke and sunstroke, initial encounter	Physical illness	icd
T74.9	Adult neglect or abandonment, confirmed, initial encounter	Other	icd
T749A	Adult neglect or abandonment, confirmed, initial encounter	Other	icd
T75.4	Unspecified effects of lightning, initial encounter	Accident and injury	icd
T78.2	Anaphylactic reaction due to unspecified food, initial encounter	Physical illness	icd
T78.4	Anaphylactic reaction due to unspecified food, initial encounter	Physical illness	icd
T79.3	Air embolism (traumatic), initial encounter	Accident and injury	icd
T79.6	Air embolism (traumatic), initial encounter	Accident and injury	icd
T80.5	Air embolism following infusion, transfusion and therapeutic injection, initial encounter	Physical illness	icd
T81.41	Postprocedural shock unspecified, initial encounter	Physical illness	icd
T81.8	Postprocedural shock unspecified, initial encounter	Physical illness	icd
T82.3	Breakdown (mechanical) of heart valve prosthesis, initial encounter	Physical illness	icd
T85.9	Breakdown (mechanical) of ventricular intracranial (communicating) shunt, initial encounter	Physical illness	icd
T88.1	Infection following immunization, initial encounter	Physical illness	icd
T88.7	Infection following immunization, initial encounter	Physical illness	icd
T89.00	Unspecified complication of procedure	Physical illness	icd
U559	NA	NA	icd
U7300	NA	NA	icd
V01.8	Contact with or exposure to cholera	Physical illness	icd
V22.2	Pregnant state, incidental	Physical illness	icd
V49.9	Unspecified problems with limbs and other problems	Physical illness	icd
V54.0	Encounter for removal of internal fixation device	Physical illness	icd
V54.8	Encounter for removal of internal fixation device	Physical illness	icd

ed_code	description	presentation_category	classification
V58.3	Encounter for radiotherapy	Physical illness	icd
V62.6	Refusal of treatment for reasons of religion or conscience	Other	icd
V62.9	Unspecified psychosocial circumstance	Mental health	icd
V65.9	Unspecified reason for consultation	Other	icd
V67.5	Follow−up examination, following surgery, unspecified	Other	icd
V67.9	Unspecified follow−up examination	Other	icd
V68.0	Disability examination	Other	icd
V68.1	Issue of repeat prescriptions	Other	icd
V68.81	Referral of patient without examination or treatment	Other	icd
V71.01	Observation for adult antisocial behavior	Mental health	icd
V71.09	Observation for other suspected mental condition	Mental health	icd
V71.4	Observation following other accident	Accident and injury	icd
V71.5	Observation following alleged rape or seduction	Accident and injury	icd
V71.8	Observation for adult antisocial behavior	Mental health	icd
V72.6	Examination of eyes and vision	Physical illness	icd
V80.00	Special screening for traumatic brain injury	Accident and injury	icd
V82.9	Screening for unspecified condition	Other	icd
V89.9	Suspected problem with amniotic cavity and membrane not found	Physical illness	icd
W19	Unspecified fall, initial encounter	Accident and injury	icd
X21.9	Contact with venomous spiders	Accident and injury	icd
X84	Intentional self−harm by unspecified means	Suicidal behaviours and self−harm	icd
X99.99	Assault by sharp glass, initial encounter	Accident and injury	icd
Y04.09	Assault by unarmed brawl or fight, initial encounter	Accident and injury	icd
Y05.89	Sexual assault by bodily force	Accident and injury	icd
Y08.09	Assault by strike by hockey stick, initial encounter	Accident and injury	icd
Y09	Assault by unspecified means	Accident and injury	icd
Y09.05	Assault by unspecified means	Accident and injury	icd
Z000	Encounter for general adult medical examination without abnormal findings	Physical illness	icd
Z01.6	Encounter for examination of eyes and vision without abnormal findings	Physical illness	icd
Z01.7	Encounter for examination of eyes and vision without abnormal findings	Physical illness	icd
Z02.7	Encounter for examination for admission to educational institution	Other	icd
Z03.2	Encounter for observation for suspected toxic effect from ingested substance ruled out	Physical illness	icd
Z03.8	Encounter for observation for suspected toxic effect from ingested substance ruled out	Physical illness	icd
Z04.0	Encounter for examination and observation following transport accident	Accident and injury	icd
Z04.3	Encounter for examination and observation following other accident	Accident and injury	icd

ed_code	description	presentation_category	classification
Z04.4	Encounter for examination and observation following transport accident	Accident and injury	icd
Z04.5	Encounter for examination and observation following transport accident	Accident and injury	icd
Z04.8	Encounter for examination and observation following transport accident	Accident and injury	icd
Z043	Encounter for examination and observation following transport accident	Accident and injury	icd
Z043A	Encounter for examination and observation following transport accident	Accident and injury	icd
Z09.9	Encounter for follow−up examination after completed treatment for conditions other than malignant neoplasm	Physical illness	icd
Z11.5	Encounter for screening for intestinal infectious diseases	Physical illness	icd
Z29.9	Encounter for prophylactic measures, unspecified	Other	icd
Z32	Encounter for pregnancy test, result unknown	Physical illness	icd
Z32.0	Encounter for pregnancy test, result unknown	Physical illness	icd
Z33	Pregnant state, incidental	Physical illness	icd
Z47.8	Aftercare following joint replacement surgery	Physical illness	icd
Z48.0	Encounter for change or removal of nonsurgical wound dressing	Accident and injury	icd
Z480	Encounter for change or removal of nonsurgical wound dressing	Accident and injury	icd
Z51.9	Encounter for antineoplastic radiation therapy	Physical illness	icd
Z53.1	Procedure and treatment not carried out because of patient's decision for reasons of belief and group pressure	Other	icd
Z53.8	Procedure and treatment not carried out for other reasons	Other	icd
Z53.9	Procedure and treatment not carried out, unspecified reason	Other	icd
Z531	Procedure and treatment not carried out due to patient smoking	Other	icd
Z59.0	Homelessness	Other	icd
Z60.1	Problems of adjustment to life−cycle transitions	Mental health	icd
Z65.9	Problem related to unspecified psychosocial circumstances	Mental health	icd
Z71.1	Person with feared health complaint in whom no diagnosis is made	Physical illness	icd
Z72.1	Tobacco use	Alcohol and substance misuse	icd
Z72.2	Tobacco use	Alcohol and substance misuse	icd
Z76.0	Encounter for issue of repeat prescription	Other	icd
Z76.5	Malingerer [conscious simulation]	Mental health	icd
Z76.8	Encounter for issue of repeat prescription	Other	icd
Z86.5	Personal history of in−situ neoplasm of breast	Physical illness	icd
Z91.1	Allergy to peanuts	Physical illness	icd
Z91.5	Personal history of self−harm	Suicidal behaviours and self−harm	icd
Z911	Allergy to peanuts	Physical illness	icd
10001005	Bacterial sepsis	Physical illness	snomed
10128002	Rosacea conjunctivitis	Physical illness	snomed
10132008	Burns of multiple sites	Accident and injury	snomed

ed_code	description	presentation_category	classification
102449007	Tardive dyskinesia	Mental health	snomed
102453009	Peritonsillar cellulitis	Physical illness	snomed
102512003	Healthy adult	Other	snomed
102550009	Muscle cramps in leg	Physical illness	snomed
102556003	Pain in upper limb	Physical illness	snomed
102570003	Inguinal pain	Physical illness	snomed
102575008	Oedema of knee	Physical illness	snomed
102587001	Acute chest pain	Physical illness	snomed
102588006	Chest wall pain	Physical illness	snomed
102589003	Atypical chest pain	Physical illness	snomed
102590007	Intermittent palpitations	Physical illness	snomed
102591006	Chest wall tenderness	Physical illness	snomed
102592004	ECG finding	Physical illness	snomed
102594003	Abnormal ECG	Physical illness	snomed
102614006	Generalised abdominal pain	Physical illness	snomed
102616008	Painful mouth	Physical illness	snomed
102617004	Congestion of throat	Physical illness	snomed
102660008	Abnormal glucose level	Physical illness	snomed
102874004	Possible pregnancy	Physical illness	snomed
102878001	Recurrent miscarriage	Physical illness	snomed
102897001	Feeling intoxicated	Alcohol and substance misuse	snomed
102911000	Thoughts of self harm	Suicidal behaviours and self−harm	snomed
102920009	Fear of contracting HIV infection	Physical illness	snomed
10298002	Victim, motorcycle rider in vehicular AND/OR traffic accident	Accident and injury	snomed
103322003	Request by third party on behalf of patient	Other	snomed
10351008	Suppurative tonsillitis	Physical illness	snomed
103552005	Cyst form of protozoa	Physical illness	snomed
103695000	Preparation of medical certificate	Other	snomed
10380004	Crushing injury of finger	Accident and injury	snomed
10385009	Digoxin poisoning	Suicidal behaviours and self−harm	snomed
10509002	Acute bronchitis	Physical illness	snomed
105481005	Refusing food	Physical illness	snomed
105485001	Family tension	Mental health	snomed
105549004	Abuses volatile solvents	Alcohol and substance misuse	snomed
105593004	Electrolyte imbalance	Physical illness	snomed

ed_code	description	presentation_category	classification
105606008	Injury of musculoskeletal system	Accident and injury	snomed
105629000	Chlamydial infection	Physical illness	snomed
10573002	Amnionitis	Physical illness	snomed
105995000	Disorder of teeth AND/OR supporting structures	Physical illness	snomed
106004004	Haemorrhagic complication of pregnancy	Physical illness	snomed
10601006	Pain in lower limb	Physical illness	snomed
106126000	Emotional state finding	Mental health	snomed
106143002	Sexuality related problem	Mental health	snomed
106190000	Allergic state	Physical illness	snomed
10633002	Acute congestive heart failure	Physical illness	snomed
10679007	Infection caused by Giardia lamblia	Physical illness	snomed
10743008	Irritable bowel syndrome	Physical illness	snomed
108365000	Infection of skin	Physical illness	snomed
108367008	Dislocation of joint	Accident and injury	snomed
1085006	Vulval candidiasis	Physical illness	snomed
10890000	Disorder of salivary gland	Physical illness	snomed
109245003	Periorbital cellulitis	Physical illness	snomed
109248001	Inflammation related to voluntary body piercing	Accident and injury	snomed
109256003	Mucositis following chemotherapy	Physical illness	snomed
109363001	Right pneumothorax	Physical illness	snomed
109779003	Abscess of buccal space of mouth	Physical illness	snomed
110015006	Injury of penis	Accident and injury	snomed
110030002	Concussion injury of brain	Accident and injury	snomed
110067005	Simple laceration of scalp	Accident and injury	snomed
110077007	Simple laceration of chin	Accident and injury	snomed
110084004	Simple laceration of tongue	Accident and injury	snomed
110151001	Superficial injury of mouth	Accident and injury	snomed
110168002	Abrasion of chin	Accident and injury	snomed
110244000	Contusion of forehead	Accident and injury	snomed
110265006	Postoperative haemorrhage	Physical illness	snomed
110286006	Self abuse	Suicidal behaviours and self−harm	snomed
110292000	Difficulty eating	Physical illness	snomed
110399007	Simple laceration	Accident and injury	snomed
11092001	Sinus tachycardia	Physical illness	snomed
110979008	Irritant contact dermatitis	Physical illness	snomed

ed_code	description	presentation_category	classification
111183001	Abscess of toe	Physical illness	snomed
111189002	Acute contact dermatitis	Physical illness	snomed
111224002	Derangement of meniscus	Physical illness	snomed
111243002	Bursitis of knee	Physical illness	snomed
111275004	Abscess of nasal septum	Physical illness	snomed
111350000	Acute gastric erosion associated with drug ingestion	Physical illness	snomed
111423006	Retained products of conception following abortion	Physical illness	snomed
111479008	Organic mental disorder	Mental health	snomed
111483008	Catatonic schizophrenia in remission	Mental health	snomed
111484002	Undifferentiated schizophrenia	Mental health	snomed
111520007	Ring corneal ulcer	Physical illness	snomed
111533001	Vertical nystagmus	Physical illness	snomed
111541001	Benign paroxysmal positional vertigo	Physical illness	snomed
111556005	Diabetic ketoacidosis without coma	Physical illness	snomed
11157007	Ventricular bigeminy	Physical illness	snomed
111640008	Closed fracture of radius	Accident and injury	snomed
111643005	Open fracture of patella	Accident and injury	snomed
111746009	Mechanical complication of device	Physical illness	snomed
111843007	Viral gastroenteritis	Physical illness	snomed
11196001	Poisoning by opiate AND/OR related narcotic	Alcohol and substance misuse	snomed
111984006	Ingestion, function (observable entity)	Accident and injury	snomed
111985007	Chronic abdominal pain	Physical illness	snomed
112082005	Inappropriate behaviour	Mental health	snomed
112101004	Dental headache	Physical illness	snomed
112222000	Raised intraocular pressure	Physical illness	snomed
112625008	Cutaneous eruption	Physical illness	snomed
113164004	Childhood or adolescent antisocial behaviour	Mental health	snomed
11387009	Psychoactive substance−induced organic mental disorder	Mental health	snomed
11437003	Contusion of back	Accident and injury	snomed
116224001	Complication of procedure	Physical illness	snomed
116289008	Abdominal bloating	Physical illness	snomed
116290004	Acute abdominal pain	Physical illness	snomed
11639007	Puncture	Accident and injury	snomed
11687002	Gestational diabetes mellitus	Physical illness	snomed
118185001	Pregnancy observations	Physical illness	snomed

ed_code	description	presentation_category	classification
118213005	Postpartum finding	Physical illness	snomed
118216002	Labour observations	Physical illness	snomed
11833005	Dry cough	Physical illness	snomed
11840006	Traveller's diarrhoea	Physical illness	snomed
118938008	Disease of mouth	Physical illness	snomed
118940003	Neurological disorder	Physical illness	snomed
118944007	Disorder of shoulder	Physical illness	snomed
119416008	Epigastric discomfort	Physical illness	snomed
119420007	Haemorrhagic ovarian cyst	Physical illness	snomed
11980003	Burn of foot	Accident and injury	snomed
12063002	Rectal haemorrhage	Physical illness	snomed
12184005	Visual field defect	Physical illness	snomed
12204004	Closed fracture of multiple ribs	Accident and injury	snomed
12295008	Bronchiectasis	Physical illness	snomed
123971006	Colles' fracture	Accident and injury	snomed
123972004	Reversed Colles' fracture	Accident and injury	snomed
123973009	Monteggia's fracture	Accident and injury	snomed
12402003	Scar	Other	snomed
12441001	Nosebleed	Physical illness	snomed
12463005	Infectious gastroenteritis	Physical illness	snomed
125593007	Injury of face	Accident and injury	snomed
125594001	Injury of shoulder region	Accident and injury	snomed
125595000	Injury of upper arm	Accident and injury	snomed
125596004	Injury of elbow	Accident and injury	snomed
125597008	Injury of forearm	Accident and injury	snomed
125598003	Injury of wrist	Accident and injury	snomed
125599006	Injury of hand	Accident and injury	snomed
125600009	Injury of hip region	Accident and injury	snomed
125601008	Injury of knee	Accident and injury	snomed
125602001	Injury of lower leg	Accident and injury	snomed
125603006	Injury of ankle	Accident and injury	snomed
125604000	Injury of foot	Accident and injury	snomed
125605004	Fracture of bone	Accident and injury	snomed
125606003	Fracture of cervical spine	Accident and injury	snomed
125607007	Fracture of thoracic spine	Accident and injury	snomed

ed_code	description	presentation_category	classification
125608002	Fracture of lumbar spine	Accident and injury	snomed
125614009	Closed traumatic dislocation of glenohumeral joint	Accident and injury	snomed
125615005	Traumatic dislocation of shoulder region	Accident and injury	snomed
125617002	Traumatic dislocation of elbow joint	Accident and injury	snomed
125619004	Traumatic dislocation of joint of finger	Accident and injury	snomed
125620005	Traumatic dislocation of joint of thumb	Accident and injury	snomed
125621009	Traumatic dislocation of hip joint	Accident and injury	snomed
125622002	Traumatic dislocation of ankle joint	Accident and injury	snomed
125623007	Traumatic dislocation of joint of foot	Accident and injury	snomed
125643001	Open wound	Accident and injury	snomed
125644007	Open wound of neck	Accident and injury	snomed
125647000	Open wound of axillary region	Accident and injury	snomed
125650002	Open wound of elbow	Accident and injury	snomed
125652005	Open wound of hand	Accident and injury	snomed
125653000	Open wound of finger	Accident and injury	snomed
125659001	Open wound of thigh	Accident and injury	snomed
125660006	Open wound of knee	Accident and injury	snomed
125661005	Open wound of lower leg	Accident and injury	snomed
125663008	Open wound of foot	Accident and injury	snomed
125665001	Crushing injury	Accident and injury	snomed
125666000	Burn	Accident and injury	snomed
125667009	Contusion	Accident and injury	snomed
125668004	Contusion of face	Accident and injury	snomed
125670008	Foreign body	Accident and injury	snomed
125802004	Closed traumatic dislocation of interphalangeal joint of finger	Accident and injury	snomed
125871005	Fracture of coccyx	Accident and injury	snomed
1261007	Multiple fractures of ribs	Accident and injury	snomed
126485001	Urticaria	Physical illness	snomed
12676007	Fracture of radius	Accident and injury	snomed
127034005	Pancytopenia	Physical illness	snomed
127086001	Cervical lymphadenopathy	Physical illness	snomed
127158006	Pelvic lymphadenopathy	Physical illness	snomed
127277000	Injury of female genital system	Accident and injury	snomed
127278005	Injury of upper limb	Accident and injury	snomed
127279002	Injury of lower limb	Accident and injury	snomed

ed_code	description	presentation_category	classification
127281000	Fracture of occipital condyle	Accident and injury	snomed
127314000	Open wound of chest wall	Accident and injury	snomed
127348004	Motor vehicle accident victim	Accident and injury	snomed
127349007	Motor vehicle accident, driver	Accident and injury	snomed
127350007	Motor vehicle accident, passenger	Accident and injury	snomed
127364007	Primip	Physical illness	snomed
127365008	Gravida 2	Physical illness	snomed
127785005	Active or passive immunisation	Other	snomed
128045006	Cellulitis	Physical illness	snomed
128053003	Deep venous thrombosis	Physical illness	snomed
128069005	Injury of abdomen	Accident and injury	snomed
128200000	Complex regional pain syndrome	Physical illness	snomed
128241005	Inflammatory disease of liver	Physical illness	snomed
128276007	Cellulitis of foot	Physical illness	snomed
128293007	Chronic mental disorder	Mental health	snomed
128295000	Inflammatory disorder of the eye	Physical illness	snomed
128302006	Chronic hepatitis C	Physical illness	snomed
128351009	Eye infection	Physical illness	snomed
128462008	Secondary malignant neoplastic disease	Physical illness	snomed
128473001	Uveitis	Physical illness	snomed
128477000	Abscess	Physical illness	snomed
128600008	Acute ulcerative colitis	Physical illness	snomed
128606002	Disorder of the urinary system	Physical illness	snomed
128613002	Seizure disorder	Physical illness	snomed
128869009	Scabies	Physical illness	snomed
128870005	Crusted scabies	Physical illness	snomed
129036004	Medication dependent	Other	snomed
129103003	Endometriosis	Physical illness	snomed
129127001	Ear infection	Physical illness	snomed
129135003	Injury of nerve of upper limb	Accident and injury	snomed
12939007	Chronic disorganised schizophrenia	Mental health	snomed
129846003	Polypharmacy	Other	snomed
130951007	Urinary retention	Physical illness	snomed
130968006	Self−mutilation	Suicidal behaviours and self−harm	snomed
130987000	Acute confusion	Physical illness	snomed

ed_code	description	presentation_category	classification
131138007	Abnormal serotonin	Alcohol and substance misuse	snomed
131148009	Bleeding	Physical illness	snomed
13164000	Reduced visual acuity	Physical illness	snomed
13180005	Retrograde menstruation	Physical illness	snomed
13197004	Contraception	Physical illness	snomed
13200003	Peptic ulcer	Physical illness	snomed
13212004	Partial thickness burn of multiple sites	Accident and injury	snomed
13301002	Cellulitis of knee	Physical illness	snomed
134187008	Child protection procedure (regime/therapy)	Other	snomed
134222005	Penetrating wound (morphologic abnormality)	Accident and injury	snomed
134407002	Chronic back pain	Physical illness	snomed
13445001	Ménière's disease	Physical illness	snomed
13457005	Foreign body in anus	Accident and injury	snomed
135869000	Torn frenulum of penis	Accident and injury	snomed
135883003	Cough with fever	Physical illness	snomed
13595002	Torsion of ovary	Physical illness	snomed
13600006	Folliculitis	Physical illness	snomed
13645005	COPD	Physical illness	snomed
13680009	Cellulitis of forearm	Physical illness	snomed
13746004	Bipolar disorder	Mental health	snomed
13758004	Paraphimosis	Physical illness	snomed
13791008	Asthenia	Physical illness	snomed
13802001	Abscess of axilla	Physical illness	snomed
13924000	Wound (morphologic abnormality)	Accident and injury	snomed
13973009	Grand mal status	Physical illness	snomed
14094001	Hyperemesis gravidarum	Physical illness	snomed
14140009	Hyperkalaemia	Physical illness	snomed
1415005	Lymphangitis	Physical illness	snomed
14189004	Measles	Physical illness	snomed
14261008	Burn of upper arm	Accident and injury	snomed
14276000	Burn any degree involving 10−19 percent of body surface	Accident and injury	snomed
14380007	Foreign body in mouth	Accident and injury	snomed
14448006	Swallowed foreign body	Accident and injury	snomed
14540002	Compression of vein	Physical illness	snomed
14664006	Epidermal burn of hand	Accident and injury	snomed

ed_code	description	presentation_category	classification
14669001	Acute kidney injury	Physical illness	snomed
1475003	Cold sore	Physical illness	snomed
14756005	Parotitis	Physical illness	snomed
14760008	Constipation	Physical illness	snomed
14812002	Cellulitis of penis	Physical illness	snomed
1482004	Chalazion	Physical illness	snomed
1489008	Stye	Physical illness	snomed
14893008	Burn of hand	Accident and injury	snomed
14992004	Contusion of penis	Accident and injury	snomed
15033003	Peritonsillar abscess	Physical illness	snomed
15167005	Alcohol abuse	Alcohol and substance misuse	snomed
15188001	Hearing loss	Physical illness	snomed
15203004	Sudden visual loss	Physical illness	snomed
15220000	Laboratory test panel	Other	snomed
15250008	Disorder of cornea	Physical illness	snomed
15387003	Being sick	Physical illness	snomed
1539003	Trigger finger	Physical illness	snomed
15550007	Open wound of wrist with tendon involvement	Accident and injury	snomed
15555002	Acute pericarditis	Physical illness	snomed
15574005	Fracture of foot	Accident and injury	snomed
15628003	Gonorrhoea	Physical illness	snomed
15771004	Diabetes insipidus	Physical illness	snomed
15802004	Dystonia	Physical illness	snomed
15805002	Acute sinusitis	Physical illness	snomed
158094009	Non−accidental injury to child	Accident and injury	snomed
16001004	Otalgia	Physical illness	snomed
16047007	Benzodiazepine (product)	Alcohol and substance misuse	snomed
160476009	Personal details	Other	snomed
160573003	AI − Alcohol intake	Alcohol and substance misuse	snomed
160592001	Alcohol intake above recommended sensible limits	Alcohol and substance misuse	snomed
160693001	Poor mobility	Physical illness	snomed
160823009	Boyfriend relationship problem	Mental health	snomed
16096002	Chemical burn of eyelid AND/OR periocular area (disorder)	Accident and injury	snomed
161051006	At risk violence in the home (finding)	Other	snomed
161128009	Behavioural problems at school	Mental health	snomed

ed_code	description	presentation_category	classification
16114001	Fracture of ankle	Accident and injury	snomed
161152002	Social problem	Other	snomed
16119006	Abscess of jaw	Physical illness	snomed
161424002	History of − viral illness (context−dependent category)	Physical illness	snomed
161469008	Has had depression	Mental health	snomed
161512007	History of pulmonary embolus	Physical illness	snomed
161538007	H/O: haematemesis	Physical illness	snomed
161882006	Stiff neck	Physical illness	snomed
161891005	Backache	Physical illness	snomed
161898004	Falls	Accident and injury	snomed
161920001	Respiratory symptom	Physical illness	snomed
161941007	Dyspnoea at rest	Physical illness	snomed
161969004	Fluttering heart	Physical illness	snomed
161972006	Central chest pain	Physical illness	snomed
161973001	Anterior chest wall pain	Physical illness	snomed
162031009	Indigestion	Physical illness	snomed
162042000	Abdominal wall pain	Physical illness	snomed
162046002	Central abdominal pain	Physical illness	snomed
162049009	Left flank pain	Physical illness	snomed
162050009	Right flank pain	Physical illness	snomed
162051008	Right iliac fossa pain	Physical illness	snomed
162052001	Left iliac fossa pain	Physical illness	snomed
162053006	Suprapubic pain	Physical illness	snomed
162116003	Frequent urination	Physical illness	snomed
162128006	Poor stream of urine	Physical illness	snomed
162145001	Vulval pain	Physical illness	snomed
162147009	C/O pelvic pain	Physical illness	snomed
162169002	Sexual activity (finding)	Physical illness	snomed
162181003	Vaginal discomfort	Physical illness	snomed
162218007	Stress−related problem	Mental health	snomed
162249002	Has tingling sensation (finding)	Physical illness	snomed
162277006	Sees flashes	Physical illness	snomed
162278001	Floaters in visual field	Physical illness	snomed
162279009	Temporary visual disturbance	Physical illness	snomed
162290004	Dry eyes	Physical illness	snomed

ed_code	description	presentation_category	classification
162299003	Generalised headache	Physical illness	snomed
162307009	Aching headache	Physical illness	snomed
162314006	Harmful thoughts	Suicidal behaviours and self−harm	snomed
162318009	Life crisis	Mental health	snomed
162359003	Bilateral earache	Physical illness	snomed
162373007	Has nosebleeds − epistaxis (disorder)	Physical illness	snomed
162388002	Has a sore throat	Physical illness	snomed
162397003	Pain in throat	Physical illness	snomed
162400007	Throat irritation	Physical illness	snomed
162410003	C/O: a swelling	Physical illness	snomed
162415008	C/O: a rash	Physical illness	snomed
162685008	O/E − dehydrated	Physical illness	snomed
16269008	Neuralgia	Physical illness	snomed
162702000	O/E − mentally confused	Mental health	snomed
162713002	O/E − collapse − syncope	Physical illness	snomed
16277007	Collapse (morphologic abnormality)	Physical illness	snomed
162832003	O/E − submandibular swelling	Physical illness	snomed
16292008	Cannabis sativa poisoning	Alcohol and substance misuse	snomed
162984005	CVS examined − NAD	Physical illness	snomed
163140002	O/E − lip swelling	Physical illness	snomed
16320008	Mixed feelings	Mental health	snomed
163220003	On examination − abdominal pain − right iliac	Physical illness	snomed
16331000	Heartburn	Physical illness	snomed
163378005	O/E − testicular swelling	Physical illness	snomed
163588007	O/E − fit/convulsion	Physical illness	snomed
1639007	Abnormal pelvic organ affecting pregnancy	Physical illness	snomed
164033006	On examination − fluorescein−corneal abrasion	Physical illness	snomed
164192001	O/E − foreign body in nose	Accident and injury	snomed
164644002	On examination − sign in skin (context−dependent category)	Physical illness	snomed
164846002	ECG requested (context−dependent category)	Physical illness	snomed
165232002	Urinary incontinence	Physical illness	snomed
165309004	Behavior assessment (procedure)	Mental health	snomed
165311008	Mildly abnormal behaviour	Mental health	snomed
165517008	Neutrophil count below reference range	Physical illness	snomed
165581004	Internationalized ratio	Other	snomed

ed_code	description	presentation_category	classification
165583001	INR − international normal ratio abnormal	Physical illness	snomed
1658003	Closed fracture of acromial end of clavicle	Accident and injury	snomed
16607004	Missed miscarriage	Physical illness	snomed
16631009	Transverse myelitis	Physical illness	snomed
166318006	Blood chemistry abnormal	Physical illness	snomed
166690008	Low serum potassium level	Physical illness	snomed
16698000	Acute abscess of breast	Physical illness	snomed
167235001	Urine: red − blood	Physical illness	snomed
16761005	Oesophagitis	Physical illness	snomed
16838000	Mediastinal emphysema	Physical illness	snomed
168393006	General pathology	Physical illness	snomed
168501001	Radiology result abnormal	Other	snomed
16863000	Incomplete miscarriage	Physical illness	snomed
168646009	Plain X−ray scaphoid abnormal	Physical illness	snomed
168776009	Soft tissue limb X−ray normal (finding)	Physical illness	snomed
169254007	Ultrasound scan normal (finding)	Other	snomed
16932000	Nausea and vomiting	Physical illness	snomed
169686005	Alpha−fetoprotein blood test wanted (finding)	Physical illness	snomed
170473002	Meningococcus contact (finding)	Physical illness	snomed
170537006	Requires a tetanus booster	Other	snomed
17059001	Prepatellar bursitis	Physical illness	snomed
170681006	Psychiatric drug side effects	Mental health	snomed
170917004	Warfarin side effects	Physical illness	snomed
171102007	Screening offered (finding)	Other	snomed
171112000	Screening due (finding)	Other	snomed
171251002	Urine screening abnormal	Physical illness	snomed
171279008	Vaccination due	Other	snomed
17133001	Abscess of submandibular region	Physical illness	snomed
171423009	Mental Health Act examination	Mental health	snomed
171431004	Patient reviewed at hospital (procedure)	Other	snomed
17204006	Pneumoperitoneum	Physical illness	snomed
17222009	Closed fracture of distal end of radius	Accident and injury	snomed
17226007	Adjustment disorder	Mental health	snomed
17326005	Normal mental state	Mental health	snomed
17329003	Ureteric colic	Physical illness	snomed

ed_code	description	presentation_category	classification
173365006	Removal of foreign body from tongue (procedure)	Accident and injury	snomed
17338001	Ventricular premature beats	Physical illness	snomed
17354003	Abscess of shoulder	Physical illness	snomed
17369002	Miscarriage	Physical illness	snomed
17383000	Carbon monoxide poisoning	Accident and injury	snomed
17741008	Acute tonsillitis	Physical illness	snomed
178155009	Plastic repair of tendon (procedure)	Accident and injury	snomed
17883008	Sprain of hip	Accident and injury	snomed
180289009	Application of plaster cast	Other	snomed
18070006	Impacted cerumen	Physical illness	snomed
18117002	Paranoid personality trait	Mental health	snomed
18165001	Jaundice	Physical illness	snomed
18171007	Fracture of phalanx of finger	Accident and injury	snomed
182446003	MM − Medial meniscus	Accident and injury	snomed
182531007	Dressing of wound	Other	snomed
182782007	Dermatitis (disorder)	Physical illness	snomed
182817000	Drug prescription	Other	snomed
182832007	Management of drug regimen	Other	snomed
182836005	Review of medicine	Other	snomed
182850000	Drugs not issued (finding)	Other	snomed
182885000	Medication satisfactory (finding)	Other	snomed
182893000	Forgot to bring medication (finding)	Other	snomed
183425000	Social care	Other	snomed
183455007	Psychiatric emergency hospital admission (procedure)	Mental health	snomed
18347007	Spinal stenosis of lumbar region	Physical illness	snomed
183616001	Follow−up arranged (finding)	Other	snomed
183644000	Surgical follow−up	Physical illness	snomed
183653007	Fracture therapy follow−up	Accident and injury	snomed
183654001	Follow−up in outpatient clinic	Other	snomed
183690008	Psychiatric self−referral (procedure)	Mental health	snomed
183767005	Listed for admission to hospital (finding)	Other	snomed
183924009	Referral needed	Other	snomed
184770004	Miscellaneous certificate request	Other	snomed
18504008	Toxic shock syndrome	Physical illness	snomed
185351004	Administrative reason for encounter	Other	snomed

ed_code	description	presentation_category	classification
185383005	Asked to come in − investigation result	Other	snomed
1855002	Learning disability	Other	snomed
18629005	Administration of medicine	Other	snomed
186544000	Dermatitis of eyelid caused by herpes simplex virus	Physical illness	snomed
186679007	Conjunctivitis caused by adenovirus	Physical illness	snomed
186738001	Epidemic vertigo	Physical illness	snomed
186846005	Early symptomatic syphilis	Physical illness	snomed
18773000	Cyclical vomiting syndrome	Physical illness	snomed
188463006	Chlamydial pelvic inflammatory disease	Physical illness	snomed
18876004	Pain in finger	Physical illness	snomed
18949003	Change of dressing	Other	snomed
18963009	Mood swings	Mental health	snomed
190902006	Fluid imbalance	Physical illness	snomed
190905008	Cystic fibrosis	Physical illness	snomed
1912002	Fall	Accident and injury	snomed
19130008	Traumatic abnormality	Accident and injury	snomed
191306005	Henoch−Schönlein purpura	Physical illness	snomed
191357009	Eosinophilic disorder	Physical illness	snomed
191480000	Alcohol withdrawal syndrome	Alcohol and substance misuse	snomed
191483003	Drug−induced psychosis	Mental health	snomed
191525009	Non−organic psychosis	Mental health	snomed
191531007	Acute exacerbation of chronic schizophrenia	Mental health	snomed
191542003	Catatonic schizophrenia	Mental health	snomed
19155002	Dysfunctional uterine bleeding	Physical illness	snomed
191555002	Acute exacerbation of chronic paranoid schizophrenia	Mental health	snomed
191572009	Acute exacerbation of chronic schizoaffective schizophrenia	Mental health	snomed
191616006	Recurrent depression	Mental health	snomed
191618007	Bipolar affective disorder, current episode manic	Mental health	snomed
191623007	Bipolar affective disorder, currently manic, severe, with psychosis	Mental health	snomed
191627008	Bipolar affective disorder, current episode depression	Mental health	snomed
191629006	Bipolar affective disorder, currently depressed, mild	Mental health	snomed
191632009	Bipolar affective disorder, currently depressed, severe, with psychosis	Mental health	snomed
191667009	Paranoid disorder	Mental health	snomed
191677006	Acute hysterical psychosis	Mental health	snomed
19169002	Miscarriage in first trimester	Physical illness	snomed

ed_code	description	presentation_category	classification
191697001	Borderline psychosis of childhood	Mental health	snomed
191714002	Dissociative convulsions	Mental health	snomed
191723004	Agoraphobia without mention of panic attacks	Mental health	snomed
191736004	Obsessive−compulsive disorder	Mental health	snomed
191765005	Emotionally unstable personality disorder	Mental health	snomed
191802004	Acute alcoholic intoxication in alcoholism	Alcohol and substance misuse	snomed
191816009	Drug dependence	Alcohol and substance misuse	snomed
191956005	Psychogenic hyperventilation	Mental health	snomed
192041001	Acute situational disturbance	Suicidal behaviours and self−harm	snomed
192080009	Chronic depression	Mental health	snomed
192085004	Anger reaction	Mental health	snomed
19213003	Infectious diarrhoeal disease	Physical illness	snomed
19227008	FB − Foreign body	Accident and injury	snomed
192362008	Bipolar affective disorder, current episode mixed	Mental health	snomed
192454004	Nonorganic insomnia	Mental health	snomed
19259001	Closed fracture of proximal radius and ulna	Accident and injury	snomed
192644005	Meningococcal meningitis	Physical illness	snomed
19284003	Abscess of back, except buttock	Physical illness	snomed
192970008	Cauda equina syndrome	Physical illness	snomed
193000002	Temporal lobe epilepsy	Physical illness	snomed
193031009	Cluster headache	Physical illness	snomed
193039006	Complicated migraine	Physical illness	snomed
193093009	Bell's palsy	Physical illness	snomed
193462001	Insomnia	Mental health	snomed
193531003	Borderline glaucoma	Physical illness	snomed
193722001	Low vision, both eyes	Physical illness	snomed
193728002	Better eye: moderate visual impairment, Lesser eye: moderate visual impairment	Physical illness	snomed
193783008	Interstitial and deep keratitis	Physical illness	snomed
193967004	Swelling of eyelid	Physical illness	snomed
193982009	Epiphora	Physical illness	snomed
194005002	Orbital cellulitis	Physical illness	snomed
194281003	Acute suppurative otitis media	Physical illness	snomed
194288009	Acute left otitis media	Physical illness	snomed
194289001	Acute right otitis media	Physical illness	snomed
194290005	Acute bilateral otitis media	Physical illness	snomed

ed_code	description	presentation_category	classification
19471005	Lymphadenitis	Physical illness	snomed
194828000	Angina	Physical illness	snomed
19491003	Injury of nose	Accident and injury	snomed
19496008	Foreign body in larynx	Accident and injury	snomed
195453000	Internal hemorrhoids, simple	Physical illness	snomed
19551004	Human herpesvirus 3	Physical illness	snomed
195647007	Acute respiratory infections	Physical illness	snomed
195658003	Acute bacterial pharyngitis	Physical illness	snomed
195662009	Acute viral pharyngitis	Physical illness	snomed
195667003	Acute follicular tonsillitis	Physical illness	snomed
195669000	Acute catarrhal tonsillitis	Physical illness	snomed
195671000	Acute bacterial tonsillitis	Physical illness	snomed
195676005	Acute viral tonsillitis	Physical illness	snomed
19569008	Mild hyperemesis gravidarum	Physical illness	snomed
195742007	Acute lower respiratory tract infection	Physical illness	snomed
195967001	Asthma	Physical illness	snomed
196075003	Pleurisy	Physical illness	snomed
196417006	Impacted third molar tooth	Physical illness	snomed
19660004	Disorder of soft tissue	Physical illness	snomed
19674006	Immersion	Accident and injury	snomed
196746003	Persistent vomiting	Physical illness	snomed
196754001	Gastric spasm	Physical illness	snomed
196863007	Irreducible umbilical hernia	Physical illness	snomed
196885001	Obstructed epigastric hernia	Physical illness	snomed
197079003	Subacute intestinal obstruction	Physical illness	snomed
197118003	Constipation − functional	Physical illness	snomed
197119006	Acute constipation	Physical illness	snomed
197232005	Anorectal pain	Physical illness	snomed
197456007	Acute pancreatitis	Physical illness	snomed
197480006	Anxiety disorder	Mental health	snomed
197927001	Recurrent urinary tract infection	Physical illness	snomed
197938001	Painless haematuria	Physical illness	snomed
197939009	Painful haematuria	Physical illness	snomed
197941005	Macroscopic haematuria	Physical illness	snomed
197983000	Orchitis and epididymitis	Physical illness	snomed

ed_code	description	presentation_category	classification
198035003	Oedema of penis	Physical illness	snomed
198108005	Breast infection	Physical illness	snomed
198130006	Pelvic inflammatory disease	Physical illness	snomed
19824006	Infection of skin and/or subcutaneous tissue	Physical illness	snomed
198820000	Endometritis following abortive pregnancy	Physical illness	snomed
198862007	Readmission for retained products of conception, legal termination of pregnancy	Physical illness	snomed
198881004	Pregnancy complications	Physical illness	snomed
20010003	Borderline personality disorder	Mental health	snomed
200146002	Caesarean delivery − delivered	Physical illness	snomed
20022000	Hemiparesis	Physical illness	snomed
200627004	Multiple boils	Physical illness	snomed
200652002	Cellulitis of face	Physical illness	snomed
200665006	Cellulitis and abscess of arm	Physical illness	snomed
200676005	Cellulitis and abscess of buttock	Physical illness	snomed
200679003	Cellulitis and abscess of thigh	Physical illness	snomed
200680000	Cellulitis and abscess of knee	Physical illness	snomed
200714005	Pilonidal sinus with abscess	Physical illness	snomed
200715006	Pilonidal sinus without abscess	Physical illness	snomed
200751004	Abscess of face	Physical illness	snomed
200755008	Abscess of limb	Physical illness	snomed
200893007	Localised skin eruption caused by drug and medicament	Physical illness	snomed
201060008	Disorder of upper respiratory system	Physical illness	snomed
202214002	Recurrent dislocation of shoulder − inferior	Accident and injury	snomed
202239006	Recurrent subluxation of carpometacarpal joint	Accident and injury	snomed
202246002	Recurrent dislocation of the patellofemoral joint	Accident and injury	snomed
202248001	Recurrent subluxation of the patella	Accident and injury	snomed
202329003	Detachment of the glenoid labrum and/or capsule of the shoulder joint	Accident and injury	snomed
20236002	Labour established	Physical illness	snomed
202373004	Elbow joint effusion	Accident and injury	snomed
202381003	Knee joint effusion	Physical illness	snomed
202472008	Hand joint pain	Physical illness	snomed
202480001	Elbow joint pain	Physical illness	snomed
202482009	Wrist joint pain	Physical illness	snomed
202487003	Sacroiliac joint pain	Physical illness	snomed
202490009	Ankle joint pain	Physical illness	snomed

ed_code	description	presentation_category	classification
20262006	Ataxia	Physical illness	snomed
202757008	Cervical disc disorder with radiculopathy	Physical illness	snomed
202855006	Lateral epicondylitis	Accident and injury	snomed
202882003	Plantar fasciitis	Physical illness	snomed
202942009	Ganglion of wrist	Physical illness	snomed
20301004	Dysphasia	Physical illness	snomed
203556007	Deviation of finger	Accident and injury	snomed
20425006	Labyrinthine disorder	Physical illness	snomed
2043009	Alcoholic gastritis	Alcohol and substance misuse	snomed
20490007	Epidermal burn of lower leg	Accident and injury	snomed
20502007	Pain in scrotum	Physical illness	snomed
20511007	Fracture of hand	Accident and injury	snomed
2055003	Recurrent erosion of cornea	Physical illness	snomed
20607006	Gingivostomatitis	Physical illness	snomed
206422009	Perinatal transient vaginal bleeding	Physical illness	snomed
206573003	Withdrawal symptoms from therapeutic use of drugs in newborn	Physical illness	snomed
2070002	Burning sensation in eye	Accident and injury	snomed
207066005	[D]Cough	Physical illness	snomed
207078009	[D]Chest pain	Physical illness	snomed
207089002	[D]Musculoskeletal chest pain	Physical illness	snomed
207111007	[D]Nausea	Physical illness	snomed
207205003	[D]Abdominal pain	Physical illness	snomed
207222001	[D]Perineal pain	Physical illness	snomed
2073000	Delusions	Mental health	snomed
207753003	Fracture of mandible, closed	Accident and injury	snomed
207755005	Closed fracture of mandible, condylar process	Accident and injury	snomed
207759004	Closed fracture of mandible, angle of jaw	Accident and injury	snomed
207787008	Fracture of orbital floor	Accident and injury	snomed
20824003	Chronic cholecystitis	Physical illness	snomed
208325003	Closed fracture radial styloid	Accident and injury	snomed
208326002	Closed fracture distal radius, intra−articular, die−punch	Accident and injury	snomed
208371005	Closed fracture scaphoid, waist, transverse	Accident and injury	snomed
208388003	Fracture at wrist and/or hand level	Accident and injury	snomed
208390002	Fracture of first metacarpal bone	Accident and injury	snomed
208393000	Fracture of metacarpal bone	Accident and injury	snomed

ed_code	description	presentation_category	classification
208394006	Closed fracture of metacarpal bone	Accident and injury	snomed
208397004	Closed fracture finger metacarpal base	Accident and injury	snomed
208399001	Closed fracture finger metacarpal neck	Accident and injury	snomed
208400008	Closed fracture finger metacarpal head	Accident and injury	snomed
208401007	Closed fracture finger metacarpal	Accident and injury	snomed
208402000	Closed fracture finger metacarpal, multiple	Accident and injury	snomed
208403005	Closed fracture of thumb metacarpal	Accident and injury	snomed
208430000	Closed fracture of one or more phalanges of hand	Accident and injury	snomed
208434009	Closed fracture thumb proximal phalanx	Accident and injury	snomed
208440002	Closed fracture thumb distal phalanx, base	Accident and injury	snomed
208444006	Closed fracture finger proximal phalanx	Accident and injury	snomed
208445007	Closed fracture finger proximal phalanx, base	Accident and injury	snomed
208450001	Closed fracture finger middle phalanx	Accident and injury	snomed
208451002	Closed fracture finger middle phalanx, base	Accident and injury	snomed
208452009	Closed fracture finger middle phalanx, shaft	Accident and injury	snomed
208454005	Closed fracture finger middle phalanx, head	Accident and injury	snomed
208456007	Closed fracture finger distal phalanx, base	Accident and injury	snomed
208629000	Closed fracture of tibia and fibula, shaft	Accident and injury	snomed
208632002	Open fracture of tibia and fibula, shaft	Accident and injury	snomed
208634001	Closed fracture distal tibia	Accident and injury	snomed
208658002	Closed fracture ankle, lateral malleolus, high	Accident and injury	snomed
208686005	Closed fracture talus, body	Accident and injury	snomed
208688006	Closed fracture metatarsal shaft	Accident and injury	snomed
208689003	Closed fracture metatarsal neck	Accident and injury	snomed
208690007	Closed fracture metatarsal head	Accident and injury	snomed
208710000	Closed fracture proximal phalanx, toe	Accident and injury	snomed
208711001	Closed fracture middle phalanx, toe	Accident and injury	snomed
208719004	Fracture of great toe	Accident and injury	snomed
208857009	Closed traumatic dislocation digit	Accident and injury	snomed
208929003	Closed traumatic dislocation of patellofemoral joint	Accident and injury	snomed
208977003	Injury to multiple structures of knee	Accident and injury	snomed
209349006	Closed fracture dislocation of ankle joint	Accident and injury	snomed
209417005	Sprain, shoulder joint, anterior	Accident and injury	snomed
20944008	Congenital postural scoliosis	Physical illness	snomed
20945009	Superficial burn of face AND/OR head	Accident and injury	snomed

ed_code	description	presentation_category	classification
20946005	Fracture, closed	Accident and injury	snomed
209472007	Sprain thumb, metacarpophalangeal joint, ulnar collateral ligament	Accident and injury	snomed
209494004	Sprain wrist extensors	Accident and injury	snomed
209496002	Sprain tendon of thumb	Accident and injury	snomed
209507002	Strain of quadriceps tendon	Accident and injury	snomed
209529003	Sprain of ankle and/or foot	Accident and injury	snomed
209531007	Sprain, ankle joint, medial	Accident and injury	snomed
209532000	Sprain, ankle joint, lateral	Accident and injury	snomed
209557005	Neck sprain	Accident and injury	snomed
209565008	Lumbar sprain	Accident and injury	snomed
209571002	Coccyx sprain	Accident and injury	snomed
209633004	Rupture of ankle ligament	Accident and injury	snomed
209634005	Rupture of medial ankle ligament	Accident and injury	snomed
209784008	Rupture of flexor tendon of foot	Accident and injury	snomed
209987007	Traumatic subdural haemorrhage	Accident and injury	snomed
21005005	Burning epigastric pain	Accident and injury	snomed
210339009	Open wound of face	Accident and injury	snomed
210342003	Open wound of forehead	Accident and injury	snomed
21055002	Crisis intervention with medication	Mental health	snomed
210566005	Open wound of hand with tendon involvement	Accident and injury	snomed
210579001	Open wound, finger, multiple	Accident and injury	snomed
210580003	Open wound of finger or thumb with complication	Accident and injury	snomed
210617003	Traumatic amputation, thumb tip	Accident and injury	snomed
210711006	Open wound foot, plantar	Accident and injury	snomed
210713009	Open wound, heel	Accident and injury	snomed
210715002	Open wound of foot with tendon involvement	Accident and injury	snomed
21092002	Abscess of finger	Physical illness	snomed
210987008	Abrasion of face	Accident and injury	snomed
211024005	Multiple superficial injuries of head	Accident and injury	snomed
211039006	Abrasion, chest wall	Accident and injury	snomed
21117005	Conjunctival haemorrhage	Physical illness	snomed
211186008	Superficial injury of shoulder and upper arm	Accident and injury	snomed
211189001	Abrasion, shoulder area	Accident and injury	snomed
211199006	Abrasion of axilla, infected	Accident and injury	snomed
211251003	Abrasion, elbow area	Accident and injury	snomed

ed_code	description	presentation_category	classification
211282004	Foreign body in arm	Accident and injury	snomed
211304002	Abrasion hand, palm	Accident and injury	snomed
211311003	Foreign body in hand	Accident and injury	snomed
211312005	Splinter of hand, without major open wound, infected	Accident and injury	snomed
211317004	Abrasion of finger	Accident and injury	snomed
211319001	Abrasion, finger, multiple	Accident and injury	snomed
211332006	Abrasion, knee	Accident and injury	snomed
211402004	Abrasion of foot	Accident and injury	snomed
211463006	Foreign body in skin wound	Accident and injury	snomed
211468002	Superficial injuries involving multiple body regions	Accident and injury	snomed
211506004	Contusion wrist or hand	Accident and injury	snomed
211569008	Crush injury wrist and/or hand	Accident and injury	snomed
211582000	Closed crush injury, finger	Accident and injury	snomed
211583005	Closed crush injury, thumb	Accident and injury	snomed
211604005	Closed crush injury, foot	Accident and injury	snomed
211661000	Alkaline chemical burn of cornea and conjunctival sac	Accident and injury	snomed
211817005	Burn of arm (excluding wrist and hand)	Accident and injury	snomed
211828009	Superficial burn of arm	Accident and injury	snomed
211879003	Burn of wrist(s) and hand(s)	Accident and injury	snomed
211894004	Superficial burn of more than one finger	Accident and injury	snomed
212049006	Burns classified according to percentage of body surface involved	Accident and injury	snomed
212269009	Injury of ulnar nerve at wrist and hand level	Accident and injury	snomed
212279006	Closed injury, digital nerve in finger	Accident and injury	snomed
212282001	Open injury, digital nerve in finger	Accident and injury	snomed
212382003	Compartment syndrome of hand	Physical illness	snomed
212458009	Injury of muscle and tendon at forearm level	Accident and injury	snomed
212459001	Injury of flexor muscle and tendon of thumb at forearm level	Accident and injury	snomed
212462003	Injury of extensor or abductor muscles and tendons of thumb at forearm level	Accident and injury	snomed
212467009	Injury of muscle and tendon at hip and thigh level	Accident and injury	snomed
212472000	Injury of muscle(s) and tendon(s) of peroneal muscle group at lower leg level	Accident and injury	snomed
212491000	Multiple open wounds of lower leg	Accident and injury	snomed
212962007	Drowning and non−fatal immersion	Accident and injury	snomed
212985008	Effects of high−pressure fluids	Other	snomed
213017001	Sexual abuse	Accident and injury	snomed
213024000	Allergic reaction to insect bite	Physical illness	snomed

ed_code	description	presentation_category	classification
213050001	Mechanical complication of Hickman Line	Physical illness	snomed
213150003	Kidney transplant failure and rejection	Physical illness	snomed
213201002	Hyperstimulation of ovaries	Physical illness	snomed
213257006	Generally unwell	Physical illness	snomed
213262007	Postoperative haematoma formation	Physical illness	snomed
213282006	Foreign body left in wound	Accident and injury	snomed
213299007	Postoperative pain	Physical illness	snomed
21333004	Derangement of lateral meniscus	Physical illness	snomed
213386007	Injuries of muscles and tendons involving multiple body regions	Accident and injury	snomed
21351003	Fracture of phalanx of foot	Accident and injury	snomed
214031005	Motor vehicle traffic accident	Accident and injury	snomed
214264003	Lethargy	Physical illness	snomed
21454007	Subarachnoid haemorrhage	Physical illness	snomed
21522001	Abdominal pain	Physical illness	snomed
216004	Delusion of persecution	Mental health	snomed
21626009	Skin hypersensitivity	Physical illness	snomed
21639008	Hypervolaemia	Physical illness	snomed
21647008	Amphetamine dependence	Alcohol and substance misuse	snomed
216530001	Accidental poisoning by benzodiazepine−based tranquilliser	Accident and injury	snomed
216679000	Accidental petrol poisoning	Accident and injury	snomed
21698002	Open fracture of phalanx of finger	Accident and injury	snomed
217082002	Accidental fall	Accident and injury	snomed
217658008	Entire ball of foot	Accident and injury	snomed
217665000	Venomous spider poisoning	Accident and injury	snomed
217697000	Dog bite	Accident and injury	snomed
217701002	Cat bite	Accident and injury	snomed
217706007	Bite of nonvenomous arthropod	Accident and injury	snomed
217835008	Foreign body accidentally entering eye and adnexa	Accident and injury	snomed
21867001	Fracture of upper end of fibula	Accident and injury	snomed
218791008	Adverse reaction to cannabis	Alcohol and substance misuse	snomed
21897009	Generalised anxiety disorder	Mental health	snomed
219006	Current drinker	Alcohol and substance misuse	snomed
219125007	Suicide and selfinflicted poisoning by gases in domestic use	Suicidal behaviours and self−harm	snomed
219152002	Self inflicted lacerations to wrist	Suicidal behaviours and self−harm	snomed
219346009	Injury of unknown intent due to fall from height	Accident and injury	snomed

ed_code	description	presentation_category	classification
21954000	Herpes zoster auricularis	Physical illness	snomed
221360009	Spasticity	Physical illness	snomed
22247000	Dehiscence of surgical wound	Physical illness	snomed
22253000	Pain	Physical illness	snomed
22325002	Abnormal gait	Physical illness	snomed
22343003	Pneumonitis due to fumes	Accident and injury	snomed
2237002	Pleuritic pain	Physical illness	snomed
22399000	Puerperal endometritis	Physical illness	snomed
224338007	In police custody	Other	snomed
22490002	Bleeding from mouth	Physical illness	snomed
224964008	No injuries apparent	Other	snomed
224977004	Feeling upset	Mental health	snomed
225009008	Feeling lost	Mental health	snomed
225049000	Hanging self	Suicidal behaviours and self−harm	snomed
225216003	Acknowledging anxiety	Mental health	snomed
225303001	Evaluating interventions	Other	snomed
225358003	Wound care	Accident and injury	snomed
225416000	Checking patient regularly (regime/therapy)	Other	snomed
225444004	Suicide risk	Suicidal behaviours and self−harm	snomed
225450009	Homicidal thoughts	Mental health	snomed
225457007	Feeling suicidal	Suicidal behaviours and self−harm	snomed
225481000	Abusive behaviour	Mental health	snomed
225552003	Wound finding	Accident and injury	snomed
225553008	Wound dehiscence	Accident and injury	snomed
225564006	Pain of nose	Physical illness	snomed
225565007	Perineal pain	Physical illness	snomed
225573003	Nipple painful	Physical illness	snomed
225577002	Stoma observations	Physical illness	snomed
225589000	Chokes when swallowing	Physical illness	snomed
225599005	Social behavior	Mental health	snomed
22562004	Contusion of scalp	Accident and injury	snomed
225624000	Panic attack	Mental health	snomed
225824003	Victim of physical abuse	Accident and injury	snomed
225858006	Safety observations	Mental health	snomed
225911002	Able to cope with pain	Physical illness	snomed

ed_code	description	presentation_category	classification
225928004	Patient self−discharge against medical advice	Other	snomed
225941000	Wears odd shoes	Physical illness	snomed
225956009	At risk of urinary tract infection	Physical illness	snomed
226034001	Injecting drug user	Alcohol and substance misuse	snomed
22631008	Unsteady when walking	Physical illness	snomed
22724000	Injury of urethra	Accident and injury	snomed
228150001	Mental health impairment	Mental health	snomed
228156007	Intellectual functioning disability	Other	snomed
228158008	Walking disability	Accident and injury	snomed
228273003	Finding relating to alcohol drinking behaviour	Alcohol and substance misuse	snomed
228315001	Binge drinker	Alcohol and substance misuse	snomed
228326007	Binge drinking	Alcohol and substance misuse	snomed
228366006	Finding relating to drug misuse behaviour	Alcohol and substance misuse	snomed
228388006	Intravenous drug user	Alcohol and substance misuse	snomed
22878006	Contusion of knee	Accident and injury	snomed
230145002	Difficulty breathing	Physical illness	snomed
230315008	Drug−induced dystonia	Physical illness	snomed
230372003	Acute relapsing multiple sclerosis	Physical illness	snomed
2304001	Discitis	Physical illness	snomed
230434009	Seizure with provoking factor	Physical illness	snomed
230461009	Headache disorder	Physical illness	snomed
230471006	Chronic tension−type headache	Physical illness	snomed
23056005	Sciatica	Physical illness	snomed
230619007	Contusion of peripheral nerve	Accident and injury	snomed
23065003	Stenosis of intestine	Physical illness	snomed
230654000	Painful legs and moving toes	Physical illness	snomed
230690007	Stroke	Physical illness	snomed
230808006	Brain ventricular shunt obstruction	Physical illness	snomed
231466009	Acute drug intoxication	Alcohol and substance misuse	snomed
231473004	Benzodiazepine dependence	Alcohol and substance misuse	snomed
231474005	Diazepam dependence	Alcohol and substance misuse	snomed
231477003	Heroin dependence	Alcohol and substance misuse	snomed
231485007	Post−schizophrenic depression	Mental health	snomed
231489001	Acute transient psychotic disorder	Mental health	snomed
231494001	Mania	Mental health	snomed

ed_code	description	presentation_category	classification
231496004	Hypomania	Mental health	snomed
231502005	Situational panic attack	Mental health	snomed
231504006	Mixed anxiety and depressive disorder	Mental health	snomed
231520001	Behavioural syndrome associated with physiological disturbance and physical factors	Mental health	snomed
231538003	Behavioural and emotional disorder with onset in childhood	Mental health	snomed
231792001	Ventriculolumbar shunt with valve	Physical illness	snomed
231794000	Injury of globe of eye	Accident and injury	snomed
231795004	Laceration of eye	Accident and injury	snomed
231796003	Cellulitis of eyelid	Physical illness	snomed
231813003	Injury to eyelid	Accident and injury	snomed
231815005	Abrasion of eyelid	Accident and injury	snomed
231816006	Laceration of eyelid	Accident and injury	snomed
231851003	Penetrating wound of orbit	Accident and injury	snomed
231854006	Atopic conjunctivitis	Physical illness	snomed
231863008	Injury of conjunctiva	Accident and injury	snomed
231866000	Conjunctival foreign body	Accident and injury	snomed
231878001	Injury of sclera	Accident and injury	snomed
231883009	Foreign body in posterior wall eye	Accident and injury	snomed
231945007	Chemical injury to cornea	Accident and injury	snomed
231954005	Traumatic hyphaema	Accident and injury	snomed
232009009	Rhegmatogenous retinal detachment − macula off	Physical illness	snomed
232209000	Nasal obstruction	Physical illness	snomed
232212002	Abscess of external ear	Physical illness	snomed
232232001	Acute non−infective otitis externa	Physical illness	snomed
232256002	Otitis media with effusion − sanguinous	Physical illness	snomed
232347008	Animal dander allergy	Physical illness	snomed
232354002	Anterior epistaxis	Physical illness	snomed
232361003	Adhesions of nasal cavity	Physical illness	snomed
232376006	Dislocated nasal septum	Accident and injury	snomed
232377002	Nasal septal haematoma	Physical illness	snomed
232401004	Glandular fever pharyngitis	Physical illness	snomed
23268009	Malingering	Other	snomed
23301003	Infection following infusion, injection, transfusion AND/OR vaccination	Physical illness	snomed
23346002	Sunburn	Accident and injury	snomed
233604007	Pneumonia	Physical illness	snomed

ed_code	description	presentation_category	classification
233606009	Atypical pneumonia	Physical illness	snomed
23361001	Exposure to sting or bite by insect	Accident and injury	snomed
233959009	Upper limb ischaemia	Physical illness	snomed
233983001	Ruptured cerebral aneurysm	Physical illness	snomed
234009005	Arterial laceration	Accident and injury	snomed
23406007	Fracture of upper limb	Accident and injury	snomed
23415000	Sprain of temporomandibular joint	Accident and injury	snomed
234162000	Simple faint (disorder)	Physical illness	snomed
234467004	Thrombophilia	Physical illness	snomed
234532001	Immunodeficiency disorder	Physical illness	snomed
23482006	[Q]Avulsion	Accident and injury	snomed
234947003	Tooth disorder	Physical illness	snomed
234948008	Tooth absent	Physical illness	snomed
235104008	Impacted tooth	Physical illness	snomed
235107001	Mandibular displacement	Accident and injury	snomed
23511006	Meningococcal infectious disease	Physical illness	snomed
23513009	Herpes infection	Physical illness	snomed
23527004	Cannabis intoxication	Alcohol and substance misuse	snomed
235494005	Chronic pancreatitis	Physical illness	snomed
235595009	Gastroesophageal reflux disease	Physical illness	snomed
235599003	Eosinophilic oesophagitis	Physical illness	snomed
235841007	Chronic nonspecific abdominal pain	Physical illness	snomed
235856003	Liver disease	Physical illness	snomed
235861001	Abscess gonococcal	Physical illness	snomed
235993005	Intra−abdominal collection	Physical illness	snomed
236069009	Chronic constipation	Physical illness	snomed
236071009	Chronic diarrhoea	Physical illness	snomed
236423003	Renal impairment	Physical illness	snomed
236425005	CKD − chronic kidney disease	Physical illness	snomed
236433006	Acute−on−chronic renal failure	Physical illness	snomed
2367005	Acute haemorrhagic gastritis	Physical illness	snomed
236725009	Obstruction of urinary stent	Physical illness	snomed
236735003	Obstructed indwelling urinary catheter	Physical illness	snomed
236838009	Drainage of vulval abscess	Physical illness	snomed
237037006	Acute pelvic inflammatory disease	Physical illness	snomed

ed_code	description	presentation_category	classification
237044002	Chronic pelvic inflammatory disease	Physical illness	snomed
237067000	Chronic pelvic pain of female	Physical illness	snomed
237084006	Chlamydial cervicitis	Physical illness	snomed
237091009	Infective vaginitis	Physical illness	snomed
237241002	Viable pregnancy	Physical illness	snomed
237439001	Abscess of nipple	Physical illness	snomed
237557003	Mass of thyroid gland	Physical illness	snomed
237621004	Diabetic severe hyperglycaemia	Physical illness	snomed
237623001	Acute hyperglycaemia	Physical illness	snomed
237632004	Hypoglycaemic event due to diabetes	Physical illness	snomed
238150007	Sepsis syndrome	Physical illness	snomed
238382001	Wound abscess	Accident and injury	snomed
238396007	Cellulitis of dorsum of hand	Physical illness	snomed
238402004	Cellulitis of lower limb	Physical illness	snomed
238408000	Infection of nail	Physical illness	snomed
238491003	Plaster of Paris injury to skin	Physical illness	snomed
238575004	Allergic contact dermatitis	Physical illness	snomed
238722001	Subungual haemorrhage	Physical illness	snomed
238741003	Hair follicle disorder	Physical illness	snomed
23875004	No pathologic diagnosis	Other	snomed
238810007	Flushing	Physical illness	snomed
23900009	Closed fracture of upper end of tibia	Accident and injury	snomed
23913003	External haemorrhoids	Physical illness	snomed
239159001	Wound seroma	Accident and injury	snomed
239164002	Wound discharge	Accident and injury	snomed
23919004	Labyrinthitis	Physical illness	snomed
23924001	Tight chest	Physical illness	snomed
239589000	Amputation of finger through distal phalanx	Accident and injury	snomed
23971007	Acute vomiting	Physical illness	snomed
239720000	Tear of meniscus of knee	Accident and injury	snomed
239725005	Rupture of anterior cruciate ligament	Accident and injury	snomed
239728007	Deficiency of medial collateral ligament of knee	Physical illness	snomed
239733006	Anterior knee pain	Physical illness	snomed
239783001	Post−infective arthritis	Physical illness	snomed
239830003	Entire right foot	Accident and injury	snomed

ed_code	description	presentation_category	classification
23986001	Glaucoma	Physical illness	snomed
239954007	Soft tissue lesion of shoulder region	Physical illness	snomed
239961006	Bursitis of shoulder	Physical illness	snomed
239973006	Tendinitis AND/OR tenosynovitis of wrist AND/OR hand	Accident and injury	snomed
240008008	Synovial cyst of knee	Physical illness	snomed
240025005	Soft tissue lesion of foot region	Physical illness	snomed
240131006	Rhabdomyolysis	Physical illness	snomed
2403008	Psychoactive substance dependence	Alcohol and substance misuse	snomed
240301009	Breastfeeding problem in the newborn	Physical illness	snomed
24059009	Acute cerebellar ataxia caused by varicella	Physical illness	snomed
24063002	Fracture of base of skull	Accident and injury	snomed
240709008	Perineal candidiasis	Physical illness	snomed
24079001	Atopic dermatitis	Physical illness	snomed
241078000	Fibula X−ray	Accident and injury	snomed
241762008	Beta−blocker poisoning	Suicidal behaviours and self−harm	snomed
241817000	Funnel web spider venom poisoning	Accident and injury	snomed
241820008	Bee sting	Accident and injury	snomed
241847003	Bleach causing toxic effect	Suicidal behaviours and self−harm	snomed
241929008	Acute allergic reaction	Physical illness	snomed
24199005	Feeling agitated	Mental health	snomed
242253008	Overdose of opiate	Alcohol and substance misuse	snomed
242591007	Poisoned bite of funnel web spider	Accident and injury	snomed
242822003	Intentional drug or pharmaceutical preparation overdose (disorder)	Suicidal behaviours and self−harm	snomed
242824002	Intentional paracetamol overdose	Suicidal behaviours and self−harm	snomed
242826000	Intentional combined analgesic overdose (disorder)	Suicidal behaviours and self−harm	snomed
242828004	Intentional opiate analgesic overdose	Suicidal behaviours and self−harm	snomed
242832005	Intentional benzodiazepine overdose	Suicidal behaviours and self−harm	snomed
242833000	Intentional diazepam overdose	Suicidal behaviours and self−harm	snomed
242835007	Intentional temazepam overdose	Suicidal behaviours and self−harm	snomed
24288004	Cellulitis of ankle	Physical illness	snomed
242937003	Late effects of assault	Accident and injury	snomed
243321006	Purulent conjunctivitis	Physical illness	snomed
243338005	Nerve root compression syndrome	Accident and injury	snomed
24342007	Infection of cesarean section AND/OR perineal wound	Physical illness	snomed
2435008	Ascariasis	Physical illness	snomed

ed_code	description	presentation_category	classification
243818000	Intrauterine contraceptive device status	Physical illness	snomed
243876005	Screening status	Other	snomed
24424003	Closed fracture of phalanx of finger	Accident and injury	snomed
24526004	Inflammatory bowel disease	Physical illness	snomed
24552005	Retained corpus luteum	Physical illness	snomed
246524009	Ear, nose and throat symptoms	Physical illness	snomed
246545002	Generalised seizure	Physical illness	snomed
246636008	Hazy vision	Physical illness	snomed
24693007	Fibromyositis	Physical illness	snomed
24700007	Multiple sclerosis	Physical illness	snomed
247165009	Retinal detachment − subretinal fluid	Physical illness	snomed
247234006	Ear observation	Physical illness	snomed
247325003	Altered sensation of skin	Physical illness	snomed
247342009	Foreign body sensation	Accident and injury	snomed
247355005	Flank pain	Physical illness	snomed
247358007	Abdominal pain type	Physical illness	snomed
247366003	Acute back pain with sciatica	Physical illness	snomed
247373008	Ankle pain	Physical illness	snomed
247398009	Neuropathic pain	Physical illness	snomed
247441003	Erythema	Physical illness	snomed
247472004	Hives	Physical illness	snomed
247518009	Nail bed observation	Accident and injury	snomed
247650009	Planning suicide	Suicidal behaviours and self−harm	snomed
2477008	Superficial thrombophlebitis	Physical illness	snomed
247730001	Verbal auditory hallucinations	Mental health	snomed
247745008	Dissociative auditory hallucinations	Mental health	snomed
247804008	Schizophrenic prodrome	Mental health	snomed
247825008	Anxiety about behaviour or performance	Mental health	snomed
247917007	Catatonia	Mental health	snomed
248004009	Physical aggression	Mental health	snomed
248039009	Defiant behaviour	Mental health	snomed
248044002	Disruptive behaviour	Mental health	snomed
248061004	Self−harm	Suicidal behaviours and self−harm	snomed
248062006	Deliberate self−harm	Suicidal behaviours and self−harm	snomed
248223005	Feeling faint	Physical illness	snomed

ed_code	description	presentation_category	classification
248228001	Funny turn	Physical illness	snomed
248283007	Illness: Cannot describe symptoms	Other	snomed
248306003	Fatty dimpling of skin	Physical illness	snomed
248325000	Malnourished	Physical illness	snomed
248406004	Ring tight on fingers	Physical illness	snomed
248490000	Bloating symptom	Physical illness	snomed
24850009	Injury of ureter	Accident and injury	snomed
248539004	Family problems	Other	snomed
248554005	Partially obstructed airway	Physical illness	snomed
248870002	Vagina observation	Physical illness	snomed
248898004	Tampon in vagina	Physical illness	snomed
249236000	Testicular observation	Physical illness	snomed
249253007	Foreskin observation	Physical illness	snomed
249306007	Nose symptoms	Physical illness	snomed
249395001	Large tonsils	Physical illness	snomed
249423002	Bleeding from tonsillar bed	Physical illness	snomed
249489001	Choking	Physical illness	snomed
249497008	Vomiting symptom	Physical illness	snomed
249519007	Diarrhoea and vomiting	Physical illness	snomed
249543005	Abdomen soft	Physical illness	snomed
249546002	Finding of movement of abdomen	Physical illness	snomed
24982008	Diplopia	Physical illness	snomed
249847000	Dystonic posture	Physical illness	snomed
249944006	Monoparesis − arm	Physical illness	snomed
249945007	Monoparesis − leg	Physical illness	snomed
250107008	Anterior cruciate instability	Physical illness	snomed
25012008	Retained foreign body of eyelid	Accident and injury	snomed
250243009	Dacrocytosis	Physical illness	snomed
250421003	Pregnancy test observation	Physical illness	snomed
25055007	Ingrowing nail with infection	Physical illness	snomed
25064002	Headache	Physical illness	snomed
25188002	Pediculosis corporis	Physical illness	snomed
2528003	Viraemia	Physical illness	snomed
25374005	Gastroenteritis	Physical illness	snomed
25458004	Acute gastritis	Physical illness	snomed

ed_code	description	presentation_category	classification
254671003	Infection of sebaceous cyst	Physical illness	snomed
25470000	Acute dacryocystitis	Physical illness	snomed
25479004	Foreign body in pharynx	Accident and injury	snomed
254845004	Fibroadenoma of breast	Physical illness	snomed
25501002	Social phobia	Mental health	snomed
255320000	Infection − suppurative	Physical illness	snomed
255339005	Depression − motion	Mental health	snomed
25569003	Ventricular tachycardia	Physical illness	snomed
25658005	Disorder of vagina	Physical illness	snomed
256764004	Abdominal flap	Physical illness	snomed
25702006	Alcohol intoxication	Alcohol and substance misuse	snomed
25786006	Abnormal behaviour	Mental health	snomed
25809009	Victim of trauma	Accident and injury	snomed
25825004	Hemorrhage in early pregnancy	Physical illness	snomed
258743006	Optical density units (qualifier value)	Other	snomed
25899002	Closed bimalleolar fracture	Accident and injury	snomed
25906001	Disorder of ear	Physical illness	snomed
2591000	Crushing injury of shoulder region	Accident and injury	snomed
25924004	Cholelithiasis with cholecystitis	Physical illness	snomed
26079004	Tremor	Physical illness	snomed
260885003	Prescription	Other	snomed
260994008	Bipolar	Mental health	snomed
261665006	Unknown	Other	snomed
262519004	Multiple injuries	Accident and injury	snomed
262520005	Thumb injury	Accident and injury	snomed
262522002	Injury of cervical spine	Accident and injury	snomed
262525000	Chest injury	Accident and injury	snomed
262526004	Wound of skin	Accident and injury	snomed
262528003	Superficial bruising of head	Accident and injury	snomed
262536007	Superficial abrasion	Accident and injury	snomed
262539000	Superficial friction burn	Accident and injury	snomed
262541004	Superficial laceration	Accident and injury	snomed
262550002	Infected insect bite	Accident and injury	snomed
262555007	Human bite − wound	Accident and injury	snomed
262560006	Penetrating wound	Accident and injury	snomed

ed_code	description	presentation_category	classification
262562003	Deep laceration	Accident and injury	snomed
262571007	Deep dog bite	Accident and injury	snomed
262576002	Crushing injury of skull and intracranial contents	Accident and injury	snomed
262582004	Burn of face	Accident and injury	snomed
262596005	Traumatic amputation of fingertip	Accident and injury	snomed
262630008	Abrasion of oral cavity	Accident and injury	snomed
262682005	Injury of vagina	Accident and injury	snomed
262754009	Injury of orbit	Accident and injury	snomed
26284000	Ulcer of mouth	Physical illness	snomed
262965006	Strain of back muscle	Accident and injury	snomed
262966007	Rupture of muscle	Accident and injury	snomed
262990008	Forearm sprain	Accident and injury	snomed
262998001	Sprained toe	Accident and injury	snomed
263019000	Dislocation of acromioclavicular joint	Accident and injury	snomed
263021005	Anterior dislocation of shoulder joint	Accident and injury	snomed
263022003	Posterior dislocation of shoulder joint	Physical illness	snomed
263029007	Dislocation of patellofemoral joint	Accident and injury	snomed
263030002	Dislocation of toe joint	Accident and injury	snomed
263049003	Subluxation of acromioclavicular joint	Accident and injury	snomed
263051004	Subluxation of shoulder joint	Accident and injury	snomed
263078002	Fracture dislocation of elbow joint	Accident and injury	snomed
263092008	Fracture dislocation of foot joint	Accident and injury	snomed
263093003	Fracture dislocation of toe joint	Accident and injury	snomed
263125003	Volar plate injury of finger joint	Accident and injury	snomed
263126002	Ligament injury	Accident and injury	snomed
263128001	Sprain of ligament of elbow	Accident and injury	snomed
263129009	Sprain of finger	Accident and injury	snomed
263133002	Sprain of lateral ligament of ankle joint	Accident and injury	snomed
263139003	Rupture of ligament of knee joint	Accident and injury	snomed
263151001	Fracture of upper jaw, closed	Accident and injury	snomed
263167007	Blow out fracture of orbit	Accident and injury	snomed
26317001	Acute ulcer	Physical illness	snomed
263171005	Fractured nasal bones	Accident and injury	snomed
263172003	Fracture of mandible	Accident and injury	snomed
263191003	Fracture of neck of humerus	Accident and injury	snomed

ed_code	description	presentation_category	classification
263193000	Supracondylar fracture of humerus	Accident and injury	snomed
263196008	Fracture of radial head	Accident and injury	snomed
263197004	Fracture of radial neck	Accident and injury	snomed
263198009	Fracture of shaft of radius	Accident and injury	snomed
263199001	Fracture of distal end of radius	Accident and injury	snomed
263205008	Fracture of distal end of ulna	Accident and injury	snomed
263208005	Fracture of distal end of radius and ulna	Accident and injury	snomed
263209002	Fracture of base of fifth metacarpal	Accident and injury	snomed
263210007	Fracture of neck of fifth metacarpal	Accident and injury	snomed
26322001	Bleeding from ear	Physical illness	snomed
263233008	Closed fracture of femur, distal end	Accident and injury	snomed
263242001	Fracture of distal end of fibula	Accident and injury	snomed
263244000	Bimalleolar fracture of ankle	Accident and injury	snomed
263246003	Fracture of talus	Accident and injury	snomed
263247007	Fracture of calcaneus	Accident and injury	snomed
263248002	Fracture of navicular	Accident and injury	snomed
263249005	Fracture of cuboid	Accident and injury	snomed
263251009	Metatarsal bone fracture	Accident and injury	snomed
26330000	Cellulitis of perineum	Physical illness	snomed
263476007	Entire head of fifth metacarpal bone	Accident and injury	snomed
26373009	Thrombosed external haemorrhoids	Physical illness	snomed
263939004	Anal	Physical illness	snomed
26416006	Drug abuse	Alcohol and substance misuse	snomed
26421009	Bleeding external haemorrhoids	Physical illness	snomed
26435006	Acute dermatitis	Physical illness	snomed
264973001	Diagnostic laparoscopy of female pelvis	Physical illness	snomed
266108008	Hand, foot and mouth disease	Physical illness	snomed
266257000	Transient ischaemic attack	Physical illness	snomed
266364000	Asthma attack	Physical illness	snomed
266433003	Gastroesophageal reflux disease with oesophagitis	Physical illness	snomed
266474003	Cholelithiasis	Physical illness	snomed
26650005	Acute tracheitis	Physical illness	snomed
266549004	Nephrotic syndrome with minimal change glomerulonephritis	Physical illness	snomed
266571009	Acquired phimosis	Physical illness	snomed
266579006	Inflammatory disorder of breast	Physical illness	snomed

ed_code	description	presentation_category	classification
266599000	Dysmenorrhoea	Physical illness	snomed
26665006	Antisocial personality disorder	Mental health	snomed
266707007	Drug addiction therapy	Alcohol and substance misuse	snomed
26677001	Sleep pattern disturbance	Mental health	snomed
266935003	Housing lack	Other	snomed
266949007	Home problems	Other	snomed
267036007	Dyspnoea	Physical illness	snomed
267038008	Oedema	Physical illness	snomed
267039000	Swollen ankle	Physical illness	snomed
267041004	Swelling around eyes	Physical illness	snomed
267064002	Urinary retention	Physical illness	snomed
267073005	Suicidal	Suicidal behaviours and self−harm	snomed
267078001	Involuntary movement	Physical illness	snomed
267096005	Frontal headache	Physical illness	snomed
267102003	Sore throat symptom	Physical illness	snomed
267103008	Feeling of lump in throat	Physical illness	snomed
267548000	Nonspecific mesenteric adenitis	Physical illness	snomed
267949000	Shoulder joint pain	Physical illness	snomed
268617001	Acute schizophrenic episode	Mental health	snomed
268619003	Manic disorder, single episode	Mental health	snomed
26883000	Abscess of knee	Physical illness	snomed
26889001	Myositis	Physical illness	snomed
268986002	O/E − foreign body in skin	Accident and injury	snomed
269083002	Closed Colles' fracture	Accident and injury	snomed
269100000	Fracture of one or more tarsal and metatarsal bones	Accident and injury	snomed
269111008	Dislocation or subluxation of finger or thumb	Accident and injury	snomed
269166009	Broken tooth injury	Accident and injury	snomed
269177003	Open wound of finger(s) or thumb	Accident and injury	snomed
269243001	Epidermal burn of wrist and hand	Accident and injury	snomed
269329008	Arm sprain − upper	Accident and injury	snomed
269406001	Post−traumatic wound infection	Accident and injury	snomed
269765000	Accidental alcohol poisoning	Alcohol and substance misuse	snomed
269833008	Infectious mononucleosis test positive	Physical illness	snomed
270426007	Did not attend − no reason	Other	snomed
270428008	Malingerer	Other	snomed

ed_code	description	presentation_category	classification
270476009	Wry neck/torticollis	Accident and injury	snomed
270490007	Acute otitis media with effusion	Physical illness	snomed
270524007	Mechanical complication of intrauterine contraceptive device	Physical illness	snomed
270911002	Hematoma with intact skin	Accident and injury	snomed
271161002	Traumatic blister of forearm	Accident and injury	snomed
271177001	Traumatic blister of lower leg	Accident and injury	snomed
271189004	Traumatic blister of toe	Accident and injury	snomed
271364002	Awaiting housing or re−housing	Other	snomed
271558008	Infectious mononucleosis	Physical illness	snomed
271594007	Syncope	Physical illness	snomed
271596009	Mental distress	Mental health	snomed
271614004	Submandibular salivary gland swelling	Physical illness	snomed
271687003	Swelling of scrotum	Physical illness	snomed
271737000	Anaemia	Physical illness	snomed
271739002	Wax in ear canal	Physical illness	snomed
271782001	Drowsy	Physical illness	snomed
271787007	Collapse	Physical illness	snomed
271789005	Dizziness and giddiness	Physical illness	snomed
271794005	Disorder of sleep−wake cycle	Physical illness	snomed
271807003	Rash	Physical illness	snomed
271809000	Peripheral oedema	Physical illness	snomed
271813007	Petechiae	Physical illness	snomed
27182002	Sprain of acromioclavicular ligament	Accident and injury	snomed
271825005	Respiratory distress	Physical illness	snomed
271857006	Loin pain	Physical illness	snomed
271893008	O/E − behavioral	Mental health	snomed
271939006	Vaginal discharge	Physical illness	snomed
271952001	Stress and adjustment reaction	Mental health	snomed
271953006	Obsessional thoughts of causing harm to self	Mental health	snomed
271973001	Disorder of intrauterine contraceptive device	Physical illness	snomed
271983002	Disorder of cardiac pacemaker system	Physical illness	snomed
272021002	C/O − ureteric colic	Physical illness	snomed
272025006	Complaining of insomnia	Mental health	snomed
272027003	C/O − a headache	Physical illness	snomed
272030005	Syncope symptom	Physical illness	snomed

ed_code	description	presentation_category	classification
272039006	C/O − cough	Physical illness	snomed
272044004	C/O − vomiting	Physical illness	snomed
27296002	Psychiatric	Mental health	snomed
2733002	Heel pain	Physical illness	snomed
27342004	Anaemia of pregnancy	Physical illness	snomed
27355003	Toothache	Physical illness	snomed
274080003	Bacterial gastroenteritis	Physical illness	snomed
274112000	Male genital abscess	Physical illness	snomed
274162005	Thoracic back sprain	Physical illness	snomed
274164006	Minor head injury	Accident and injury	snomed
274165007	Laceration of skin	Accident and injury	snomed
274166008	Scalp laceration	Accident and injury	snomed
274171001	Laceration of back	Accident and injury	snomed
274172008	Laceration of finger	Accident and injury	snomed
274173003	Laceration of thumb	Accident and injury	snomed
274179004	Traumatic haematoma	Accident and injury	snomed
274182009	Superficial injury of neck	Accident and injury	snomed
274191008	Superficial injury of shoulder	Accident and injury	snomed
274192001	Superficial injury of wrist	Accident and injury	snomed
274196003	Superficial injury of hip	Accident and injury	snomed
274204004	Burn of cornea	Accident and injury	snomed
274205003	Burn of eye region	Accident and injury	snomed
274221008	Wound − in medical care	Accident and injury	snomed
274224000	Foreign body accident	Accident and injury	snomed
274277005	C/O right iliac fossa pain	Physical illness	snomed
274279008	Renal pain	Physical illness	snomed
274287009	O/E − abdominal pain	Physical illness	snomed
274288004	O/E − epigastric pain	Physical illness	snomed
27431007	Fibrocystic disease of breast	Physical illness	snomed
274662006	Transient limb paralysis	Physical illness	snomed
274663001	Acute pain	Physical illness	snomed
274664007	Chest pain on breathing	Physical illness	snomed
274665008	Chronic intractable pain	Physical illness	snomed
274667000	Jaw pain	Physical illness	snomed
274668005	Non−cardiac chest pain	Physical illness	snomed

ed_code	description	presentation_category	classification
274671002	Pelvic and perineal pain	Physical illness	snomed
274676007	Tingling of skin	Physical illness	snomed
274734008	Urination frequency and polyuria	Physical illness	snomed
274740001	Enlarged submandibular lymph gland	Physical illness	snomed
274743004	Swelling of inguinal region	Physical illness	snomed
274747003	Localised swelling, mass and lump, neck	Physical illness	snomed
274755005	Head and neck swelling	Physical illness	snomed
27477003	Fracture of facial bones	Accident and injury	snomed
274776000	Finding of alcohol in blood	Alcohol and substance misuse	snomed
274951009	Outbursts of anger	Mental health	snomed
274952002	Borderline schizophrenia	Mental health	snomed
275320004	Injury of nail	Accident and injury	snomed
275334004	Shoulder strain	Physical illness	snomed
275335003	Ruptured Achilles tendon − traumatic	Accident and injury	snomed
275393007	Oral infection	Physical illness	snomed
275413005	Blocked catheter	Physical illness	snomed
275453008	Foreign body − finger	Accident and injury	snomed
275454002	Foreign body in thumb	Accident and injury	snomed
275457009	Open wound of finger or thumb with tendon involvement	Accident and injury	snomed
275461003	Wound of toenail	Accident and injury	snomed
275476006	Foreign body under eyelid	Accident and injury	snomed
275498002	Respiratory tract infection	Physical illness	snomed
275570002	Complaining of per vaginam bleeding	Physical illness	snomed
27561001	Cellulitis of finger	Physical illness	snomed
275813002	Emergency contraception	Physical illness	snomed
275814008	Morning after pill 900000000000020002<br>1225447015 20040131 0 900000000000207008 275814008 en 900000000000013009 Morning after	Other	snomed
275869006	O/E − skin lesion	Physical illness	snomed
275897000	O/E − soft tissue swelling	Physical illness	snomed
27601005	Helminth infection	Physical illness	snomed
276369006	Reduced fetal movement	Physical illness	snomed
276433004	Insect bite	Accident and injury	snomed
276464002	Injury of anus	Accident and injury	snomed
276796006	Atrial tachycardia	Physical illness	snomed
276853009	Self inflicted injury	Suicidal behaviours and self−harm	snomed
277131000	Intentional non−steroidal anti−inflammatory agent overdose	Suicidal behaviours and self−harm	snomed

ed_code	description	presentation_category	classification
277491009	Peripheral positional vertigo	Physical illness	snomed
2776000	Delirium	Physical illness	snomed
277843001	Behavioural problem	Mental health	snomed
277890004	Toe swelling	Physical illness	snomed
278086000	Baseline tachycardia	Physical illness	snomed
278286009	Right hemiparesis	Physical illness	snomed
278287000	Left hemiparesis	Physical illness	snomed
27836007	Pertussis	Physical illness	snomed
278516003	Lobar pneumonia	Physical illness	snomed
278528006	Facial swelling	Physical illness	snomed
278537006	Fracture of distal end of tibia	Accident and injury	snomed
27885002	Complete atrioventricular block	Physical illness	snomed
278852008	Paranoid−hallucinatory epileptic psychosis	Mental health	snomed
278853003	Acute schizophrenia−like psychotic disorder	Mental health	snomed
278860009	Chronic lower back pain	Physical illness	snomed
278862001	Acute low back pain	Physical illness	snomed
279038004	Thoracic back pain	Physical illness	snomed
279039007	Low back pain	Physical illness	snomed
279040009	Mechanical low back pain	Physical illness	snomed
279043006	Pain in buttock	Physical illness	snomed
279066007	Foot joint pain	Physical illness	snomed
279069000	Musculoskeletal pain	Physical illness	snomed
279084009	Chest discomfort	Physical illness	snomed
27942005	Shock	Physical illness	snomed
27956007	Cocaine intoxication	Alcohol and substance misuse	snomed
279572002	Testicle structure	Physical illness	snomed
279992002	Recurrent falls	Physical illness	snomed
280133005	Disorder of soft tissue of trunk	Physical illness	snomed
280137006	Diabetic foot	Physical illness	snomed
280427006	Psychotic symptom present	Mental health	snomed
280816001	Facial palsy	Physical illness	snomed
280965007	Behavioral disability	Mental health	snomed
281164004	Foreign body in foot	Accident and injury	snomed
281239006	Exacerbation of asthma	Physical illness	snomed
281245003	Musculoskeletal chest pain	Physical illness	snomed

ed_code	description	presentation_category	classification
281254000	Large bowel obstruction	Physical illness	snomed
281255004	Small bowel obstruction	Physical illness	snomed
281368005	Intra−articular foreign body	Accident and injury	snomed
281398003	Groin mass	Physical illness	snomed
281399006	Did not attend	Other	snomed
281503004	Dislocation of carpometacarpal joint	Accident and injury	snomed
281521001	Traumatic effusion of joint of knee	Accident and injury	snomed
281527002	Fracture of radial styloid	Accident and injury	snomed
281528007	Fracture of olecranon	Accident and injury	snomed
281530009	Fracture of ulnar styloid	Accident and injury	snomed
281531008	Fracture of medial malleolus	Accident and injury	snomed
281535004	Fracture of lateral malleolus	Accident and injury	snomed
281544003	Strain of tendon of foot and ankle	Accident and injury	snomed
281598004	Sprain of spinal ligament	Accident and injury	snomed
281794004	Viral upper respiratory tract infection	Physical illness	snomed
281900007	No abnormality detected	Other	snomed
281923005	Crush fracture of thoracic vertebra	Accident and injury	snomed
281934008	Burst fracture of lumbar vertebra	Accident and injury	snomed
282025003	Labial cyst	Physical illness	snomed
282026002	Soft tissue injury	Accident and injury	snomed
282027006	Normal menstruation	Physical illness	snomed
282095007	Allergic reaction to bee sting	Physical illness	snomed
282100009	Adverse reaction to substance	Alcohol and substance misuse	snomed
282293007	Diagnosis not made	Other	snomed
282360001	Persistent testicular pain	Physical illness	snomed
282749008	Head and neck injury	Accident and injury	snomed
282752000	Injury of eye region	Accident and injury	snomed
282756002	Jaw injury	Accident and injury	snomed
282757006	Cheek injury	Accident and injury	snomed
282760004	Clavicle injury	Accident and injury	snomed
282762007	Fingernail injury	Accident and injury	snomed
282766005	Lower back injury	Accident and injury	snomed
282770002	Injury of ribs	Accident and injury	snomed
282771003	Pelvic injury	Accident and injury	snomed
282772005	Genital injury	Accident and injury	snomed

ed_code	description	presentation_category	classification
282776008	Injury of toe	Accident and injury	snomed
282780003	Heel injury	Accident and injury	snomed
282781004	Injury of tympanic membrane	Accident and injury	snomed
283025007	Superficial injury of head	Accident and injury	snomed
283026008	Superficial injury of ear region	Accident and injury	snomed
283042001	Superficial injury of great toe	Accident and injury	snomed
283049005	Abrasion of head	Accident and injury	snomed
283050005	Abrasion of eye region	Accident and injury	snomed
283058003	Abrasion of wrist	Accident and injury	snomed
283059006	Abrasion of hand	Accident and injury	snomed
283062009	Abrasion of lower limb	Accident and injury	snomed
283115000	Traumatic blister of head	Accident and injury	snomed
283126006	Traumatic blister of finger	Accident and injury	snomed
283138002	Contusion of dorsum of foot	Accident and injury	snomed
283189002	Fishing hook foreign body	Accident and injury	snomed
283268000	Glass in lower leg	Accident and injury	snomed
283293003	Wood splinter in finger	Accident and injury	snomed
283330004	Insect bite of upper limb	Accident and injury	snomed
283336005	Insect bite of trunk	Accident and injury	snomed
283339003	Insect bite of lower limb	Accident and injury	snomed
283355005	Laceration of oral cavity	Accident and injury	snomed
283357002	Laceration of lower limb	Accident and injury	snomed
283359004	Laceration of forehead	Accident and injury	snomed
283360009	Laceration of eye region	Accident and injury	snomed
283361008	Laceration of ear region	Accident and injury	snomed
283362001	Laceration of cheek	Accident and injury	snomed
283363006	Laceration of lip	Accident and injury	snomed
283364000	Laceration of neck	Accident and injury	snomed
283366003	Laceration of upper limb	Accident and injury	snomed
283367007	Laceration of shoulder	Accident and injury	snomed
283370006	Laceration of elbow	Accident and injury	snomed
283371005	Laceration of forearm	Accident and injury	snomed
283372003	Laceration of wrist	Accident and injury	snomed
283373008	Laceration of dorsum of hand	Accident and injury	snomed
283374002	Laceration of palm of hand	Accident and injury	snomed

ed_code	description	presentation_category	classification
283378004	Laceration of abdomen	Accident and injury	snomed
283383007	Laceration of buttock	Accident and injury	snomed
283384001	Laceration of hip	Accident and injury	snomed
283385000	Laceration of thigh	Accident and injury	snomed
283386004	Laceration of knee	Accident and injury	snomed
283387008	Laceration of lower leg	Accident and injury	snomed
283390002	Laceration of ankle	Accident and injury	snomed
283391003	Laceration of great toe	Accident and injury	snomed
283475002	Stab wound of abdomen	Accident and injury	snomed
283514006	Puncture wound of elbow	Accident and injury	snomed
283517004	Puncture wound of hand	Accident and injury	snomed
283536009	Puncture wound of lower leg	Accident and injury	snomed
283543003	Puncture wound of sole of foot	Accident and injury	snomed
283596007	Needle prick injury	Accident and injury	snomed
283619007	Needle stick injury of finger	Accident and injury	snomed
283682007	Bite − wound	Accident and injury	snomed
283684008	Human bite of head and neck	Accident and injury	snomed
283691006	Human bite of ear region	Accident and injury	snomed
283693009	Human bite of cheek	Accident and injury	snomed
283734005	Dog bite − wound	Accident and injury	snomed
283735006	Dog bite of head and neck	Accident and injury	snomed
283738008	Dog bite of face	Accident and injury	snomed
283745008	Dog bite of neck	Accident and injury	snomed
283747000	Dog bite of upper limb	Accident and injury	snomed
283752005	Dog bite of forearm	Accident and injury	snomed
283753000	Dog bite of wrist	Accident and injury	snomed
283754006	Dog bite of hand	Accident and injury	snomed
283756008	Dog bite of palm of hand	Accident and injury	snomed
283759001	Dog bite of trunk	Accident and injury	snomed
283767009	Dog bite of lower limb	Accident and injury	snomed
283770008	Dog bite of thigh	Accident and injury	snomed
283772000	Dog bite of lower leg	Accident and injury	snomed
283782004	Cat bite − wound	Accident and injury	snomed
283840005	Snake bite − wound	Accident and injury	snomed
283858004	Crush injury of thumb	Accident and injury	snomed

ed_code	description	presentation_category	classification
283859007	Crush injury of pelvic region	Accident and injury	snomed
283949007	Laceration of penis	Accident and injury	snomed
284002000	Injury of dental structures	Accident and injury	snomed
284075002	Spotting per vagina in pregnancy	Physical illness	snomed
284212002	Burn of digits of hand	Accident and injury	snomed
284227002	Burn of ankle and foot	Accident and injury	snomed
28432003	Abscess of breast	Physical illness	snomed
28442001	Polyuria	Physical illness	snomed
284424006	Examination of scrotal contents	Physical illness	snomed
284476005	Superficial laceration of face	Accident and injury	snomed
284480000	Cellulitis of arm	Physical illness	snomed
284499009	Maladaptive behavior	Mental health	snomed
284504009	Difficult to manage behaviour	Mental health	snomed
284513006	Manic behaviour	Mental health	snomed
284521000	Pitting oedema	Physical illness	snomed
284549007	Laceration of hand	Accident and injury	snomed
284551006	Laceration of foot	Accident and injury	snomed
284552004	Laceration of toe	Accident and injury	snomed
284554003	Avulsion − injury	Accident and injury	snomed
284612008	Difficulty controlling aggression	Mental health	snomed
284614009	Threatening behaviour	Mental health	snomed
284616006	Verbally threatening behaviour	Mental health	snomed
284754000	Pulling out sutures	Other	snomed
285035003	Unable to manage medicine	Other	snomed
285037006	Does not manage medication	Other	snomed
285302001	Cutaneous cyst	Physical illness	snomed
285335006	Bleeding tooth socket	Physical illness	snomed
285344007	Viral gastritis	Physical illness	snomed
285345008	Multiple lacerations	Accident and injury	snomed
285348005	Strain of abdominal muscle	Accident and injury	snomed
285365001	Pain in toe	Physical illness	snomed
285375003	Pain in penis	Physical illness	snomed
285381006	Acute infective exacerbation of COPD	Physical illness	snomed
285385002	Left sided chest pain	Physical illness	snomed
285386001	Right sided chest pain	Physical illness	snomed

ed_code	description	presentation_category	classification
285387005	Left sided abdominal pain	Physical illness	snomed
285388000	Right sided abdominal pain	Physical illness	snomed
28651003	Orthostatic hypotension	Physical illness	snomed
286756000	Impulsive character	Mental health	snomed
28691000	Abscess of leg, except foot	Physical illness	snomed
286933003	Confusional state	Physical illness	snomed
287045000	Pain in left upper limb	Physical illness	snomed
287046004	Pain in right upper limb	Physical illness	snomed
287047008	Pain in left lower limb	Physical illness	snomed
287048003	Pain in right lower limb	Physical illness	snomed
287075005	Fracture malunion − hand	Accident and injury	snomed
287097007	Sprained finger/thumb	Accident and injury	snomed
287181000	Attempted suicide − hanging	Suicidal behaviours and self−harm	snomed
28743005	Productive cough	Physical illness	snomed
288217001	Rupture of tendon of hand	Accident and injury	snomed
288241003	Pain in limb − multiple	Physical illness	snomed
28826002	Sialolithiasis	Physical illness	snomed
288293001	CNS drug poisoning	Suicidal behaviours and self−harm	snomed
289195008	Slurred speech	Physical illness	snomed
289209003	Pregnancy problem	Physical illness	snomed
289260002	Epigastric hernia	Physical illness	snomed
289476008	Swelling of labia	Physical illness	snomed
289485008	Erythema of vulva	Physical illness	snomed
289515002	Bulging of vaginal wall	Physical illness	snomed
289530006	Vaginal bleeding	Physical illness	snomed
289538004	Scanty vaginal bleeding	Physical illness	snomed
289543006	Vaginal bleeding problem	Physical illness	snomed
289567003	Vaginal discharge problem	Physical illness	snomed
289889009	Variation in quantity of menstrual flow	Physical illness	snomed
289894009	Menstrual bleeding present	Physical illness	snomed
289896006	Problem of menstruation	Physical illness	snomed
289900009	Period pain present	Physical illness	snomed
289908002	Pregnancy, function (observable entity)	Physical illness	snomed
289909005	Labor, function (observable entity)	Physical illness	snomed
290031007	Plaster of Paris backslab	Accident and injury	snomed

ed_code	description	presentation_category	classification
2901004	Melaena	Physical illness	snomed
290134002	Accidental paracetamol poisoning	Accident and injury	snomed
29050005	Acute iritis	Physical illness	snomed
290802009	Lithium poisoning	Suicidal behaviours and self−harm	snomed
290858001	Intentional tricyclic antidepressant poisoning	Suicidal behaviours and self−harm	snomed
291121009	Antipsychotic agent poisoning	Suicidal behaviours and self−harm	snomed
2919008	Nausea, vomiting and diarrhoea	Physical illness	snomed
292414000	Amphetamine group adverse reaction	Alcohol and substance misuse	snomed
295117008	Over the counter product overdose	Suicidal behaviours and self−harm	snomed
295119006	Intentional over−the−counter product overdose	Suicidal behaviours and self−harm	snomed
295122008	Overdose of analgesic drug	Suicidal behaviours and self−harm	snomed
295123003	Overdose of nonopioid analgesic	Alcohol and substance misuse	snomed
295124009	Paracetamol overdose	Suicidal behaviours and self−harm	snomed
295171003	Accidental overdose by codeine	Alcohol and substance misuse	snomed
295174006	Heroin overdose	Alcohol and substance misuse	snomed
295175007	Accidental heroin overdose	Alcohol and substance misuse	snomed
295217003	Non−steroidal anti−inflammatory overdose	Suicidal behaviours and self−harm	snomed
295250003	Ibuprofen overdose	Suicidal behaviours and self−harm	snomed
295251004	Accidental ibuprofen overdose	Alcohol and substance misuse	snomed
295252006	Intentional ibuprofen overdose	Suicidal behaviours and self−harm	snomed
295257000	Indomethacin overdose of undetermined intent	Suicidal behaviours and self−harm	snomed
295497000	Nitrous oxide overdose	Alcohol and substance misuse	snomed
29555009	Retinal disorder	Physical illness	snomed
295830007	Overdose of antidepressant drug	Suicidal behaviours and self−harm	snomed
295831006	Lithium overdose	Suicidal behaviours and self−harm	snomed
295833009	Intentional lithium overdose	Suicidal behaviours and self−harm	snomed
295937006	Intentional fluoxetine overdose	Suicidal behaviours and self−harm	snomed
295978008	Overdose of sodium valproate	Suicidal behaviours and self−harm	snomed
296015009	Sedative overdose	Suicidal behaviours and self−harm	snomed
296018006	Intentional zopiclone overdose	Suicidal behaviours and self−harm	snomed
296036006	Barbiturate overdose	Suicidal behaviours and self−harm	snomed
296053004	Benzodiazepine overdose	Alcohol and substance misuse	snomed
296080006	Accidental alprazolam overdose	Alcohol and substance misuse	snomed
296108003	Intentional oxazepam overdose	Suicidal behaviours and self−harm	snomed
296125001	Overdose of temazepam	Alcohol and substance misuse	snomed

ed_code	description	presentation_category	classification
296128004	Accidental overdose of benzodiazepine	Alcohol and substance misuse	snomed
296129007	Overdose of benzodiazepine of undetermined intent	Alcohol and substance misuse	snomed
296291005	Amphetamine overdose	Alcohol and substance misuse	snomed
296292003	Accidental amphetamine overdose	Alcohol and substance misuse	snomed
296293008	Intentional amphetamine overdose	Suicidal behaviours and self−harm	snomed
296301000	Cannabis overdose	Alcohol and substance misuse	snomed
296323001	Intentional overdose by cocaine	Suicidal behaviours and self−harm	snomed
296329002	Caffeine overdose	Alcohol and substance misuse	snomed
296335002	Overdose of beta−adrenergic blocking drug	Suicidal behaviours and self−harm	snomed
296340005	Propranolol overdose	Suicidal behaviours and self−harm	snomed
296382009	Intentional salbutamol overdose	Suicidal behaviours and self−harm	snomed
296393006	Anticholinergic drug overdose	Suicidal behaviours and self−harm	snomed
297062008	Digoxin overdose	Suicidal behaviours and self−harm	snomed
297142003	Foot swelling	Physical illness	snomed
297186008	Motorcycle accident	Accident and injury	snomed
297193007	Ganglion of hand	Physical illness	snomed
297199006	Accidental overdose of opiate	Alcohol and substance misuse	snomed
297200009	Overdose of tricyclic antidepressant	Suicidal behaviours and self−harm	snomed
297201008	Intentional overdose of tricyclic antidepressant	Suicidal behaviours and self−harm	snomed
297217002	Rib pain	Physical illness	snomed
29738008	Proteinuria	Physical illness	snomed
29749002	Closed fracture of scapula	Accident and injury	snomed
29753000	Partial seizure	Physical illness	snomed
297960002	Mass of skin	Physical illness	snomed
298005009	Observation of wound healing	Accident and injury	snomed
298010008	Wound dirty	Accident and injury	snomed
298055001	Flap laceration of skin	Accident and injury	snomed
298065007	Foreign body of skin of lip	Accident and injury	snomed
298068009	Foreign body of skin of ear region	Accident and injury	snomed
298078007	Foreign body of skin of forearm	Accident and injury	snomed
298083004	Foreign body of skin of finger	Accident and injury	snomed
298092001	Foreign body of skin of lower limb	Accident and injury	snomed
298098002	Foreign body of skin of lower leg	Accident and injury	snomed
298102005	Foreign body of skin of toe	Accident and injury	snomed
298109001	Ectopic	Physical illness	snomed

ed_code	description	presentation_category	classification
298292009	Pain on movement of skeletal muscle	Physical illness	snomed
298343000	Observation of falls	Physical illness	snomed
298349001	Soft tissue swelling	Accident and injury	snomed
29857009	Chest pain	Physical illness	snomed
298729007	Sternum tender	Physical illness	snomed
298731003	Pain of sternum	Physical illness	snomed
298856001	Tenderness of shoulder joint	Physical illness	snomed
298872009	Soft tissue swelling of elbow joint	Accident and injury	snomed
298929004	Elbow joint − painful on movement	Accident and injury	snomed
299037003	Swelling of hand	Physical illness	snomed
299060006	Swelling of finger	Physical illness	snomed
299219007	Amputated finger	Accident and injury	snomed
299322007	Swelling of knee joint	Physical illness	snomed
299377003	Knee joint painful on movement	Physical illness	snomed
299414004	Swelling of ankle joint	Physical illness	snomed
299416002	Soft tissue swelling of ankle joint	Accident and injury	snomed
29943008	Herpes simplex dendritic keratitis	Physical illness	snomed
299447008	Ankle joint − painful on movement	Physical illness	snomed
299703001	Mass of neck	Physical illness	snomed
299709002	Dental abscess	Physical illness	snomed
299989006	Infection of toe	Physical illness	snomed
299990002	Infection of foot	Physical illness	snomed
299991003	Infection of finger	Physical illness	snomed
300071000	Infected tooth socket	Physical illness	snomed
300090008	Finding of odour of ear	Physical illness	snomed
300132001	Ear discharge	Physical illness	snomed
300231003	Removal of foreign body from mouth	Accident and injury	snomed
30037006	Anal fissure	Physical illness	snomed
30041005	Acute angle−closure glaucoma	Physical illness	snomed
300471006	Finding of frequency of urination	Physical illness	snomed
300479008	Genital observation	Physical illness	snomed
300569009	Perineal lump	Physical illness	snomed
3006004	Altered consciousness	Mental health	snomed
300848003	Mass of body structure	Physical illness	snomed
300863000	Mass of axilla	Physical illness	snomed

ed_code	description	presentation_category	classification
300874009	Swelling of ear	Physical illness	snomed
300889000	Swelling of arm	Physical illness	snomed
300895004	Anxiety attack	Mental health	snomed
300909004	Allergic reaction to wasp sting	Physical illness	snomed
300932000	Uvulitis	Physical illness	snomed
300953009	Pain in axilla	Physical illness	snomed
300954003	Pain in calf	Physical illness	snomed
300955002	Pain in thumb	Physical illness	snomed
300959008	Allergic cough	Physical illness	snomed
300980002	Normocytic anaemia	Physical illness	snomed
30128009	Tietze's disease	Physical illness	snomed
301350008	Lesion of lip	Physical illness	snomed
301353005	Pain of eye structure	Physical illness	snomed
301365009	Pain of head and neck region	Physical illness	snomed
301410009	Tenderness of right iliac fossa	Physical illness	snomed
301453009	Tendon laceration	Accident and injury	snomed
301467000	Hyperextension of lumbar spine	Accident and injury	snomed
30164005	Signed out against medical advice (procedure)	Other	snomed
301706005	Abscess of foot	Physical illness	snomed
301710008	Cellulitis of elbow	Physical illness	snomed
301711007	Abscess of elbow	Physical illness	snomed
301716002	Left lower quadrant pain	Physical illness	snomed
301717006	Right upper quadrant pain	Physical illness	snomed
301754002	Right lower quadrant pain	Physical illness	snomed
301822002	Abnormal vaginal bleeding	Physical illness	snomed
301924000	Normal globe	Physical illness	snomed
302013002	Carbuncle of upper limb	Physical illness	snomed
302016005	Carbuncle of lower limb	Physical illness	snomed
302222008	Elbow fracture − closed	Accident and injury	snomed
302283001	Helps with dressing	Accident and injury	snomed
30250000	Acute otitis externa	Physical illness	snomed
302533005	Phalanx of toe	Accident and injury	snomed
302866003	Hypoglycaemia	Physical illness	snomed
302932006	Tear of medial meniscus of knee	Accident and injury	snomed
302933001	Tear of lateral meniscus of knee	Accident and injury	snomed

ed_code	description	presentation_category	classification
302941001	Nonunion of fracture	Accident and injury	snomed
302964004	Dislocation of temporomandibular joint	Accident and injury	snomed
304527002	Acute asthma	Physical illness	snomed
304542004	Nonspecific abdominal pain	Physical illness	snomed
304594002	Suicidal intent	Suicidal behaviours and self−harm	snomed
30473006	Pelvic pain	Physical illness	snomed
304838007	Injury of trochlear nerve	Accident and injury	snomed
30549001	Removal of suture	Other	snomed
30556007	Recurrent dislocation of shoulder region	Accident and injury	snomed
305721007	Seen by ophthalmologist	Physical illness	snomed
305865005	Seen by psychiatry service (finding)	Mental health	snomed
305874007	Seen by gynaecology service	Physical illness	snomed
307052004	Illicit drug use	Alcohol and substance misuse	snomed
307131005	Mallory−Weiss tear	Physical illness	snomed
307136000	Old healed fracture of bone	Accident and injury	snomed
307176005	Acute sciatica	Physical illness	snomed
307212006	Brain ventricular shunt malfunction	Physical illness	snomed
30731004	Glossodynia	Physical illness	snomed
307390004	Haematoma of groin	Accident and injury	snomed
307391000	Haematoma of thigh	Accident and injury	snomed
30746006	Lymphadenopathy	Physical illness	snomed
307496006	Diverticulitis	Physical illness	snomed
307534009	Urinary tract infection in pregnancy	Physical illness	snomed
307541003	Lower urinary tract symptoms	Physical illness	snomed
307733001	Inevitable miscarriage complete	Physical illness	snomed
307737000	Inevitable miscarriage incomplete	Physical illness	snomed
30800001	Vaginitis	Physical illness	snomed
308153009	Closed fracture of distal fibula	Accident and injury	snomed
308273005	Follow−up status	Other	snomed
308492005	Contusion − lesion (morphologic abnormality)	Accident and injury	snomed
308904008	Haematemesis − cause unknown	Physical illness	snomed
308921004	Neurological symptom	Physical illness	snomed
308923001	Eye symptom	Physical illness	snomed
309016009	Referred to person	Other	snomed
309018005	Referred to service	Other	snomed

ed_code	description	presentation_category	classification
309083007	Abscess of back	Physical illness	snomed
309462008	Infection of big toe	Physical illness	snomed
309464009	Elbow fracture	Accident and injury	snomed
309521004	Numbness of hand	Physical illness	snomed
309536001	Numbness of finger	Physical illness	snomed
309537005	Numbness of lower limb	Physical illness	snomed
309538000	Numbness of foot	Physical illness	snomed
309557009	Numbness of face	Physical illness	snomed
309585006	Syncope and collapse	Physical illness	snomed
3097002	Superficial injury of lip with infection	Accident and injury	snomed
309737007	Abdominal pain in pregnancy	Physical illness	snomed
309774006	Weakness of limb	Physical illness	snomed
309784007	Blocked ureteric stent	Physical illness	snomed
309838005	Emotional upset	Mental health	snomed
30989003	Knee pain	Physical illness	snomed
310249008	Follow−up orthopaedic assessment	Other	snomed
310455000	Medical report requested	Other	snomed
310484009	C/O − pain in hallux	Physical illness	snomed
310495003	Mild depression	Mental health	snomed
310505005	Hyperosmolar non−ketotic state due to diabetes mellitus	Physical illness	snomed
31054009	Ureteric stone	Physical illness	snomed
31070006	Epididymitis	Physical illness	snomed
3110003	Acute otitis media	Physical illness	snomed
311453002	Pilonidal sinus of natal cleft	Physical illness	snomed
311821002	Closed fracture of great toe	Accident and injury	snomed
311822009	Open fracture of great toe	Accident and injury	snomed
312133006	Viral respiratory infection	Physical illness	snomed
312396008	Acetaminophen level normal	Other	snomed
312590001	Foreign body in lip	Accident and injury	snomed
312608009	Laceration	Accident and injury	snomed
312609001	Puncture wound − injury	Accident and injury	snomed
312887003	Attending clinic	Other	snomed
312984006	Abnormal uterine bleeding unrelated to menstrual cycle	Physical illness	snomed
313287004	Observations of seizure	Physical illness	snomed
313307000	Epileptic seizure	Physical illness	snomed

ed_code	description	presentation_category	classification
313331005	Admission for social reasons	Other	snomed
313341008	INR raised	Physical illness	snomed
313432002	Post−ictal drowsiness	Physical illness	snomed
3135009	Otitis externa	Physical illness	snomed
31399009	Abscess of scalp	Physical illness	snomed
314041007	Abdominal pain in early pregnancy	Physical illness	snomed
314204000	Early stage of pregnancy	Physical illness	snomed
314212008	Abdominal pain − cause unknown	Physical illness	snomed
31446002	Bipolar affective disorder, current episode hypomanic	Mental health	snomed
314483007	Primary post tonsillectomy haemorrhage	Physical illness	snomed
314484001	Secondary post tonsillectomy haemorrhage	Physical illness	snomed
314496008	Contact lens related disorder	Physical illness	snomed
314497004	Contact lens related corneal infiltrate	Physical illness	snomed
314499001	Contact lens related red eye	Physical illness	snomed
314503007	DNA − hospital appointment (finding)	Other	snomed
314506004	Traumatic corneal abrasion	Accident and injury	snomed
314516007	Preseptal cellulitis	Physical illness	snomed
314533000	Blunt injury of eye	Accident and injury	snomed
314534006	Thermal burn	Accident and injury	snomed
314550003	History of deliberate self harm	Suicidal behaviours and self−harm	snomed
314557000	Bacterial keratitis	Physical illness	snomed
31457007	Syncope on exertion	Physical illness	snomed
314630009	Abscess of labia	Physical illness	snomed
314716005	Acute pelvic pain	Physical illness	snomed
314828009	Epilepsy control poor	Physical illness	snomed
314871000	Prescription status	Other	snomed
314940005	Suspected urinary tract infection	Physical illness	snomed
314943007	Varicella contact	Physical illness	snomed
314984005	Lost prescription	Other	snomed
315018008	Dizzy spells	Physical illness	snomed
315224006	Heavy episode of vaginal bleeding	Physical illness	snomed
315597005	Abscess of upper limb	Physical illness	snomed
3163006	Acute adenoviral follicular conjunctivitis	Physical illness	snomed
31658008	Chronic paranoid schizophrenia	Mental health	snomed
31681005	Trigeminal neuralgia	Physical illness	snomed

ed_code	description	presentation_category	classification
31758001	Post−ictal state	Physical illness	snomed
31822004	Urethritis	Physical illness	snomed
31871009	Infection AND/OR inflammatory reaction due to internal prosthetic device, implant AND/OR graft	Physical illness	snomed
31928004	Abscess of skin and/or subcutaneous tissue	Physical illness	snomed
31956009	Cocaine dependence	Alcohol and substance misuse	snomed
31975004	Fracture of scaphoid bone	Accident and injury	snomed
31978002	Fracture of tibia	Accident and injury	snomed
31986002	Animal bite	Accident and injury	snomed
3199001	Sprain of shoulder	Accident and injury	snomed
31996006	Vasculitis	Physical illness	snomed
320934008	Oesophageal injury	Physical illness	snomed
32110003	Disorder of anus	Physical illness	snomed
3218000	Mycosis	Physical illness	snomed
32267003	Acute drug overdose	Alcohol and substance misuse	snomed
3228009	Closed fracture of shaft of radius	Accident and injury	snomed
3238004	Pericarditis	Physical illness	snomed
32390006	Panhypopituitarism	Physical illness	snomed
32398004	Bronchitis	Physical illness	snomed
324007	Plaster ulcer	Physical illness	snomed
3253007	Discolouration of skin	Physical illness	snomed
32553006	Hangover	Alcohol and substance misuse	snomed
32709003	Addiction	Alcohol and substance misuse	snomed
3282008	Welders' keratitis	Accident and injury	snomed
32861005	Erythema nodosum	Physical illness	snomed
32911000	Homeless	Other	snomed
32937002	Crisis	Mental health	snomed
32976001	Open wound of leg with complication	Accident and injury	snomed
330007	Occipital headache	Physical illness	snomed
33173003	Closed fracture of clavicle	Accident and injury	snomed
33192001	Closed fracture of lower end of radius AND ulna	Accident and injury	snomed
33334006	Foreign body in digestive tract	Accident and injury	snomed
33449004	Personality disorder	Mental health	snomed
33461007	Complication of peritoneal dialysis	Physical illness	snomed
33633005	Medicine prescription	Other	snomed
33659008	Keloid scar	Accident and injury	snomed

ed_code	description	presentation_category	classification
33678008	On examination − no disease present	Other	snomed
3368006	Dull chest pain	Physical illness	snomed
33737001	Fracture of rib	Accident and injury	snomed
33810006	Injection of eye proper (procedure)	Accident and injury	snomed
33826007	Epidermal burn of elbow	Accident and injury	snomed
33839006	Genital herpes simplex	Physical illness	snomed
33879002	Active immunisation	Other	snomed
33910007	Postoperative infection	Physical illness	snomed
33931005	Injury of lip	Accident and injury	snomed
33958003	Disorder of penis	Physical illness	snomed
33962009	Chief complaint	Other	snomed
34000006	Crohn's disease	Physical illness	snomed
34014006	Viral infection	Physical illness	snomed
34095006	Dehydration	Physical illness	snomed
34124000	Foreign body in vagina	Accident and injury	snomed
3415004	Cyanosis	Physical illness	snomed
342070009	Closed fracture of foot	Accident and injury	snomed
3424008	Tachycardia	Physical illness	snomed
34268009	Closed fracture of lateral malleolus	Accident and injury	snomed
34436003	Haematuria	Physical illness	snomed
34486009	Hyperthyroidism	Physical illness	snomed
34622000	Temporomandibular subluxation	Physical illness	snomed
34663006	Contusion of brain	Accident and injury	snomed
34789001	Pain in the coccyx	Physical illness	snomed
3480002	Burn of wrist	Accident and injury	snomed
34801009	Ectopic pregnancy	Physical illness	snomed
34840004	Tendinitis	Accident and injury	snomed
34842007	Antepartum haemorrhage	Physical illness	snomed
3502005	Cervical lymphadenitis	Physical illness	snomed
35037009	Primary atypical interstitial pneumonia	Physical illness	snomed
3507004	Abscess of thigh	Physical illness	snomed
35265002	Mallory−Weiss syndrome	Physical illness	snomed
352818000	Tonic−clonic epilepsy	Physical illness	snomed
35298007	Slow transit constipation	Physical illness	snomed
3545003	Diastolic dysfunction	Physical illness	snomed

ed_code	description	presentation_category	classification
35489007	Depression	Mental health	snomed
35678005	Arthralgia of multiple joints	Physical illness	snomed
35688006	Complication of medical care	Physical illness	snomed
35708002	Abscess of perineum	Physical illness	snomed
35745008	Umbilical discharge	Physical illness	snomed
3577000	Retinal lattice degeneration	Physical illness	snomed
35868009	Carcinoid syndrome	Physical illness	snomed
35923002	Chronic maxillary sinusitis	Physical illness	snomed
35933005	Tear − wound	Accident and injury	snomed
359751003	Impetigo caused by Staphylococcus aureus	Physical illness	snomed
360371003	Acute cardiac pulmonary oedema	Physical illness	snomed
360437006	Strain of tendon of neck	Accident and injury	snomed
360450007	Strain of neck muscle	Accident and injury	snomed
361055000	Substance abuser	Alcohol and substance misuse	snomed
361136003	Abnormal heart beat	Physical illness	snomed
36118008	Pneumothorax	Physical illness	snomed
36202009	Fracture of tooth	Accident and injury	snomed
36281004	Abscess of wrist	Physical illness	snomed
362969004	Disorder of endocrine system	Physical illness	snomed
363101005	Drug withdrawal	Alcohol and substance misuse	snomed
36311007	Oedema of vulva	Physical illness	snomed
363162000	Infectious disorder of joint	Physical illness	snomed
3633001	Abscess of hand	Physical illness	snomed
363732003	Addison's disease	Physical illness	snomed
363746003	Acute pharyngitis	Physical illness	snomed
36427004	Intervertebral disc disorder	Physical illness	snomed
36456004	Mental state finding	Mental health	snomed
365747005	Urine drug levels − finding	Alcohol and substance misuse	snomed
366661000	Hair characteristics − finding	Alcohol and substance misuse	snomed
36689008	Acute pyelonephritis	Physical illness	snomed
366979004	Depressed mood	Mental health	snomed
36715001	Erythema multiforme	Physical illness	snomed
36734001	Catatonic posturing	Mental health	snomed
367391008	Malaise	Physical illness	snomed
367409002	FB	Accident and injury	snomed

ed_code	description	presentation_category	classification
367423000	Contusion of eye	Accident and injury	snomed
36787001	Contusion of trunk	Accident and injury	snomed
36789003	Acute infective gastroenteritis	Physical illness	snomed
36857002	Onychia of finger	Physical illness	snomed
36924003	Closed fracture of metatarsal bone	Accident and injury	snomed
36948007	Fasciitis	Physical illness	snomed
36971009	Sinusitis	Physical illness	snomed
36989005	Mumps	Physical illness	snomed
36991002	Closed fracture of upper limb	Accident and injury	snomed
36994005	Transient visual loss	Physical illness	snomed
370143000	Major depressive disorder	Mental health	snomed
370218001	Mild asthma	Physical illness	snomed
370239008	Superficial laceration of toe	Accident and injury	snomed
370240005	Superficial laceration of foot	Accident and injury	snomed
370242002	Superficial laceration of chest wall	Accident and injury	snomed
370243007	Superficial laceration of finger	Accident and injury	snomed
370244001	Superficial laceration of hand	Accident and injury	snomed
370245000	Superficial laceration of thumb	Accident and injury	snomed
370246004	Superficial laceration of upper limb	Accident and injury	snomed
370247008	Facial laceration	Accident and injury	snomed
370506009	Keratouveitis	Physical illness	snomed
370509002	Neurapraxia	Physical illness	snomed
37057007	Psychophysiologic disorder	Mental health	snomed
370773004	Administration of prescribed medications and solutions	Other	snomed
370956008	Corneal epithelial defect	Physical illness	snomed
37098000	Cellulitis of external nose	Physical illness	snomed
37102008	Ovarian dysfunction	Physical illness	snomed
371039008	Thromboembolic disorder	Physical illness	snomed
371073003	Postural orthostatic tachycardia syndrome	Physical illness	snomed
371077002	Ventriculoperitoneal shunt malfunction	Physical illness	snomed
371087003	Diabetic foot ulcer	Physical illness	snomed
371405004	Disorder of eye	Physical illness	snomed
371409005	Disorder of eye region	Physical illness	snomed
37156001	Disorder of jaw	Physical illness	snomed
371596008	Bipolar I disorder	Mental health	snomed

ed_code	description	presentation_category	classification
371600003	Severe bipolar disorder	Mental health	snomed
371631005	Panic disorder	Mental health	snomed
371704001	Injury due to chemical exposure	Accident and injury	snomed
371708003	Injury due to electrical exposure	Accident and injury	snomed
37174005	Closed fracture of hamate bone of wrist	Accident and injury	snomed
37223007	Cellulitis of neck	Physical illness	snomed
37224001	Psychogenic vomiting	Mental health	snomed
3723001	Arthritis	Physical illness	snomed
372871004	Promethazine (substance)	Other	snomed
372939007	Suppurative arthritis	Physical illness	snomed
373117000	Pathology examination findngs indeterminate (finding)	Other	snomed
37344009	Cannabis abuse	Alcohol and substance misuse	snomed
373602003	Laceration of nose	Accident and injury	snomed
37372002	Upper gastrointestinal haemorrhage	Physical illness	snomed
37389005	Biliary colic	Physical illness	snomed
373931001	Sensation of heaviness in limbs	Physical illness	snomed
373945007	Pericardial effusion	Physical illness	snomed
37418005	Multiple fractures of hand bones	Accident and injury	snomed
37450000	Corneal foreign body	Accident and injury	snomed
37610005	Inflammation of cervix	Physical illness	snomed
37785001	Patellar tendinitis	Physical illness	snomed
37796009	Migraine	Physical illness	snomed
37871000	Acute hepatitis	Physical illness	snomed
37907001	Contusion of abdominal wall	Accident and injury	snomed
38013005	Immunosuppression	Physical illness	snomed
38059007	Prolapsed external haemorrhoids	Physical illness	snomed
38301007	Closed traumatic dislocation of joint of thumb	Accident and injury	snomed
38341003	Hypertension	Physical illness	snomed
38343000	Vaginal pain	Physical illness	snomed
38354005	Open wound of head	Accident and injury	snomed
38362002	Dengue fever	Physical illness	snomed
38394007	Chronic otitis media with perforation	Physical illness	snomed
38433004	No sensitivity to pain	Other	snomed
384709000	Sprain (morphologic abnormality)	Accident and injury	snomed
38484003	Abrasion and/or friction burn of back without infection	Accident and injury	snomed

ed_code	description	presentation_category	classification
385093006	Community acquired pneumonia	Physical illness	snomed
385486001	Postoperative complication	Physical illness	snomed
385494008	Haematoma	Accident and injury	snomed
38556006	Closed traumatic dislocation of joint of wrist	Accident and injury	snomed
385627004	Cellulitis (morphologic abnormality)	Physical illness	snomed
385893007	Mental health treatment (regime/therapy)	Mental health	snomed
385942004	Wound care management	Accident and injury	snomed
386034005	Acute mastoiditis	Physical illness	snomed
386053000	Evaluation procedure	Other	snomed
386661006	Fever	Physical illness	snomed
386663009	Bicycle accident	Accident and injury	snomed
386689009	Hypothermia	Physical illness	snomed
386692008	Menorrhagia	Physical illness	snomed
386702006	Victim of abuse	Accident and injury	snomed
386705008	Lightheadedness	Physical illness	snomed
386738004	Multiple somatic complaints	Physical illness	snomed
386804004	Disorder of menstruation	Physical illness	snomed
386807006	Memory impairment	Mental health	snomed
386813002	Abnormal breathing	Physical illness	snomed
386816005	Emotional problems	Mental health	snomed
386817001	Behaviour problem of childhood and adolescence	Mental health	snomed
387665005	Pulpal abscess	Physical illness	snomed
38822007	Cystitis	Physical illness	snomed
388983002	Paronychia of toe	Physical illness	snomed
38907003	Varicella	Physical illness	snomed
389145006	Allergic asthma	Physical illness	snomed
3895009	Application of dressing	Other	snomed
390808007	Mental health care	Mental health	snomed
390809004	Emergency mental health assessment	Mental health	snomed
390811008	Crisis/short term interventions in mental health care (regime/therapy)	Mental health	snomed
391281002	Mental health assessment	Mental health	snomed
391906003	Closure of skin by suture (procedure)	Accident and injury	snomed
39402007	Pelvic congestion syndrome	Physical illness	snomed
39406005	Legal termination of pregnancy	Physical illness	snomed
394587001	Psychiatry (qualifier value)	Mental health	snomed

ed_code	description	presentation_category	classification
394616008	Unsteady gait (finding)	Physical illness	snomed
394642008	Drug screening test (procedure)	Alcohol and substance misuse	snomed
394659003	Acute coronary syndrome	Physical illness	snomed
394686003	Moderate suicide risk	Suicidal behaviours and self−harm	snomed
394924000	Symptoms of depression	Mental health	snomed
395017009	C/O − panic attack	Mental health	snomed
395080004	Sensory disturbance in limb	Physical illness	snomed
395101001	Ongoing review	Other	snomed
39579001	Anaphylaxis	Physical illness	snomed
396073008	Medicine administration assessment	Other	snomed
39621005	Gallbladder disorder	Physical illness	snomed
396232000	Inguinal hernia	Physical illness	snomed
396285007	Bronchopneumonia	Physical illness	snomed
396331005	Coeliac disease	Physical illness	snomed
396332003	Rheumatism	Physical illness	snomed
396337009	Acute and chronic gastritis	Physical illness	snomed
396347007	Umbilical hernia	Physical illness	snomed
396348002	Acute eczema	Physical illness	snomed
396544001	Caesarean wound disruption	Physical illness	snomed
396550006	Blood test	Other	snomed
397683000	Pseudomembranous enterocolitis (disorder)	Physical illness	snomed
397696004	Antibiotic enterocolitis	Physical illness	snomed
397712006	Absence of signs and symptoms of electrical injury (context−dependent category)	Accident and injury	snomed
397828008	Median neuropathy	Physical illness	snomed
397869004	Dental trauma	Accident and injury	snomed
397940009	Victim of child abuse	Accident and injury	snomed
398057008	Tension headache	Physical illness	snomed
398117008	Falling injury	Accident and injury	snomed
39812007	Contusion of forearm	Accident and injury	snomed
398126006	Muscular headache	Physical illness	snomed
398232005	Drug dose (finding)	Other	snomed
398254007	Pre−eclampsia	Physical illness	snomed
39848009	Whiplash injury to neck	Accident and injury	snomed
398652001	Vasovagal attack (disorder)	Physical illness	snomed
398665005	Vasovagal syncope	Physical illness	snomed

ed_code	description	presentation_category	classification
398870000	Recurrent aphthous ulcer	Physical illness	snomed
398909004	Rosacea	Physical illness	snomed
398987004	Headache following lumbar puncture	Physical illness	snomed
399029005	Tinea cruris	Physical illness	snomed
399114005	Adhesive capsulitis of shoulder	Physical illness	snomed
399131003	Non−menstrual vaginal bleeding	Physical illness	snomed
399153001	Vertigo	Physical illness	snomed
399221001	Bleeding from vagina (disorder)	Physical illness	snomed
399858007	Retina finding	Physical illness	snomed
399907009	Animal bite wound	Accident and injury	snomed
399912005	Pressure injury	Accident and injury	snomed
399963005	Abrasion	Accident and injury	snomed
400012003	Abrasion and/or friction burn of skin	Accident and injury	snomed
400045003	Superficial laceration of skin	Accident and injury	snomed
400097005	Ingrowing nail	Physical illness	snomed
400200009	Ingrowing toenail	Physical illness	snomed
40024006	Retinal defect	Physical illness	snomed
40055000	Chronic sinusitis	Physical illness	snomed
4009004	Lower urinary tract infectious disease	Physical illness	snomed
40095003	Renal injury	Physical illness	snomed
400976006	Head injury advice given	Accident and injury	snomed
400978007	Drug compliance poor	Other	snomed
400990009	Blanching rash	Physical illness	snomed
401061005	Mental health review	Mental health	snomed
401141001	Parental concern about child	Other	snomed
401206008	At risk for deliberate self harm	Suicidal behaviours and self−harm	snomed
401207004	Medicine side effects present	Other	snomed
401229000	Thoughts of deliberate self harm	Suicidal behaviours and self−harm	snomed
401232002	Unknown risk of deliberate self harm	Suicidal behaviours and self−harm	snomed
401303003	Acute ST segment elevation myocardial infarction	Physical illness	snomed
401314000	Acute non−ST segment elevation myocardial infarction	Physical illness	snomed
40178009	Allergic urticaria	Physical illness	snomed
402121009	Epstein−Barr virus infection (disorder)	Physical illness	snomed
402192007	Atopic dermatitis of face	Physical illness	snomed
402294001	Chapping of lips	Other	snomed

ed_code	description	presentation_category	classification
402387002	Allergic angioedema	Physical illness	snomed
402408009	Acute urticaria	Physical illness	snomed
40257000	Contusion of shoulder region	Accident and injury	snomed
40275004	Contact dermatitis	Physical illness	snomed
40283005	Thrombophlebitis of superficial veins of lower limb	Physical illness	snomed
402894005	Recurrent genital herpes simplex	Physical illness	snomed
402938009	Staphylococcal infection of skin	Physical illness	snomed
403149008	Spider bite wound	Accident and injury	snomed
403190006	Epidermal burn of skin	Accident and injury	snomed
403191005	Partial thickness burn	Accident and injury	snomed
403398009	Cutis laxa following urticaria−angioedema	Physical illness	snomed
403465000	Chronic infective balanitis	Physical illness	snomed
403583006	Deliberate self−cutting	Suicidal behaviours and self−harm	snomed
404189009	Domestic violence	Accident and injury	snomed
404223003	Deep venous thrombosis of lower limb	Physical illness	snomed
40425004	Post−concussion syndrome	Physical illness	snomed
40458008	Abscess of chest wall	Physical illness	snomed
404640003	Dizziness	Physical illness	snomed
405275001	Rupture of ulnar collateral ligament of thumb	Accident and injury	snomed
40541001	Acute pulmonary oedema	Physical illness	snomed
405571006	Electrical burn	Accident and injury	snomed
405729008	Haematochezia	Physical illness	snomed
405737000	Pharyngitis	Physical illness	snomed
405783006	Psychological assessment (procedure)	Mental health	snomed
405944004	Asthmatic bronchitis	Physical illness	snomed
40613008	Open fracture of nasal bones	Accident and injury	snomed
406506008	Attention deficit hyperactivity disorder	Mental health	snomed
406554000	Low risk of harm to self	Suicidal behaviours and self−harm	snomed
40733004	Infection	Physical illness	snomed
40739000	Dysphagia	Physical illness	snomed
407560009	At risk of sexually transmitted infection	Physical illness	snomed
407622005	Many seizures a day	Physical illness	snomed
408099007	Suture (physical object)	Accident and injury	snomed
40835002	Coffee ground vomiting	Physical illness	snomed
408366001	Unable to use medication (finding)	Other	snomed

ed_code	description	presentation_category	classification
408536007	Clozapine monitoring (finding)	Mental health	snomed
4088009	Acquired hydrocephalus	Accident and injury	snomed
408856003	Autistic disorder	Mental health	snomed
40890009	Oesophageal dysphagia	Physical illness	snomed
409021006	Dispensing medication education	Other	snomed
409089005	Febrile neutropenia	Physical illness	snomed
40917007	Confusion	Physical illness	snomed
40930008	Hypothyroidism	Physical illness	snomed
409516001	Post−exposure prophylaxis (procedure)	Physical illness	snomed
40959008	Acute viral pericarditis	Physical illness	snomed
409596002	Non−productive cough (finding)	Physical illness	snomed
409630004	Asymptomatic viraemia	Physical illness	snomed
409631000	Acute viral disease	Physical illness	snomed
40970001	Chronic osteomyelitis	Physical illness	snomed
409780002	Acute osteomyelitis	Physical illness	snomed
409966000	Acute diarrhoea	Physical illness	snomed
41006004	Depression	Mental health	snomed
410061008	Intentional poisoning	Suicidal behaviours and self−harm	snomed
410062001	Laceration of vagina	Accident and injury	snomed
410223002	Mental health care assessment	Mental health	snomed
410229003	Mental health screening assessment	Mental health	snomed
410232000	Mental health treatment assessment	Mental health	snomed
410234004	Management of mental health treatment	Mental health	snomed
4103001	Complex partial seizure with impairment of consciousness	Physical illness	snomed
41036008	Closed fracture of styloid process of ulna	Accident and injury	snomed
410429000	Cardiac arrest	Physical illness	snomed
410543007	No show	Other	snomed
4106009	Rotator cuff syndrome	Physical illness	snomed
410692006	Anterior uveitis	Physical illness	snomed
410706007	Stabbing sensation quality (qualifier value)	Physical illness	snomed
410709000	Cramping sensation quality (qualifier value)	Physical illness	snomed
410713007	Sore sensation quality (qualifier value)	Physical illness	snomed
41291007	Angioedema	Physical illness	snomed
41308008	Acute follicular conjunctivitis	Physical illness	snomed
413090001	Referral letter status	Other	snomed

ed_code	description	presentation_category	classification
413235002	PEG externally removable	Physical illness	snomed
41327002	Police investigation	Other	snomed
413306008	Informal referral − signposted to other agency (finding)	Other	snomed
413307004	Mental health problem	Mental health	snomed
413875004	Closed fracture of head of humerus	Accident and injury	snomed
413877007	Closed fracture of tibia AND fibula	Accident and injury	snomed
413878002	Closed, displaced fracture of nasal bone	Accident and injury	snomed
413897002	Community detoxification registered (finding)	Alcohol and substance misuse	snomed
413939000	Cystocele with second degree uterine prolapse	Physical illness	snomed
414029004	Disorder of immune function	Physical illness	snomed
414033006	Disorder of rotator cuff	Physical illness	snomed
414188008	Fall down stairs	Accident and injury	snomed
414189000	Fall down steps	Accident and injury	snomed
414285001	Food allergy	Physical illness	snomed
414292006	Fracture of lower leg	Accident and injury	snomed
414293001	Fracture of tibia AND fibula	Accident and injury	snomed
41446000	Blepharitis	Physical illness	snomed
414474001	Ventral incisional hernia	Physical illness	snomed
414521009	Internal hordeolum	Physical illness	snomed
414545008	Ischaemic heart disease	Physical illness	snomed
414943006	Open fracture of tibia AND fibula	Accident and injury	snomed
414992000	Painless rectal bleeding	Physical illness	snomed
41501003	Threatening suicide	Suicidal behaviours and self−harm	snomed
41511005	Open fracture of distal phalanx of finger	Accident and injury	snomed
415172002	Primary acquired melanocytic naevus of conjunctiva	Physical illness	snomed
415175000	Primary acquired melanosis of conjunctiva without atypia	Physical illness	snomed
415297005	Retinopathy of prematurity	Physical illness	snomed
415658005	Substance misuse behaviour	Alcohol and substance misuse	snomed
41582007	Streptococcal tonsillitis	Physical illness	snomed
41608004	Closed fractures of tarsal AND metatarsal bones	Accident and injury	snomed
416093006	Allergic reaction to drug	Physical illness	snomed
416113008	Febrile illness	Physical illness	snomed
416302000	Rapid referral from minor ailments clinic (finding)	Physical illness	snomed
416381005	Prescription collected	Other	snomed
416462003	Wound	Accident and injury	snomed

ed_code	description	presentation_category	classification
41652007	Eye pain	Physical illness	snomed
416675009	Furuncle	Physical illness	snomed
416886008	Closed wound	Accident and injury	snomed
416960004	Corneal endothelial dystrophy	Physical illness	snomed
417076003	Dislocation of shoulder joint	Accident and injury	snomed
417096006	Referral to community drug and alcohol team (procedure)	Alcohol and substance misuse	snomed
417163006	Traumatic AND/OR non−traumatic injury	Accident and injury	snomed
417233008	Paranoid ideation	Mental health	snomed
417284009	Current drug user	Alcohol and substance misuse	snomed
417350008	Altered behaviour	Mental health	snomed
417373000	Inflammatory polyarthropathy	Physical illness	snomed
417473004	O/E − decreased level of consciousness	Physical illness	snomed
417532002	Allergy to fish	Physical illness	snomed
417654008	Contusion of ocular adnexa and periocular tissues	Accident and injury	snomed
417713005	Abnormality of surgical wound (disorder)	Other	snomed
417746004	Traumatic injury	Accident and injury	snomed
417981005	Exposure to blood and/or body fluid	Physical illness	snomed
418107008	Unconscious	Physical illness	snomed
418290006	Itching	Physical illness	snomed
418363000	Itching of skin	Physical illness	snomed
418399005	Motor vehicle accident	Accident and injury	snomed
418420002	Intentionally harming self	Suicidal behaviours and self−harm	snomed
418634005	Allergic reaction to substance	Physical illness	snomed
418809008	Allergic reaction caused by dye	Physical illness	snomed
418842009	Disorder of vitreous cavity	Physical illness	snomed
41888000	Temporomandibular joint disorder	Physical illness	snomed
418925002	Immune hypersensitivity reaction (disorder)	Physical illness	snomed
419045004	Loss of consciousness	Physical illness	snomed
41906002	Drug withdrawal seizure	Alcohol and substance misuse	snomed
419076005	Allergic reaction	Physical illness	snomed
419219000	Drug−induced nausea and vomiting	Physical illness	snomed
419284004	Altered mental state	Mental health	snomed
41931001	Abdominal distension	Physical illness	snomed
419452009	Allergic reaction to food	Physical illness	snomed
419502003	Chest infection (disorder)	Physical illness	snomed

ed_code	description	presentation_category	classification
419603000	Epidermoid cyst of skin	Physical illness	snomed
420103007	Watery eye	Physical illness	snomed
420422005	Ketoacidosis due to diabetes mellitus	Physical illness	snomed
42059000	Retinal detachment	Physical illness	snomed
42063007	Sign or symptom of the urinary system	Physical illness	snomed
420904002	Lacrimal gland finding	Physical illness	snomed
420999000	Vitreous debris	Physical illness	snomed
42131003	Abnormal menstrual cycle	Physical illness	snomed
42134006	Metamorphopsia	Mental health	snomed
421512008	Nonspecific exanthematous viral infection (disorder)	Physical illness	snomed
421750000	Ketoacidosis due to type 2 diabetes mellitus	Physical illness	snomed
421843005	Subretinal fluid	Physical illness	snomed
42188001	Closed fracture of ankle	Accident and injury	snomed
422183001	Diabetic skin ulcer	Physical illness	snomed
422400008	Vomiting	Physical illness	snomed
4224004	Complication of infusion	Physical illness	snomed
422411000	Surgical scar	Physical illness	snomed
422426003	Disorder of facial nerve	Physical illness	snomed
422504002	Ischaemic stroke	Physical illness	snomed
422587007	Nausea	Physical illness	snomed
422588002	Aspiration pneumonia	Physical illness	snomed
422608009	Sexual assault (finding)	Accident and injury	snomed
422768004	Unresponsive	Physical illness	snomed
422868009	Unexplained weight loss	Physical illness	snomed
422916003	Blunt injury of thorax	Accident and injury	snomed
422947009	Corneal subepithelial scar	Physical illness	snomed
423051001	Haematoma of face	Accident and injury	snomed
423234004	Inhalation injury	Accident and injury	snomed
42343007	Congestive heart failure	Physical illness	snomed
42344001	Alcohol−induced psychosis	Mental health	snomed
423488006	Papilloedema − optic disc oedema due to raised intracranial pressure	Physical illness	snomed
423716004	Petechiae of skin	Physical illness	snomed
423778009	Tenosynovitis of hand	Physical illness	snomed
423810002	Tendinitis of wrist	Accident and injury	snomed
423902002	Purpura	Physical illness	snomed

ed_code	description	presentation_category	classification
42399005	Renal failure	Physical illness	snomed
424131007	Easy bruising	Physical illness	snomed
424316008	Burn by hot liquid	Accident and injury	snomed
424461008	Behaviour change due to substance use	Mental health	snomed
424685008	Bite wound of skin	Accident and injury	snomed
424863004	Blunt injury of abdomen	Accident and injury	snomed
425104003	Suicidal behaviour	Suicidal behaviours and self−harm	snomed
425144005	Minor open wound	Accident and injury	snomed
425322008	Stab wound	Accident and injury	snomed
425359009	Blunt injury	Accident and injury	snomed
425492002	Generalised dystonia	Physical illness	snomed
425772008	Tendinitis of foot	Accident and injury	snomed
425860006	Pain radiating to lower abdomen	Physical illness	snomed
425940002	Olecranon bursitis	Accident and injury	snomed
426032000	Malfunction of gastrostomy tube	Physical illness	snomed
426284001	Chemical burn	Accident and injury	snomed
42636007	Closed fracture of upper end of humerus	Accident and injury	snomed
426396005	Cardiac chest pain	Physical illness	snomed
42643001	Disorder of bladder	Physical illness	snomed
426469008	Pain radiating to left leg	Physical illness	snomed
42658009	Disorder of the peripheral nervous system	Physical illness	snomed
426912003	Splinter foreign body (morphologic abnormality)	Accident and injury	snomed
426936004	Smoke inhalation injury	Accident and injury	snomed
426965005	Aphthous ulcer of mouth	Physical illness	snomed
427310006	Pain radiating to neck	Physical illness	snomed
427461000	Presyncope	Physical illness	snomed
427509002	Closed Salter−Harris type I fracture of lower epiphysis of femur	Accident and injury	snomed
427653003	Pain radiating to right leg	Physical illness	snomed
427679007	Mild intermittent asthma	Physical illness	snomed
427769002	Infection of amputation stump	Physical illness	snomed
427793007	Complication of urinary catheter	Physical illness	snomed
427898007	Infection of tooth	Physical illness	snomed
428088000	Laceration of head	Accident and injury	snomed
428099003	Closed fracture of base of skull	Accident and injury	snomed
428152007	Penetrating wound of neck	Accident and injury	snomed

ed_code	description	presentation_category	classification
42818005	Closed fracture of scaphoid bone of wrist	Accident and injury	snomed
428200002	Assault by person	Accident and injury	snomed
428324008	Piercing of tongue (procedure)	Accident and injury	snomed
428438000	Inhalation injury due to chemical	Accident and injury	snomed
428786006	Localised superficial swelling of skin	Physical illness	snomed
428819003	Opiate misuse	Alcohol and substance misuse	snomed
428823006	Cannabis misuse	Alcohol and substance misuse	snomed
429093006	Abnormal neurovascular status of distal limb	Physical illness	snomed
429196001	Partial obstruction of small bowel	Physical illness	snomed
42942008	Compression fracture of spine	Accident and injury	snomed
429433004	Injury of soft tissue of face	Accident and injury	snomed
429530004	Dizziness of unknown cause	Physical illness	snomed
429562001	Abrasion and/or friction burn of multiple sites	Accident and injury	snomed
429655000	Closed torus fracture of radius	Accident and injury	snomed
429696002	Instability of patellofemoral joint	Physical illness	snomed
429719000	Foreign body in forearm	Accident and injury	snomed
43077002	Epididymal cyst	Physical illness	snomed
430909002	Conduct disorder	Mental health	snomed
430981001	Avulsion injury of fingernail	Accident and injury	snomed
430984009	Closed fracture of facial bone	Accident and injury	snomed
43116000	Eczema	Physical illness	snomed
431237007	Chronic headache disorder	Physical illness	snomed
431307001	Intentional drug poisoning	Suicidal behaviours and self−harm	snomed
431309003	Acute urinary tract infection	Physical illness	snomed
431737008	Acute lower urinary tract infection	Physical illness	snomed
431957001	Seen in mental health clinic (finding)	Mental health	snomed
43240000	Diarrhoea of presumed infectious origin	Physical illness	snomed
432615008	Chronic pain in face	Physical illness	snomed
432618005	Penetrating wound of lower limb	Accident and injury	snomed
432754003	Injury of sternum	Accident and injury	snomed
43295006	Closed fracture of humerus	Accident and injury	snomed
43339004	Hypokalaemia	Physical illness	snomed
43364001	Abdominal discomfort	Physical illness	snomed
43422002	Crushing injury of foot	Accident and injury	snomed
43478001	Abdominal tenderness	Physical illness	snomed

ed_code	description	presentation_category	classification
43491000	Acute epididymitis	Physical illness	snomed
43548008	Mittelschmerz	Physical illness	snomed
438457000	Swelling of testicle	Physical illness	snomed
438479005	Injury of ligament of knee	Accident and injury	snomed
438480008	Injury of nail bed of finger	Accident and injury	snomed
438582003	Acute injury of anterior cruciate ligament	Physical illness	snomed
4386001	Bronchospasm	Physical illness	snomed
43878008	Streptococcal sore throat	Physical illness	snomed
439022003	Traumatic injury due to assault	Accident and injury	snomed
439377004	Accidental removal of catheter	Accident and injury	snomed
439469002	Recurrent abdominal pain	Physical illness	snomed
440144004	Injury due to suicide attempt	Suicidal behaviours and self−harm	snomed
440311000	Suprapubic urinary catheter in situ	Physical illness	snomed
44037003	Globus pharyngeus	Physical illness	snomed
440402001	Short term psychiatric in−patient (finding)	Mental health	snomed
44077006	Numbness	Physical illness	snomed
44132006	Abscess morphology	Physical illness	snomed
441457006	Cyst	Physical illness	snomed
441668002	Drug seeking behaviour	Alcohol and substance misuse	snomed
441704009	Affective psychosis	Mental health	snomed
441708007	Swelling of lower jaw region	Physical illness	snomed
44186003	Dyssomnia	Physical illness	snomed
442025000	Acute exacerbation of chronic asthmatic bronchitis	Physical illness	snomed
44216000	Postpartum haemorrhage with retained placenta	Physical illness	snomed
44256003	Chronic eczema	Physical illness	snomed
442618008	Abnormal finding on evaluation procedure	Physical illness	snomed
442696006	Influenza caused by Influenza A virus subtype H1N1	Physical illness	snomed
443137009	Infection of pierced pinna	Physical illness	snomed
443371007	Decreased level of consciousness	Physical illness	snomed
44376007	Dissociative disorder	Mental health	snomed
443769009	Abscess of female genital structure	Physical illness	snomed
443920001	Simple laceration	Accident and injury	snomed
443999008	Risk of exposure to communicable disease	Physical illness	snomed
444019000	Did not wait for treatment	Other	snomed
44402007	Loss of teeth due to extraction	Physical illness	snomed

ed_code	description	presentation_category	classification
444158007	Injury of cruciate ligament of knee	Accident and injury	snomed
444159004	Injury of collateral ligament of knee	Accident and injury	snomed
44428005	Cellulitis of buttock	Physical illness	snomed
44465007	Sprain of ankle	Accident and injury	snomed
444673007	Hyperemesis	Physical illness	snomed
445060000	Left against medical advice	Other	snomed
445095002	Witnessed epileptic seizure	Physical illness	snomed
445410003	Closed fracture of distal tibia and distal fibula	Accident and injury	snomed
44612009	Structure of sternal angle	Physical illness	snomed
446456003	Deep laceration of thumb	Accident and injury	snomed
446653004	Foreign body in lower limb	Accident and injury	snomed
446869009	Superficial laceration of knee	Accident and injury	snomed
447139008	Closed fracture of tibia	Accident and injury	snomed
44730006	Uraemia	Physical illness	snomed
4473006	Migraine with aura	Physical illness	snomed
447350003	Laceration of eyebrow	Accident and injury	snomed
447395005	Closed fracture of fibula	Accident and injury	snomed
44782008	Molar pregnancy	Physical illness	snomed
44801007	Contusion of hip	Accident and injury	snomed
44808001	Conduction disorder of the heart	Physical illness	snomed
449671007	Cellulitis of upper limb	Physical illness	snomed
449710006	Cellulitis of lower limb	Physical illness	snomed
45007003	Hypotension	Physical illness	snomed
4506002	Educational problem	Other	snomed
45082006	Acute orchitis	Physical illness	snomed
45150006	Auditory hallucinations	Mental health	snomed
45170000	Encephalitis	Physical illness	snomed
45177002	Eye swelling	Physical illness	snomed
45198002	Mastitis (disorder)	Physical illness	snomed
45261009	Viral conjunctivitis	Physical illness	snomed
45326000	Shoulder pain	Physical illness	snomed
45352006	Spasm	Physical illness	snomed
4556007	Gastritis	Physical illness	snomed
45613006	Contusion of lower leg	Accident and injury	snomed
45775001	Amphetamine poisoning	Alcohol and substance misuse	snomed

ed_code	description	presentation_category	classification
45816000	Pyelonephritis	Physical illness	snomed
45913009	Laryngitis	Physical illness	snomed
45959006	Acute suppurative inflammation	Physical illness	snomed
46153004	Catatonic stupor	Mental health	snomed
46206005	Mood disorder	Mental health	snomed
46422008	Closed fracture of shaft of metacarpal bone	Accident and injury	snomed
46635009	Type 1 diabetes mellitus	Physical illness	snomed
46689006	Hypertrophy of tonsils	Physical illness	snomed
46742003	Skin ulcer	Physical illness	snomed
46866001	Fracture of lower limb	Accident and injury	snomed
46871008	Scrotal varices − varicocele	Physical illness	snomed
46960006	Lumbago−sciatica due to displacement of lumbar intervertebral disc	Physical illness	snomed
46970008	Mycoplasma pneumonia	Physical illness	snomed
47032000	Congenital hydrocephalus	Physical illness	snomed
47117005	Superficial injury of upper arm with infection	Accident and injury	snomed
47268002	Reflux (finding)	Physical illness	snomed
47372000	Adjustment disorder with anxious mood	Mental health	snomed
47382004	Dermatophytosis	Physical illness	snomed
47398006	Marginal corneal ulcer	Physical illness	snomed
4740000	Herpes zoster	Physical illness	snomed
47505003	Post−traumatic stress disorder	Mental health	snomed
4754008	Gynaecomastia	Physical illness	snomed
47609003	Foreign body in oesophagus	Accident and injury	snomed
47639008	Pilonidal cyst	Physical illness	snomed
47695004	Inability to cope	Mental health	snomed
47717004	Abnormal swallowing	Physical illness	snomed
4776004	Lichen planus	Physical illness	snomed
47821001	Postpartum haemorrhage	Physical illness	snomed
47874006	Sprain of arm	Accident and injury	snomed
4788002	Closed fracture of zygoma	Accident and injury	snomed
47933007	Foot pain	Physical illness	snomed
48123002	Contusion of wrist	Accident and injury	snomed
48167000	Amnesia	Mental health	snomed
4821001	Contusion of cheek	Accident and injury	snomed
48245008	Septic arthritis	Physical illness	snomed

ed_code	description	presentation_category	classification
48277006	Impetigo	Physical illness	snomed
48333001	Burn − lesion	Accident and injury	snomed
48440001	Arthritis due to gout	Physical illness	snomed
48500005	Delusional disorder	Mental health	snomed
48532005	Muscle strain	Accident and injury	snomed
48661000	Peritonitis	Physical illness	snomed
48694002	Anxiety	Mental health	snomed
48867003	Bradycardia	Physical illness	snomed
49130001	Disorder of external ear	Physical illness	snomed
49218002	Hip pain	Physical illness	snomed
4927003	Acute anterior uveitis	Physical illness	snomed
49298003	Abscess of trunk	Physical illness	snomed
49346003	Closed fracture of orbital floor (blow−out)	Accident and injury	snomed
49388007	Sprain of foot	Accident and injury	snomed
49436004	Atrial fibrillation	Physical illness	snomed
49631001	Acute mesenteric adenitis	Physical illness	snomed
49650001	Dysuria	Physical illness	snomed
4969004	Sinus headache	Physical illness	snomed
49723003	Intussusception of intestine	Physical illness	snomed
49727002	Cough	Physical illness	snomed
49810002	Cellulitis of shoulder	Physical illness	snomed
49882001	Viral exanthem	Physical illness	snomed
50063009	Femoral hernia	Physical illness	snomed
50257008	Perforation of colon	Physical illness	snomed
50320000	Hallucinogen intoxication	Alcohol and substance misuse	snomed
50397009	Closed fracture of distal end of ulna	Accident and injury	snomed
50417007	Lower respiratory tract infection	Physical illness	snomed
50438001	Peripheral vertigo	Physical illness	snomed
50446000	Monocular diplopia	Physical illness	snomed
50448004	Fracture of vertebral column	Accident and injury	snomed
50793006	Crushing injury of hand	Accident and injury	snomed
50866000	Childhood absence epilepsy	Physical illness	snomed
50920009	Myocarditis	Physical illness	snomed
50960005	Haemorrhage	Physical illness	snomed
51030006	Cellulitis of lip	Physical illness	snomed

ed_code	description	presentation_category	classification
51037009	Fracture of patella	Accident and injury	snomed
51299004	Collar bone	Physical illness	snomed
51551000	Bleeding haemorrhoids	Physical illness	snomed
5158005	Tourette's syndrome	Mental health	snomed
51637008	Chronic bipolar I disorder, most recent episode depressed	Mental health	snomed
51771007	Postviral fatigue syndrome	Physical illness	snomed
51795009	Musculoskeletal torsion, function (observable entity)	Physical illness	snomed
51868009	Ulcer of duodenum	Physical illness	snomed
51885006	Morning sickness	Physical illness	snomed
52011008	Injury of finger	Accident and injury	snomed
52254009	Nephrotic syndrome	Physical illness	snomed
52329006	Fracture, open	Accident and injury	snomed
52404001	Mastoiditis	Physical illness	snomed
52515009	Abdominal hernia	Physical illness	snomed
52603002	Closed fracture of lower limb	Accident and injury	snomed
52684005	Assault	Accident and injury	snomed
52702003	Chronic fatigue syndrome	Physical illness	snomed
52756005	Polyp in anterior nares	Physical illness	snomed
52845002	Nephritis	Physical illness	snomed
52899007	Partial thickness burn of lower limb	Accident and injury	snomed
5291005	Hypocalcaemia	Physical illness	snomed
52954000	Schizoid personality disorder	Mental health	snomed
53057004	Hand pain	Physical illness	snomed
53080007	Partial thickness burn of upper limb	Accident and injury	snomed
53084003	Bacterial pneumonia	Physical illness	snomed
53181008	Scratch marks	Suicidal behaviours and self−harm	snomed
53226007	Talipes planus	Physical illness	snomed
53277000	Vulvovaginitis	Physical illness	snomed
53295002	Chronic otitis externa	Physical illness	snomed
5332004	Oculogyric crisis	Physical illness	snomed
53430007	Mastalgia	Physical illness	snomed
53627009	Closed fracture of the radius and ulna	Accident and injury	snomed
53726008	Acute conjunctivitis	Physical illness	snomed
53792000	Closed fracture of shaft of ulna	Accident and injury	snomed
54048003	Threatened miscarriage	Physical illness	snomed

ed_code	description	presentation_category	classification
54150009	Upper respiratory tract infection	Physical illness	snomed
54200006	Tonic−clonic seizure	Physical illness	snomed
54296007	Partial thickness burn of hand	Accident and injury	snomed
54355006	Intracranial injury, without skull fracture	Accident and injury	snomed
54398005	Acute upper respiratory infection	Physical illness	snomed
54404000	Cervical radiculopathy	Physical illness	snomed
54512006	Nonspecific immune reaction	Physical illness	snomed
54556006	Fracture of ulna	Accident and injury	snomed
54586004	Lower abdominal pain	Physical illness	snomed
54635001	Scald of skin	Accident and injury	snomed
54888009	Sprain of knee	Accident and injury	snomed
54975007	Partial thickness burn of face AND/OR head	Accident and injury	snomed
55042009	Contusion of ankle	Accident and injury	snomed
55300003	Muscle cramp	Physical illness	snomed
55342001	Neoplastic disease	Physical illness	snomed
55343006	Burn of back of hand	Accident and injury	snomed
55434001	Hydrocoele	Physical illness	snomed
55464009	Systemic lupus erythematosus	Physical illness	snomed
55584005	Embolism − lesion	Physical illness	snomed
55680006	Drug overdose	Alcohol and substance misuse	snomed
55874001	Fracture of neck of metacarpal bone	Accident and injury	snomed
55899000	Foreign body on external eye	Accident and injury	snomed
55993003	Unilateral obstructed inguinal hernia	Physical illness	snomed
56018004	Wheezing	Physical illness	snomed
56020001	Patellar tap	Physical illness	snomed
56038003	Staphylococcal infection	Physical illness	snomed
56051008	Ketoacidosis	Physical illness	snomed
56097005	Migraine without aura	Physical illness	snomed
56170001	Photopsia	Physical illness	snomed
56608008	Pain in wrist	Physical illness	snomed
5662003	Contusion of hand	Accident and injury	snomed
56710004	Epidermal burn of wrist	Accident and injury	snomed
568005	Tic disorder	Mental health	snomed
56863004	Closed fracture of orbit	Accident and injury	snomed
56882008	Anorexia nervosa	Mental health	snomed

ed_code	description	presentation_category	classification
56890008	Sexual assault victim	Accident and injury	snomed
56905009	Intestinal perforation	Physical illness	snomed
57019003	Verruca vulgaris	Physical illness	snomed
57044006	Bartholin's gland cyst	Physical illness	snomed
57182000	Nerve injury	Accident and injury	snomed
57194009	Adjustment disorder with depressed mood	Mental health	snomed
57302007	Hypersensitivity reaction, caused by correct medicinal substance properly administered	Physical illness	snomed
57406009	Carpal tunnel syndrome	Physical illness	snomed
57419008	Gastroenteritis presumed infectious	Physical illness	snomed
57463004	Pneumonitis due to inhaled liquid	Physical illness	snomed
57546000	Acute severe asthma	Physical illness	snomed
5758002	Bacteraemia	Physical illness	snomed
57676002	Arthralgia	Physical illness	snomed
57773001	Rectal prolapse	Physical illness	snomed
58075000	Contusion of toe	Accident and injury	snomed
58077008	Hypotensive syncope	Physical illness	snomed
58126003	Postoperative wound infection	Physical illness	snomed
58150001	Fracture of clavicle	Accident and injury	snomed
58170007	Viral meningitis	Physical illness	snomed
58188004	Traumatic arthropathy	Accident and injury	snomed
58189007	Injury of anterior neck	Accident and injury	snomed
58196009	Abscess of ankle	Physical illness	snomed
58214004	Schizophrenia	Mental health	snomed
58320001	Traumatic dislocation of knee joint	Accident and injury	snomed
58580000	Closed supracondylar fracture of humerus	Accident and injury	snomed
58703003	Postpartum depression	Mental health	snomed
5888003	Keratitis	Physical illness	snomed
59021001	Angina decubitus	Physical illness	snomed
59026006	Blepharospasm	Physical illness	snomed
5913000	Fracture of neck of femur	Accident and injury	snomed
59274003	Intentional drug overdose	Suicidal behaviours and self−harm	snomed
59282003	Pulmonary embolism	Physical illness	snomed
59292006	Hemiplegic migraine	Physical illness	snomed
59363009	Inevitable miscarriage	Physical illness	snomed
59369008	Accidental drug overdose	Alcohol and substance misuse	snomed

ed_code	description	presentation_category	classification
59426001	Burn of vagina	Accident and injury	snomed
59843005	Furuncle	Physical illness	snomed
59883002	Cellulitis of abdominal wall	Physical illness	snomed
59966007	Structure of wisdom tooth	Physical illness	snomed
60046008	Pleural effusion	Physical illness	snomed
60119000	Exhaustion	Physical illness	snomed
60168000	Osteomyelitis	Physical illness	snomed
602001	Ross river fever	Physical illness	snomed
60342002	Movement disorder	Physical illness	snomed
60442001	Perforation of tympanic membrane	Physical illness	snomed
60505005	Congenital anomaly of optic disc	Physical illness	snomed
60540006	Cellulitis of external cheek	Physical illness	snomed
60555002	Hypersensitivity angiitis	Physical illness	snomed
6066007	Carbuncle of anus	Physical illness	snomed
60667009	Closed fracture of rib	Accident and injury	snomed
60713008	Burn of neck	Accident and injury	snomed
60728008	Abdominal swelling	Physical illness	snomed
6077001	Foot−drop	Physical illness	snomed
60814007	Antisocial behaviour	Mental health	snomed
60843004	Closed fracture of multiple bones of upper limb	Accident and injury	snomed
60845006	Dyspnoea on exertion	Physical illness	snomed
60862001	Tinnitus	Physical illness	snomed
60897004	Contusion of nose	Accident and injury	snomed
60959003	Open wound of fingernail with complication	Accident and injury	snomed
6118003	Demyelinating disease of central nervous system	Physical illness	snomed
6132001	Burn of thigh	Accident and injury	snomed
61372001	Aggressive behaviour	Mental health	snomed
61387006	Moderate anxiety	Mental health	snomed
6142004	Influenza	Physical illness	snomed
61486003	Sacral back pain	Physical illness	snomed
61582004	Allergic rhinitis	Physical illness	snomed
61653009	Bennett's fracture	Accident and injury	snomed
62014003	Adverse drug reaction	Physical illness	snomed
6215006	Acute cholangitis	Physical illness	snomed
62315008	Diarrhoea	Physical illness	snomed

ed_code	description	presentation_category	classification
62507009	Pins and needles	Physical illness	snomed
62660000	Conjunctival deposit	Physical illness	snomed
6273006	Priapism	Physical illness	snomed
62745008	Injury of ulnar nerve	Accident and injury	snomed
62837005	Cellulitis of hand	Physical illness	snomed
6284004	Abscess of neck	Physical illness	snomed
6285003	Tachyarrhythmia	Physical illness	snomed
63102001	Visual disturbance	Physical illness	snomed
6341002	Burn any degree involving less than 10 percent of body surface	Accident and injury	snomed
63643000	Derangement of knee	Physical illness	snomed
63649001	Cannabis delusional disorder	Mental health	snomed
6383007	Premature labour	Physical illness	snomed
63901009	Orchidodynia	Physical illness	snomed
63943002	Superficial injury of eyelid AND/OR periocular area	Accident and injury	snomed
64109004	Costal chondritis	Physical illness	snomed
6417001	Medial malleolus	Physical illness	snomed
64226004	Colitis	Physical illness	snomed
64229006	Traumatic lesion during delivery	Physical illness	snomed
64269007	Visual hallucinations	Mental health	snomed
64298006	Acquired deformity of finger due to trauma	Accident and injury	snomed
6456007	Supraventricular tachycardia	Physical illness	snomed
64576003	Abscess of buttock	Physical illness	snomed
64613007	Enteritis of small intestine	Physical illness	snomed
64662007	Pulmonary infarction	Physical illness	snomed
64665009	Closed fracture of calcaneus	Accident and injury	snomed
6471006	Suicidal thoughts	Suicidal behaviours and self−harm	snomed
64766004	Ulcerative colitis	Physical illness	snomed
64831005	Abrasion and/or friction burn of shoulder with infection	Physical illness	snomed
64905009	Paranoid schizophrenia	Mental health	snomed
6500006	Partial thickness burn of trunk	Accident and injury	snomed
65074000	Iritis	Physical illness	snomed
65108000	Potential for violence	Mental health	snomed
65120008	Generalised convulsive epilepsy	Physical illness	snomed
65124004	Swelling	Physical illness	snomed
65155005	Grand mal	Physical illness	snomed

ed_code	description	presentation_category	classification
6525002	Dependent drug abuse	Alcohol and substance misuse	snomed
65275009	Acute cholecystitis	Physical illness	snomed
65363002	Otitis media	Physical illness	snomed
65467000	Epidermal burn of forearm	Accident and injury	snomed
65568007	Cigarette smoker	Alcohol and substance misuse	snomed
65636009	Keratoconus	Physical illness	snomed
65759007	Injury of mouth	Accident and injury	snomed
65896005	Crushing injury of ankle	Accident and injury	snomed
65966004	Fracture of forearm	Accident and injury	snomed
66091009	Congenital disease	Physical illness	snomed
66112004	Closed fracture of sternum	Accident and injury	snomed
66123000	Ulcer on tongue	Physical illness	snomed
66190008	Victim of trauma with multiple injuries	Accident and injury	snomed
66214007	Substance abuse	Alcohol and substance misuse	snomed
66264000	Todd's paresis	Physical illness	snomed
66308002	Fracture of humerus	Accident and injury	snomed
66381006	Adjustment disorder with mixed disturbance of emotions AND conduct	Mental health	snomed
66383009	Gingivitis	Physical illness	snomed
6655004	Acute laryngitis	Physical illness	snomed
66590003	Alcohol dependence	Alcohol and substance misuse	snomed
66609003	Tactile hallucinations	Mental health	snomed
66657009	Paroxysmal ventricular tachycardia	Physical illness	snomed
66754008	Appendix	Physical illness	snomed
66760008	Optic neuritis	Physical illness	snomed
66857006	Haemoptysis	Physical illness	snomed
6698000	Closed trimalleolar fracture	Accident and injury	snomed
67070005	Crushing injury of ear	Accident and injury	snomed
67195008	Acute stress disorder	Mental health	snomed
6738008	Female infertility	Physical illness	snomed
67426006	Toxic effect of alcohol	Alcohol and substance misuse	snomed
67564005	Social maladjustment	Other	snomed
67602004	Acute peritonitis	Physical illness	snomed
67624004	Abscess of Bartholin's gland	Physical illness	snomed
67667007	Acute endometritis	Physical illness	snomed
67678004	Acute atopic conjunctivitis	Physical illness	snomed

ed_code	description	presentation_category	classification
67801009	Tenosynovitis	Physical illness	snomed
67878002	Abscess of upper arm	Physical illness	snomed
68010000	Ligament of knee joint	Physical illness	snomed
68142008	Contusion of upper limb	Accident and injury	snomed
68172002	Disorder of tendon	Physical illness	snomed
68226007	Acute cystitis	Physical illness	snomed
68267002	Idiopathic intracranial hypertension	Physical illness	snomed
68272006	Acute maxillary sinusitis	Physical illness	snomed
6836001	Injury of brachial plexus	Accident and injury	snomed
68566005	Urinary tract infection	Physical illness	snomed
68581004	Hypoglycaemia of childhood	Physical illness	snomed
68653001	Anal pain	Physical illness	snomed
68670009	Contact dermatitis of eyelid	Physical illness	snomed
68730006	Plastic operation of hoof	Other	snomed
68854005	Closed fracture of head of radius	Accident and injury	snomed
68890003	Schizoaffective disorder	Mental health	snomed
68962001	Myalgia	Physical illness	snomed
68978004	Hyperventilation	Mental health	snomed
68996008	Disorder of middle ear	Physical illness	snomed
69322001	Psychotic disorder	Mental health	snomed
69479009	Anxiety hyperventilation	Mental health	snomed
69776003	Acute gastroenteritis	Physical illness	snomed
69866009	Closed fracture of vault of skull without intracranial injury	Accident and injury	snomed
69878008	Polycystic ovaries	Physical illness	snomed
69916004	Fracture of base of thumb	Accident and injury	snomed
70070008	Torticollis	Mental health	snomed
7011001	Hallucinations	Mental health	snomed
70153002	Haemorrhoids	Physical illness	snomed
70176004	Foreign body in rectum	Accident and injury	snomed
70204006	Closed fracture of fifth metatarsal bone	Accident and injury	snomed
70250009	Epidermal burn of lower limb	Accident and injury	snomed
70273001	Paracetamol poisoning	Suicidal behaviours and self−harm	snomed
70323002	Eye strain	Physical illness	snomed
70505006	Partial thickness burn of forearm	Accident and injury	snomed
70582006	Scar of skin	Accident and injury	snomed

ed_code	description	presentation_category	classification
70637004	Cellulitis of toe	Physical illness	snomed
70704007	Sprain of wrist	Accident and injury	snomed
70759006	Pyoderma	Physical illness	snomed
708038006	Acute exacerbation of asthma	Physical illness	snomed
7093002	Renal colic	Physical illness	snomed
70997004	Mild anxiety	Mental health	snomed
71103003	Chronic residual schizophrenia	Mental health	snomed
71139009	Closed fracture of proximal end of radius	Accident and injury	snomed
71186008	Croup	Physical illness	snomed
712823008	Acute depression	Mental health	snomed
71286001	Spinal cord compression	Physical illness	snomed
71392009	Cellulitis of wrist	Physical illness	snomed
71555008	Closed fracture of ulna	Accident and injury	snomed
71620000	Fracture of femur	Accident and injury	snomed
7163005	Urinary tract obstruction	Physical illness	snomed
71677004	Effect of exposure to external cause	Accident and injury	snomed
7180009	Meningitis	Physical illness	snomed
71850005	Abdominal pain worse on motion	Physical illness	snomed
71906005	Paronychia	Physical illness	snomed
7200002	Alcoholism	Alcohol and substance misuse	snomed
72073003	Injury of flank	Accident and injury	snomed
721104000	Sepsis due to urinary tract infection	Physical illness	snomed
722051000168101	Situational crisis	Mental health	snomed
72274001	Nerve root disorder	Physical illness	snomed
72366004	Eating disorder	Mental health	snomed
7248001	Salicylate poisoning	Suicidal behaviours and self−harm	snomed
72605008	Candidal vulvovaginitis	Physical illness	snomed
72658003	Heat rash	Physical illness	snomed
72704001	Fracture	Accident and injury	snomed
72779005	Anorectal fistula	Physical illness	snomed
72892002	Normal pregnancy	Physical illness	snomed
72934000	Candidiasis of vagina	Physical illness	snomed
73009009	Bloodshot eyes	Physical illness	snomed
73013002	Disorder of cranial nerve	Physical illness	snomed
73211009	Diabetes mellitus	Physical illness	snomed

ed_code	description	presentation_category	classification
73430006	Sleep apnoea	Physical illness	snomed
735645009	Contusion of head	Accident and injury	snomed
73589001	Intervertebral disc prolapse	Physical illness	snomed
73595000	Stress	Mental health	snomed
736004	Abscess of hip	Physical illness	snomed
73790007	Threatened miscarriage in first trimester	Physical illness	snomed
73820008	Testicular dysfunction	Physical illness	snomed
73830004	Foreign body in penis	Accident and injury	snomed
73862001	Complication of catheter	Physical illness	snomed
73867007	Severe major depression with psychotic features	Mental health	snomed
73998008	Prolapse of female genital organs	Physical illness	snomed
74036000	Pregnancy detection examination	Physical illness	snomed
74188005	Medical	Physical illness	snomed
74323005	Pain in elbow	Physical illness	snomed
74333002	Spasmodic torticollis	Physical illness	snomed
74390002	Wolff−Parkinson−White pattern	Physical illness	snomed
74395007	Open fracture of phalanx of foot	Accident and injury	snomed
74400008	Appendicitis	Physical illness	snomed
74474003	Gastrointestinal haemorrhage	Physical illness	snomed
7449006	Injury of femoral nerve	Accident and injury	snomed
74506000	Bereavement	Mental health	snomed
74615001	Tachycardia−bradycardia	Physical illness	snomed
74682007	Crushing injury of toe	Accident and injury	snomed
74699008	Foreign body in nose	Accident and injury	snomed
74732009	Mental illness	Mental health	snomed
74779009	Strain of rotator cuff of shoulder	Accident and injury	snomed
74814004	Contusion of foot	Accident and injury	snomed
74934004	Psychoactive substance−induced withdrawal syndrome	Alcohol and substance misuse	snomed
75137002	Closed traumatic dislocation of joint of finger	Accident and injury	snomed
75183008	Abnormal liver function	Physical illness	snomed
7520000	Pyrexia of unknown origin	Physical illness	snomed
75258004	Foodborne illness	Physical illness	snomed
75308009	Closed fracture of navicular bone of foot	Accident and injury	snomed
75408008	Feeling angry	Mental health	snomed
75441006	Foreign body in ear	Accident and injury	snomed

ed_code	description	presentation_category	classification
75478009	Toxicity	Physical illness	snomed
7551007	Closed fracture of neck of metacarpal bone	Accident and injury	snomed
75544000	Opioid dependence	Alcohol and substance misuse	snomed
75591007	Fracture of fibula	Accident and injury	snomed
75694006	Pancreatitis	Physical illness	snomed
75705005	Red eye	Physical illness	snomed
75817003	Cellulitis of breast	Physical illness	snomed
75857000	Fracture of radius AND ulna	Accident and injury	snomed
75884004	Bleeding internal haemorrhoids	Physical illness	snomed
75955007	Thrombosed haemorrhoids	Physical illness	snomed
76046001	Ulcer of penis	Physical illness	snomed
76126009	Abscess of eyelid	Physical illness	snomed
76226003	Tattoo	Accident and injury	snomed
763287008	Review at hospital	Other	snomed
763288003	Patient review	Other	snomed
76349003	Extrapyramidal disease	Physical illness	snomed
76437000	Contusion of thumb	Accident and injury	snomed
76581006	Cholecystitis	Physical illness	snomed
76618002	Urethral stricture	Physical illness	snomed
76682005	Disorder of vitreous body	Physical illness	snomed
7674000	Greater trochanteric pain syndrome	Physical illness	snomed
76844004	Wound infection	Accident and injury	snomed
76865005	Closed fracture of distal phalanx of finger	Accident and injury	snomed
76902006	Tetanus	Physical illness	snomed
76974008	Fracture of multiple sites of phalanges of hand	Accident and injury	snomed
77054009	Cellulitis of thigh	Physical illness	snomed
77157004	Disorder of optic nerve	Physical illness	snomed
77295000	Closed skull fracture without intracranial injury	Accident and injury	snomed
77355000	Cannabis−induced organic mental disorder	Mental health	snomed
77386006	Pregnant	Physical illness	snomed
77477000	Computerised tomography	Other	snomed
77489003	Pterygium	Physical illness	snomed
77493009	Fracture of pelvis	Accident and injury	snomed
77568009	Back	Physical illness	snomed
77675002	Anorexia nervosa, restricting type	Mental health	snomed

ed_code	description	presentation_category	classification
77721001	Opioid intoxication	Alcohol and substance misuse	snomed
77803008	Closed fracture of shaft of fibula	Accident and injury	snomed
77830003	Epidermal burn of multiple sites	Accident and injury	snomed
77880009	Rectal pain	Physical illness	snomed
78004001	Bulimia nervosa	Mental health	snomed
78048006	Candidiasis	Physical illness	snomed
78164000	Feeding problem	Physical illness	snomed
78275009	Obstructive sleep apnoea	Physical illness	snomed
78292000	Fracture of shaft of metacarpal bone	Accident and injury	snomed
78370002	Scleritis	Physical illness	snomed
78408007	Complication of obstetrical surgery AND/OR procedure	Physical illness	snomed
78455002	Subjective visual disturbance	Physical illness	snomed
78514002	Thigh pain	Physical illness	snomed
78598000	Superficial injury of eye	Accident and injury	snomed
78623009	Endometritis	Physical illness	snomed
78691002	Staggering gait	Physical illness	snomed
78745000	Urticaria pigmentosa	Physical illness	snomed
78755001	Contact dermatitis due to cosmetics	Physical illness	snomed
78768009	Subconjunctival haemorrhage	Physical illness	snomed
78868004	Chronic mucoid otitis media	Physical illness	snomed
78883009	Hallux structure	Physical illness	snomed
7895008	Drug toxicity	Physical illness	snomed
78980006	Open fracture of lower end of forearm	Accident and injury	snomed
7899002	Feeling of throat tightness	Physical illness	snomed
79017007	Deformed pupil	Physical illness	snomed
79099006	Colitis presumed infectious	Physical illness	snomed
79216009	Abscess of groin	Physical illness	snomed
7927006	Periorbital haematoma	Physical illness	snomed
79315005	Mechanical complication due to urethral indwelling catheter	Physical illness	snomed
79626009	Closed fracture of talus	Accident and injury	snomed
79631006	Absence seizure	Physical illness	snomed
7973008	Abnormal vision	Physical illness	snomed
79740000	Candidiasis of mouth	Physical illness	snomed
79883001	Ovarian cyst	Physical illness	snomed
79890006	Loss of appetite	Physical illness	snomed

ed_code	description	presentation_category	classification
79893008	Vesicular rash	Physical illness	snomed
79899007	Medicine interaction	Other	snomed
79922009	Epigastric pain	Physical illness	snomed
79962008	Diffuse oesophageal spasm	Physical illness	snomed
80068009	Swelling of limb	Physical illness	snomed
80182007	Irregular menstruation	Physical illness	snomed
80313002	Palpitations	Physical illness	snomed
8034008	Painful penile erection	Physical illness	snomed
80394007	Hyperglycaemia	Physical illness	snomed
80423007	Spontaneous pneumothorax	Physical illness	snomed
80515008	Hepatomegaly	Physical illness	snomed
80583007	Severe anxiety (panic)	Mental health	snomed
80593000	Ingestion of foreign material	Accident and injury	snomed
80640009	Perirenal abscess	Physical illness	snomed
80744008	Commotio retinae	Physical illness	snomed
80756009	Closed fracture of patella	Accident and injury	snomed
80827001	Burn of forearm	Accident and injury	snomed
80967001	Dental caries	Physical illness	snomed
8098009	Sexually transmissible infection	Physical illness	snomed
81060008	Intestinal obstruction	Physical illness	snomed
81094005	Epidermal burn of thigh	Accident and injury	snomed
81102000	Back injury	Physical illness	snomed
81125004	Acute haemorrhagic cystitis	Physical illness	snomed
81302005	Worried well	Other	snomed
81308009	Encephalopathy	Physical illness	snomed
81371004	Concussion	Physical illness	snomed
81405006	Open wound of upper limb	Accident and injury	snomed
81485008	Worker in work−related accident	Accident and injury	snomed
815008	Episcleritis	Physical illness	snomed
81546003	Abscess of oral tissue	Physical illness	snomed
81576005	Closed fracture of phalanx of foot	Accident and injury	snomed
81629009	Traumatic dislocation of temporomandibular joint	Accident and injury	snomed
81639003	Closed fracture of nasal bones	Accident and injury	snomed
81680005	Neck pain	Physical illness	snomed
81712001	Pain in female genitalia on intercourse	Physical illness	snomed

ed_code	description	presentation_category	classification
81808003	Haemarthrosis	Accident and injury	snomed
81914009	Benzodiazepine−based tranquilliser poisoning	Alcohol and substance misuse	snomed
81935006	Family conflict	Other	snomed
81996005	Testicular torsion	Physical illness	snomed
82057004	Burn of finger	Accident and injury	snomed
82065001	Fracture of carpal bone	Accident and injury	snomed
82127005	Perianal abscess	Physical illness	snomed
82196007	Vascular insufficiency of intestine	Physical illness	snomed
82271004	Head injury	Accident and injury	snomed
82272006	Common cold	Physical illness	snomed
82276009	Antidepressant poisoning	Suicidal behaviours and self−harm	snomed
82346000	Acquired obstructive hydrocephalus	Accident and injury	snomed
82380002	Foreign body in intestine	Accident and injury	snomed
82423001	Chronic pain	Physical illness	snomed
82576008	Foreign body in eye	Accident and injury	snomed
8260003	Organophosphate poisoning	Suicidal behaviours and self−harm	snomed
82661006	Abdominal pregnancy	Physical illness	snomed
82675004	Synovial cyst of popliteal space	Physical illness	snomed
82782008	Ethanol poisoning	Alcohol and substance misuse	snomed
82966003	Hereditary angioedema	Physical illness	snomed
82991003	Generalised aches and pains	Physical illness	snomed
83074005	Unplanned pregnancy	Physical illness	snomed
83128009	Oliguria	Physical illness	snomed
83132003	Upper abdominal pain	Physical illness	snomed
83366000	Full thickness burns of multiple sites	Accident and injury	snomed
83458005	Agitated depression	Mental health	snomed
8349005	Sexual exposure	Physical illness	snomed
83746006	Chronic schizophrenia	Mental health	snomed
83966006	Hypothermia due to cold environment	Physical illness	snomed
840539006	COVID−19	Physical illness	snomed
840544004	Suspected COVID−19	Physical illness	snomed
840546002	Exposure to COVID−19	Physical illness	snomed
84178004	Conjunctival oedema	Physical illness	snomed
8420001	Abrasion procedure	Other	snomed
84229001	Fatigue	Physical illness	snomed

ed_code	description	presentation_category	classification
84292000	Menstrual period late	Physical illness	snomed
844005	Finding relating to behaviour	Mental health	snomed
84416003	Contusion of thigh	Accident and injury	snomed
84437006	Acute pneumothorax	Physical illness	snomed
84677008	Burn of lower limb	Accident and injury	snomed
84757009	Epilepsy	Physical illness	snomed
84849002	Tinea corporis	Physical illness	snomed
85005007	Cannabis dependence	Alcohol and substance misuse	snomed
8513005	Contusion of neck	Accident and injury	snomed
85189001	Acute appendicitis	Physical illness	snomed
85224001	Pilonidal cyst with abscess	Physical illness	snomed
85232009	Left ventricular failure	Physical illness	snomed
85243001	Minor laceration	Accident and injury	snomed
85478004	Disorder of iris	Physical illness	snomed
85561006	Uncomplicated alcohol withdrawal	Alcohol and substance misuse	snomed
85562004	Hand	Physical illness	snomed
85564003	Injury of blood vessels of head AND/OR neck	Accident and injury	snomed
85679001	Fracture, longitudinal	Accident and injury	snomed
85769006	Streptococcal infection	Physical illness	snomed
85777005	Dacryocystitis	Physical illness	snomed
85848002	Corneal abrasion	Accident and injury	snomed
85921004	Puncture procedure	Other	snomed
85922006	Fracture of hamate bone of wrist	Accident and injury	snomed
86208007	Loin pain−haematuria syndrome	Physical illness	snomed
86216003	Mastitis, associated with childbirth	Physical illness	snomed
86276007	Bleeding gums	Physical illness	snomed
86279000	Acute suppurative otitis media with spontaneous rupture of ear drum	Physical illness	snomed
8635005	Alcohol withdrawal delirium	Alcohol and substance misuse	snomed
86481000	Opening of peritoneum	Physical illness	snomed
86569001	Postpartum state	Physical illness	snomed
86708008	Seborrhea	Physical illness	snomed
86772005	Petroleum product poisoning	Alcohol and substance misuse	snomed
86849004	Suicidal deliberate poisoning	Suicidal behaviours and self−harm	snomed
86981007	Infective otitis externa	Physical illness	snomed
87132004	Opioid withdrawal	Alcohol and substance misuse	snomed

ed_code	description	presentation_category	classification
87317003	Respiratory arrest	Physical illness	snomed
87376003	Closed fracture of shaft of clavicle	Accident and injury	snomed
87414006	Reactive depression (situational)	Mental health	snomed
87486003	Aphasia	Physical illness	snomed
87522002	Iron deficiency anaemia	Physical illness	snomed
87642003	Dislocation	Accident and injury	snomed
8765009	Haematemesis	Physical illness	snomed
87778004	Sprain of hand	Accident and injury	snomed
87858002	Drug−related disorder	Alcohol and substance misuse	snomed
87860000	Testicular mass	Physical illness	snomed
88151007	Keratoconjunctivitis	Physical illness	snomed
88157006	Salpingitis	Physical illness	snomed
88361008	Cellulitis of groin	Physical illness	snomed
8837000	Amphetamine delirium	Alcohol and substance misuse	snomed
88594005	Herpes simplex	Physical illness	snomed
88797001	Complication of surgical procedure	Physical illness	snomed
88906006	Sprain of deltoid ligament of ankle	Accident and injury	snomed
88975006	Schizophreniform disorder	Mental health	snomed
89130002	Closed traumatic dislocation of fifth cervical vertebra	Accident and injury	snomed
89164003	Breast lump	Physical illness	snomed
89322006	Allergic urticaria due to drug	Physical illness	snomed
89362005	Weight loss	Physical illness	snomed
89627008	Hyponatraemia	Physical illness	snomed
89797005	Heat syncope	Physical illness	snomed
90129003	Tussive syncope	Physical illness	snomed
9014002	Psoriasis	Physical illness	snomed
90176007	Tonsillitis	Physical illness	snomed
90244007	Contusion of heel	Accident and injury	snomed
90275003	Habit	Other	snomed
90325002	Vomiting of pregnancy	Physical illness	snomed
90354008	Complication of internal device	Physical illness	snomed
90458007	Internal haemorrhoids	Physical illness	snomed
90460009	Neck injury	Accident and injury	snomed
90507008	Disorder of vein	Physical illness	snomed
90560007	Gout	Physical illness	snomed

ed_code	description	presentation_category	classification
9063003	Epidermal burn of back	Accident and injury	snomed
90688005	Chronic renal failure	Physical illness	snomed
90708001	Kidney disease	Physical illness	snomed
90739004	Thyrotoxicosis	Physical illness	snomed
90748009	Motorbike	Accident and injury	snomed
90774003	Victim of physical assault	Accident and injury	snomed
90834002	Pain in limb	Physical illness	snomed
90979004	Chronic tonsillitis	Physical illness	snomed
91019004	Paraesthesia	Physical illness	snomed
91038008	Acute frontal sinusitis	Physical illness	snomed
91146006	Epidermal burn of back of hand	Accident and injury	snomed
9115004	Pili incarnati	Physical illness	snomed
91175000	Seizure	Physical illness	snomed
9124008	Subacute appendicitis	Physical illness	snomed
91302008	Sepsis	Physical illness	snomed
91487003	Nappy rash	Physical illness	snomed
91514001	Corneal ulcer	Physical illness	snomed
91588005	Closed fracture of metacarpal bone	Accident and injury	snomed
91603007	Contusion of lower limb	Accident and injury	snomed
91613004	Contusion of elbow	Accident and injury	snomed
91862002	Acute periodontal abscess	Physical illness	snomed
91934008	Allergy to nut	Physical illness	snomed
91941002	Food anaphylaxis	Physical illness	snomed
91957002	Back pain complicating pregnancy	Physical illness	snomed
9209005	Acute abdominal pain syndrome	Physical illness	snomed
92507001	Buccal crossbite	Physical illness	snomed
9267009	Chest pain at rest	Physical illness	snomed
9275003	Multiple fractures of metacarpal bones	Accident and injury	snomed
93458008	Foreign body in skin	Accident and injury	snomed
9404003	Orbital swelling	Physical illness	snomed
9468002	Closed fracture of carpal bone	Accident and injury	snomed
95320005	Disorder of skin	Physical illness	snomed
95324001	Skin lesion	Physical illness	snomed
95344007	Ulcer of lower limb	Physical illness	snomed
95345008	Ulcer of foot	Physical illness	snomed

ed_code	description	presentation_category	classification
95434006	Tracheomalacia	Physical illness	snomed
95545007	Haemorrhagic diarrhoea	Physical illness	snomed
95566004	Urolithiasis	Physical illness	snomed
95570007	Renal calculus	Physical illness	snomed
95598005	Ruptured ovarian cyst	Physical illness	snomed
95644001	Systemic lupus erythematosus encephalitis	Physical illness	snomed
95668009	Pain in face	Physical illness	snomed
95674009	Ulnar neuropraxia	Physical illness	snomed
95725002	Corneal laceration	Accident and injury	snomed
95730003	Marginal keratitis	Physical illness	snomed
95766002	Disorder of lacrimal gland	Physical illness	snomed
95806007	Cellulitis of external ear	Physical illness	snomed
95808008	Infection of earlobe	Physical illness	snomed
95811009	Abscess of external auditory canal	Physical illness	snomed
95819006	Unilateral hearing loss	Physical illness	snomed
95820000	Bilateral hearing loss	Physical illness	snomed
95822008	Clicking tinnitus	Physical illness	snomed
95847005	Muscle injury	Accident and injury	snomed
95851007	Orbital fracture	Accident and injury	snomed
95855003	Traumatic amputation of finger	Accident and injury	snomed
95868006	Heat exhaustion	Physical illness	snomed
95880003	Soft tissue infection	Physical illness	snomed
95882006	Bacterial ear infection	Physical illness	snomed
95891005	Influenza−like illness	Physical illness	snomed
95898004	Tick bite	Physical illness	snomed
95913008	Drug action reversal	Alcohol and substance misuse	snomed
9655003	FB − Removal of foreign body from external auditory canal	Accident and injury	snomed
9682006	Fracture of scapula	Accident and injury	snomed
9713002	Prostatitis	Physical illness	snomed
9748009	Dyskinesia	Physical illness	snomed
9826008	Conjunctivitis	Physical illness	snomed
9991008	Abdominal colic	Physical illness	snomed
